# A biodiversity hotspot for Microgastrinae (Hymenoptera, Braconidae) in North America: annotated species checklist for Ottawa, Canada

**DOI:** 10.3897/zookeys.633.10480

**Published:** 2016-11-17

**Authors:** Jose Fernandez-Triana, Caroline Boudreault, Joel Buffam, Ronald Mclean

**Affiliations:** 1 Canadian National Collection of Insects, Ottawa, Canada

**Keywords:** Microgastrinae, diversity, North America, Canada, Ottawa

## Abstract

Microgastrinae wasps (Hymenoptera, Braconidae) from the city of Ottawa and its surroundings (a 50-km radius circle, ~7,800 km^2^) were studied based on 1,928 specimens collected between 1894 and 2010, and housed in the Canadian National Collection of Insects. A total of 158 species from 21 genera were identified, which is by far the highest number of species ever recorded for a locality in North America. An annotated checklist of species is provided. *Choeras parasitellae* (Bouché, 1834) and *Pholetesor nanus* (Reinhard, 1880) are recorded for the first time in the Nearctic (previously only known from the Palearctic region), *Cotesia depressa* (Viereck, 1912) is recorded for the first time in Canada (previously only known from the United States), and *Cotesia hemileucae* (Riley, 1881) and *Protapanteles phlyctaeniae* (Muesebeck, 1929) are recorded for the first time in the province of Ontario. In Ottawa the most diverse genera are *Cotesia*, *Apanteles*, *Microplitis*, *Pholetesor*, *Microgaster*, and *Dolichogenidea*, altogether comprising 77% of the species found in the area. A total of 73 species (46%) were represented by only one or two specimens, suggesting that the inventory for Ottawa is still relatively incomplete. Seasonal distribution showed several peaks of activity, in spring, summer, and early fall. That general pattern varied for individual species, with some showing a single peak of abundance either in the summer or towards the end of the season, others species attaining two peaks, in late spring and late summer, or in early summer and early fall, and yet others attaining up to three different peaks, in spring, summer and fall. At least 72 of the Microgastrinae species from Ottawa have been previously associated with 554 species of Lepidoptera as hosts – but those historical literature records are not always reliable and in many cases are based on data from areas beyond Ottawa. Thus, our knowledge of the associations between the 158 species of microgastrine parasitoids and the caterpillars of the 2,064 species of Lepidoptera recorded from Ottawa is still very incomplete.

## Introduction

Microgastrinae wasps are the second largest subfamily of Braconidae (Hymenoptera) (Yu et al. 2016), and evidence suggests they may eventually prove to be the largest subfamily (e.g., Rodriguez et al. 2013). The number of described species has increased significantly within the past 35 years: in 1980 there were about 1,500 (Papp 1976, Mason 1981), by 2012 there were more than 2,200 (Yu et al. 2012), and currently the total surpasses 2,700 (Yu et al. 2016). The actual diversity worldwide is estimated to be 20,000–46,000 species (Rodriguez et al. 2013), meaning that most likely 10% or even less of the extant species have been described.

At the regional or local level our knowledge about Microgastrinae is also very incomplete, with few biodiversity inventories available and most published information not being comprehensive enough to capture the actual diversity of species. Examples in North America include Lewis and Whitfield (1999), Whitfield and Lewis (2001), Shaw (2002), and Fernandez-Triana et al. (2009, 2011).

Besides its extraordinary diversity, microgastrine wasps are very important in biocontrol efforts against Lepidoptera pests, as they represent the single most important group of caterpillar parasitoids in the world (Whitfield 1997).

This paper analyzes the diversity of Microgastrinae of Ottawa (Canada) and the surrounding areas, discusses the significance of these results for future studies of the group in the Nearctic region, and provides an initial reference point for the Ottawa fauna (with a dynamic species checklist to be available online as well).

## Methods

**Figure 1.**
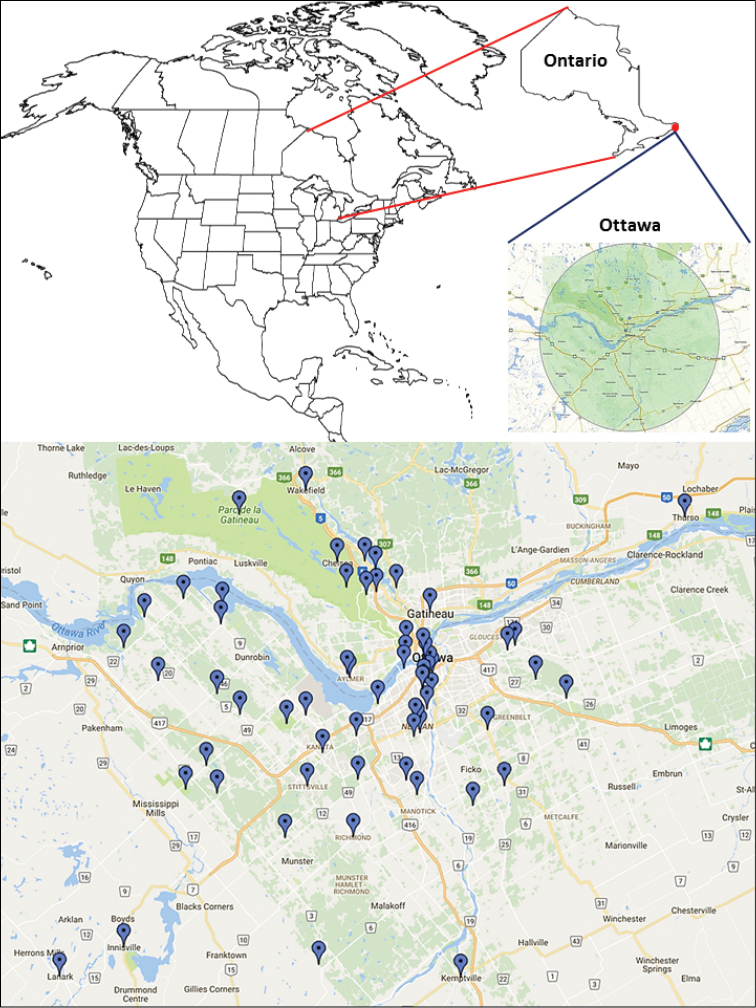
Localities within the city of Ottawa and surroundings where specimens of Microgastrinae were collected (1894–2010, data based on CNC holdings only).

Since 1895, the Ottawa Field-Naturalists’ Club (http://www.ofnc.ca/) has considered a circle with a 50 km radius (approximately 7,800 km^2^) centered on the Peace Tower in downtown Ottawa as its study area. This human-defined region (variously named as ‘National Capital Region’, ‘Ottawa-Gatineau Region’, ‘Ottawa Region’, ‘Ottawa District’) has been widely adopted and used in many scientific papers and conservation efforts conducted in the city over the past 120 years. We follow the same concept, henceforth calling it simply ‘Ottawa’ for brevity. Figure 1 shows the localities within this 50-km radius where specimens of Microgastrinae had been collected and were available to us for study. For consistency, we do not include data from a few additional species collected in localities slightly out of this 100 km diameter circle even though, from a natural perspective, those species are very likely to be found in Ottawa as well.

This paper is based on the study of 1,928 specimens, housed in the Canadian National Collection of Insects, Ottawa (CNC); they were all databased and assigned to species following the most recent taxonomic information available for Canada (Fernandez-Triana 2010, 2014, 2015, Fernandez-Triana et al. 2013, 2014a, 2014b, 2015). Some specimens could only be identified to genus but in all cases they had unique morphological characteristics and/or DNA barcodes that clearly identified them as distinct species–in those cases we use an alphanumeric species identifier e.g., ‘*Apanteles* jft09’. In order to allow these provisional species to be recognized and studied further in the future we also provide DNA Barcodes Index Numbers (BINs) (Ratnasingham and Hebert 2013) for them in the annotated species checklist.

Pictures of 36 species are provided to illustrate the diversity of microgastrine wasps in Ottawa. Photos were taken either with a Leica M165 C or a Keyence VHX-1000 Digital Microscope, using a lens with a range of 10–130 ×. Multiple images were taken of a structure through the focal plane and then combined to produce a single in-focus image. For the images taken with the Leica camera, the Zerene Stacker program (http://zerenesystems.com/cms/stacker) was used; software associated with the Keyence System produced focused images taken with that camera. Images were corrected using Adobe Photoshop CS4 and the plates were prepared using Microsoft PowerPoint 2010.

A species checklist was generated using the CNC database (http://www.cnc-ottawa.ca/taxonomy/TaxonMain.php). The list is organized alphabetically by genus and species within a given genus. For every taxon we detail general distribution (outside Ottawa), specimens examined, and notes on species where relevant. For zoogeographic regions we used the following acronyms: NEA-Nearctic, NEO-Neotropics, OTL-Oriental, and PAL-Palearctic.

Using the Scratchpad platform and infrastructure (Smith et al. 2011), the species checklist given here as the initial reference point for the Ottawa fauna of Microgastrinae will be made available online (http://microgastrinae.myspecies.info/content/microgastrinae-ottawa-canada), where updates will be maintained as and when changes occur.

## Results

A total of 158 species from 21 genera of Microgastrinae are recorded from Ottawa. *Choeras parasitellae* (Bouché, 1834) and *Pholetesor nanus* (Reinhard, 1880) are new records for the Nearctic (previously only known from the Palearctic region). *Cotesia depressa* (Viereck, 1912) is a new record for Canada (previously only known from the United States). *Cotesia hemileucae* (Riley, 1881) and *Protapanteles phlyctaeniae* (Muesebeck, 1929) are new records for Ontario.

The most diverse genera were *Cotesia* (32 species), *Apanteles* (31), *Microplitis* (23), *Pholetesor* (13), *Microgaster* (11), and *Dolichogenidea* (11). These six genera together comprised 77% of the species found in the area. This pattern is similar to that found in both the whole Nearctic region (Whitfield 1995, Yu et al. 2012, 2016) as well as in specific North American localities that have been the focus of biodiversity inventories (Lewis and Whitfield 1999, Whitfield and Lewis 2001, Shaw 2002, Fernandez-Triana et al. 2009, 2011). A notable exception is *Glyptapanteles*, one of the most diverse genera in North America and elsewhere, but currently with only three species recorded from Ottawa. However, many species of that genus still remain unidentified and further study will certainly reveal a much higher diversity for *Glyptapanteles* in the area. Five genera of Microgastrinae reached their northernmost known distribution in Ottawa: *Alphomelon*, *Clarkinella*, *Distatrix*, *Protomicroplitis* and *Pseudapanteles* (Fernandez-Triana 2010, 2014, 2015, Fernandez-Triana et al. 2014c).

At species level, the most common were *Glyptapanteles militaris* (Walsh, 1861) (322 specimens), *Pholetesor ornigis* (Weed, 1887) (157), *Glyptapanteles pallipes* (Reinhard, 1880) (87), *Apanteles nephoptericis* (Packard, 1864) (82), *Cotesia atalantae* (Packard, 1881) (77), *Microplitis varicolor* Viereck, 1917 (72), *Hypomicrogaster zonaria* (Say, 1836) (65), *Dolichogenidea cacoeciae* (Riley, 1881) (59), *Hygroplitis melligaster* (Provancher, 1886) (47), and *Cotesia laeviceps* (Ashmead, 1890) (45). Those ten species together represented 53% of all Microgastrinae specimens collected in Ottawa.

There were 50 species (31.4%) represented by only one specimen, and 23 (14.4%) represented by two specimens. The high proportion (45.9%) of singletons and doubletons shows how incomplete the Ottawa species inventory still is. The Chao-1 non-parametric estimator of species richness (Rarefaction Calculator at http://www.biology.ualberta.ca/jbrzusto/rarefact.php#Calculator) estimates 213.35 ± 16.29 species for Ottawa, thus our present knowledge represents less than 75% of the actual total of species to be found there.

Among all listed species, 73 were only identified to genus and have been assigned interim alphanumeric identifiers. Some of them certainly represent new taxa to be described in future papers. Others may represent species already described in Europe and for the present are given interim identifiers until they are compared with Palearctic material.

Regarding zoogeographical affinities, 132 species (83%) found in Ottawa are strictly Nearctic, 16 species (10%) are distributed within the Holarctic, 5 (3%) are recorded from the New World (Nearctic and Neotropics), and 10 (6%) are either truly cosmopolitan or present in at least three different regions (Nearctic/Neotropics/Palearctic or Nearctic/Oriental/Palearctic).

Some of the localities sampled contain special, sometimes unique habitats/ecosystems. Gatineau Park, with a rich biodiversity and a wide range of protected habitats and ecosystems within its 36,000 ha (http://www.ncc-ccn.gc.ca/places-to-visit/gatineau-park/conservation-gatineau-park) contains five species so far found nowhere else within the Ottawa area: *Alphomelon winniewertzae* Deans, 2003, *Cotesia depressa* (Viereck, 1912), *Cotesia diacrisiae* (Gahan, 1917), *Distatrix carolinae* Fernandez-Triana, 2010, and *Microplitis impressus* (Wesmael, 1837).

Mer Bleue, a 3,500 ha conservation area with a northern, boreal-like ecosystem more typical of the Subarctic than the Ottawa Valley (http://www.ncc-ccn.gc.ca/places-to-visit/greenbelt/mer-bleue) harbours three species that have not been found elsewhere in the Ottawa area: *Cotesia clisiocampae* (Ashmead, 1903), *Dolichogenidea absona* (Muesebeck, 1965), and *Pholetesor rhygoplitoides* Whitfield, 2006. Similarly, the approximately 1,500 ha of alvar ecosystem near Almonte (Belcher et al. 1992) is the only known Ottawa-area locality for *Pholetesor viminetorum* (Wesmael, 1837). Further studies on the fauna of Microgastrinae may show that some of those species are more widely distributed within Ottawa or beyond, though some are likely to remain restricted to particular habitats or ecosystems where their hosts occur.

The diversity of Microgastrinae revealed for Ottawa is extraordinary: in spite of being a relatively small area (7,800 km^2^) its species total would rank 17^th^ among countries of the world (Table 1). And, when placed within the context of what has been published for other, similarly-sized localities in North America, Ottawa has the highest number of species, double the second most diverse locality (Table 2). Even when accounting for differences in collecting efforts and taxonomic coverage (e.g., it is clear that the relatively low diversity in the Yellowstone National Park is due to insufficient study of its Microgastrinae fauna), the total recorded in Ottawa may not be surpassed by any North American locality at a comparable latitude. The high diversity found in Ottawa likely relates to being a transition from an eastern deciduous forest biome to a boreal biome, with small areas of unusual habitats like dunes, alvars, floodplains, and bogs.

**Table 1.** Microgastrinae diversity by country with over 100 species recorded. Data from Taxapad (Yu et al. 2012), Fauna Europaea (http://www.fauna-eu.org/) and the present paper (for Canada and Ottawa). For Costa Rica the total is a very conservative estimate based on numerous papers published after 2012 on the fauna of Area de Conservación Guanacaste (http://janzen.sas.upenn.edu/caterpillars/database.lasso).

Costa Rica	400+	Finland	163
Hungary	328	**Ottawa**	**158**
China	316	Mongolia	157
Russia	293	Italy	150
U.S.A.	284	Ukraine	149
Germany	249	Yugoslavia	139
United Kingdom	234	Democratic Republic of Congo	134
India	210	Bulgaria	124
Canada	196	Korea	124
Czech Republic	190	France	123
Romania	173	Sweden	117
Poland	170	Moldova	113
Switzerland	168	Slovakia	113
Turkey	167	Australia	112
Netherlands	176	Kazakhstan	112

**Table 2.** Microgastrinae diversity in selected North American localities. Data from Lewis and Whitfield (1999), Whitfield and Lewis (2001), Shaw (2002), Fernandez-Triana et al. (2009, 2011), and the present paper.

Locality/ecosystem	Latitude	Species recorded
Yellowstone National Park, Montana/forests	44°N	35
Quebec/apple orchards	45°N	36
Midwestern USA/tall grass prairies	39°N	55
Arkansas/forests	34°N	65
Churchill, Manitoba/boreal forest/tundra	59°N	79
Ottawa, Ontario/diverse habitats	45°N	158

We are not aware of published information for more southern localities, although based on specimens seen in collections (Fernandez-Triana, unpublished), it is clear that southern localities in the United States, when thoroughly sampled and studied, should have higher species totals. But Ottawa presently is the biodiversity hotspot for Microgastrinae in North America, a result of its habitat diversity and the relatively comprehensive taxonomic studies done on its fauna.

**Figure 2.**
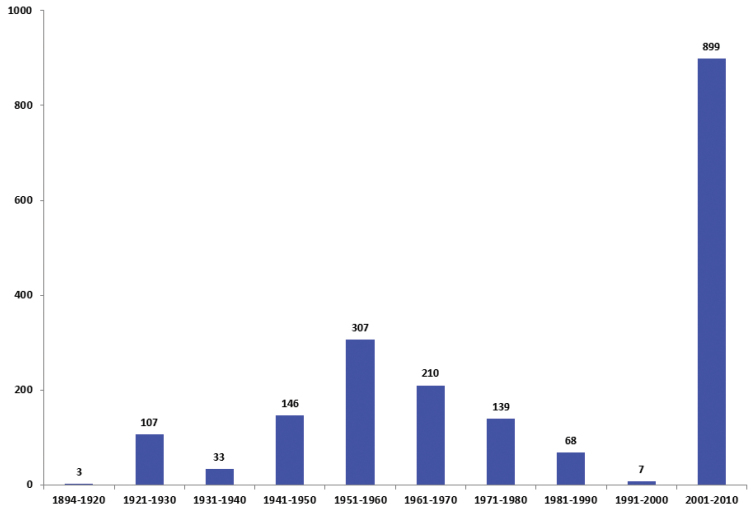
Specimens of Microgastrinae collected in Ottawa by decade (1894–2010). Data based on CNC holdings only.

The Ottawa microgastrines we studied were collected from 1894–2010 (Figure 2), with most of the specimens collected either from 1945–1975 or 2007–2008. More recent material (2012–2016) is presently being prepared and will be included in future updates of the current species list.

**Figure 3.**
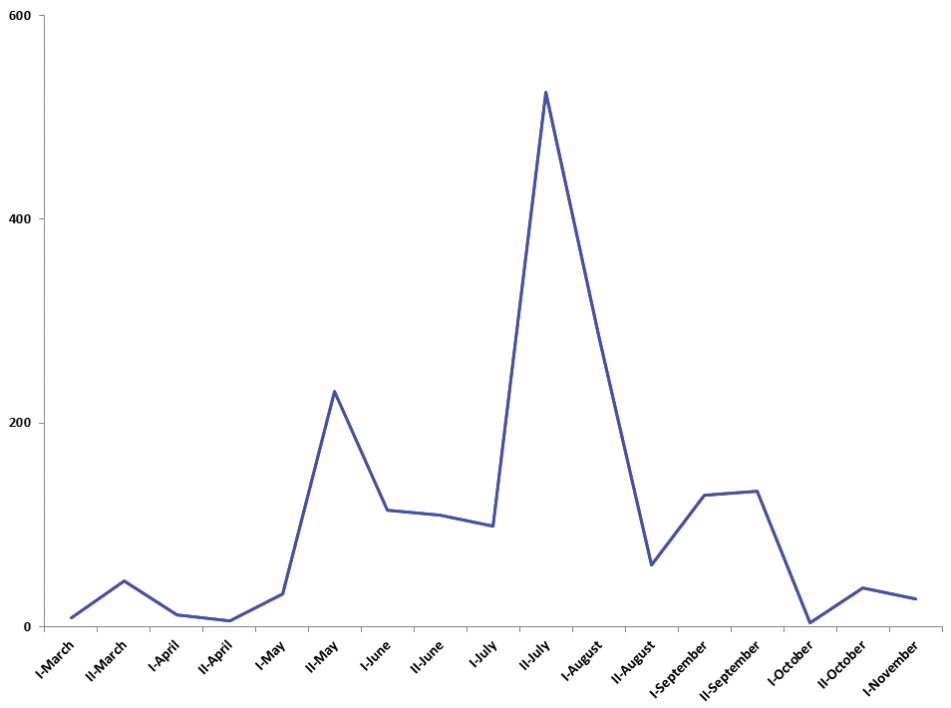
Specimens of Microgastrinae collected in Ottawa bi-weekly (March-November), based on CNC holdings only (1894–2010).

Seasonal distribution of the specimens in general (Figure 3) shows several activity peaks: 1) spring (second half of May to second half of June), 2) a larger peak in summer (late July to early August), and 3) a smaller peak in early fall (September). This pattern is somewhat similar to that found in another intensively studied Canadian locality (Churchill, Manitoba, at 59°N), although there were only two peaks in that more northern area, due to its shorter season (Fernandez-Triana et al. 2011).

**Figure 4.**
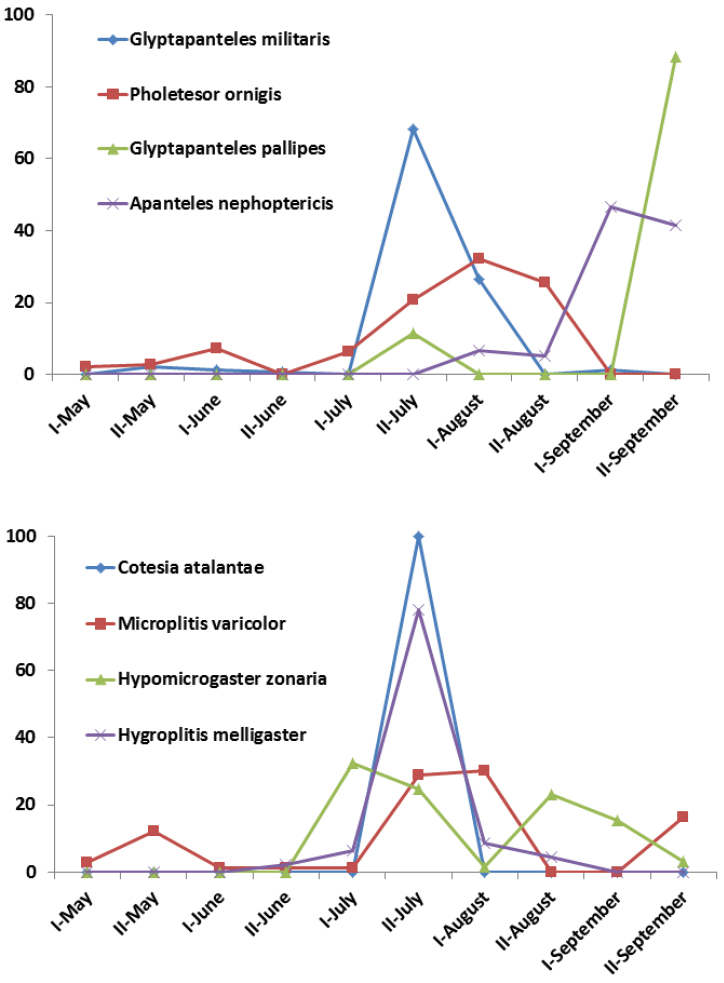
Bi-weekly (March-November) abundance of the eight most common species of Microgastrinae collected in Ottawa, based on CNC holdings only (1894–2010).

The general pattern varies depending on the species. Among the eight most common species, five show only a single peak of abundance, three in summer (*Glyptapanteles militaris*, *Cotesia atalantae* and *Hygroplitis melligaster*), and two in the fall (*Apanteles nephoptericis* and *Glyptapanteles pallipes*). Two species have two peaks, *Pholetesor ornigis* in late spring and late summer, and *Hypomicrogaster zonaria* in early summer and early fall. *Microplitis varicolor* has three peaks in spring, summer and fall (Fig. 4). As more collected material becomes available, a better picture of species seasonality will be obtained.

Species of Microgastrinae are parasitoids exclusively of Lepidoptera (Whitfield 1997). There are at least 2,064 species of Lepidoptera already recorded from Ottawa (D. Lafontaine, *in lit.* 2016, Lafontaine 1997; http://www.acleris.com/dls/habitat-diversity.html), with the actual number probably approaching 2,200 species (D. Lafontaine, pers. com.). A literature search shows that 72 of the Microgastrinae species found in Ottawa have been, at some point, recorded parasitizing at least 554 Lepidoptera species (based on data compiled by Yu et al. 2012).

However, it must be pointed that some of those lepidopterans do not actually occur in Ottawa, and some of the reported host/parasitoid associations are likely to be incorrect (see Shaw et al. 2009 for a discussion and examples that demonstrate the pitfalls of using compilations of host/parasitoid abstracted uncritically from the literature). Our knowledge of the association between lepidopteran caterpillars and their microgastrine parasitoids at the local and/or regional level is still very incomplete, and many more studies are needed before some patterns can be reliably established. Thus, in this paper we do not analyze further the relationships between Microgastrinae and Lepidoptera in Ottawa.

Evidence from recent studies in Area de Conservacion Guanacaste (ACG), Costa Rica, comprising 11,000+ Lepidoptera and 1,100+ Microgastrinae species (e.g., Janzen and Hallwachs 2016) shows that microgastrine wasps are more specialized (=host specific) than previously thought. Most species have been found parasitizing one/few species of Lepidoptera which are usually phylogenetically related or, alternatively, related ecologically in the niche they occupy (e.g., Fernandez-Triana et al. 2014d). At present there is no comprehensively enough information in North America to compare, but the available (more limited) data suggests that it might well be the same situation as in the tropics. Based on the currently verified and available data, the ratio between species of Lepidoptera (2,064) and Microgastrinae (158) in Ottawa stands at 13.1, very similar to the ratio found in ACG and other regions of world (Fernandez-Triana 2010, Rodriguez et al. 2013, Janzen and Hallwachs 2016). The significance of the Lepidoptera/Microgastrinae ratio has been discussed further in Rodriguez et al. (2013).

### Annotated checklist of Microgastrinae from Ottawa, Canada

#### Alphomelon winniewertzae

Taxon classificationAnimaliaHymenopteraBraconidae

Deans, 2003

Fig. 5

##### Distribution.

NEA, NEO.

##### Notes.

The status of this species as a potential member of the Species Candidate Lists of The Committee on the Status of Endangered Wildlife in Canada (COSEWIC, http://www.cosewic.gc.ca/) was assessed by Fernandez Triana (2014).

##### Material examined.

Quebec, Old Chelsea, 45.503548 -75.797963, 351m, 11.viii.1965, J. R. Vockeroth, Voucher Code: CNCHYM00025.

**Figure 5.**
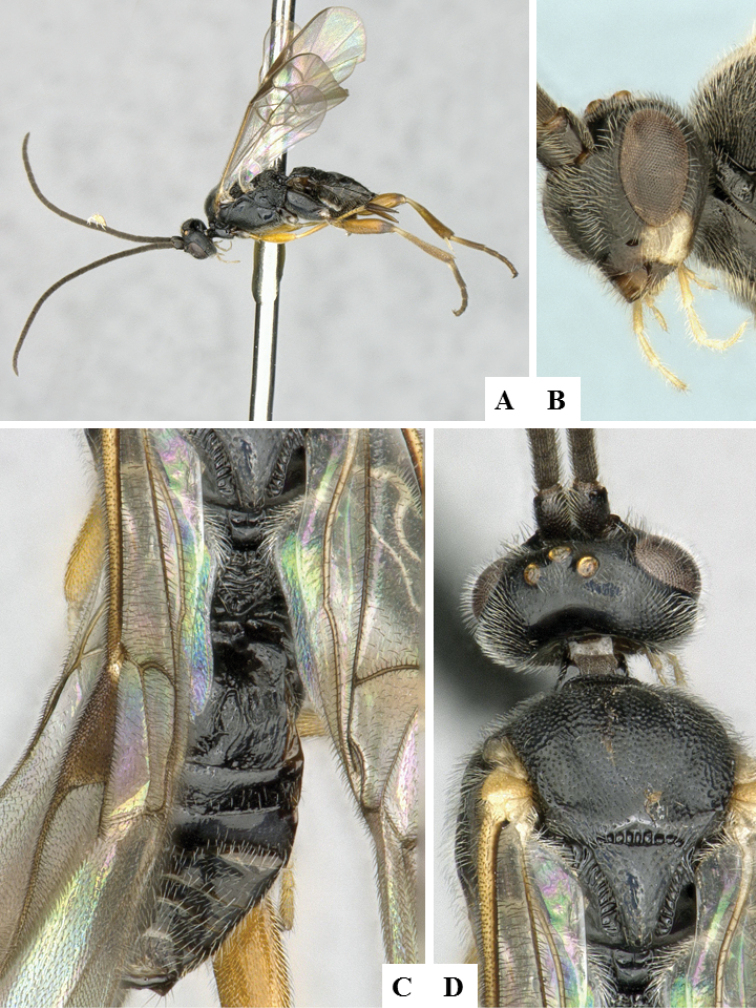
*Alphomelon winniewertzae*. **A** Habitus, lateral **B** Head, lateral **C** Metasoma, dorsal **D** Head and mesosoma (partially), dorsal.

#### Apanteles baldufi

Taxon classificationAnimaliaHymenopteraBraconidae

Muesebeck, 1968

##### Distribution.

NEA.

##### Material examined.

Ontario, Blackburn, 45.430272 -75.563017, 15.vi.1942, J. McDunnough, Voucher Code: MIC000024; 20.vi.1931, G.S. Walley, Voucher Code: MIC000020; Dirleton, Ont, 45.495069 -76.142986, 25.vi.1963, G.S. Walley, Voucher Code: CNC280541.

#### Apanteles canarsiae

Taxon classificationAnimaliaHymenopteraBraconidae

Ashmead, 1898

##### Distribution.

NEA.

##### Material examined.

Quebec, Wrightville, 45.438108 -75.743308, 23.vi.1939, F.I.S., Voucher Code: MIC000032.

#### Apanteles carpatus

Taxon classificationAnimaliaHymenopteraBraconidae

(Say, 1836)

##### Distribution.

Cosmopolitan.

##### Material examined.

Ontario, Ottawa, city garden, 45.356 -75.707, 10.viii.2007, H. Goulet, Voucher Code: CAM0036, CAM0042; 13.vii.2007, H. Goulet, Voucher Code: CAM0020, CAM0021, CAM0058, CAM0063; 19.ix.2007, H. Goulet, Voucher Code: CAM0023; 23.vii.2007, H. Goulet, Voucher Code: CAM0046, CAM0049, CAM0053; 30.vii.2007, H. Goulet, Voucher Code: CAM0026, CAM0117; 8.ix.2007, H. Goulet, Voucher Code: CAM0104; Ottawa, 45.356083 -75.706933, 12.iii.1952, J.R. Vockeroth, Voucher Code: CNC280555; 45.3825 -75.7137, 15.ix.1955, J. R. Vockeroth, Voucher Code: MIC000035; 45.382500 -75.713700, 8.v.1928, C. Twinn, Voucher Code: MIC000033; 45.406631 -75.701407, 4.iii.1952, J.R. Vockeroth , Voucher Code: CNC474712; v.1952, J.R. Vockeroth , Voucher Code: CNC474713.

#### Apanteles conanchetorum

Taxon classificationAnimaliaHymenopteraBraconidae

Viereck, 1917

##### Distribution.

NEA.

##### Material examined.

Ontario, Aylmer West, 45.400000 -75.850000, 20.vii.1972, Voucher Code: MIC000060; Ottawa, city garden, 45.356 -75.707, 1.ix.2007, H. Goulet, Voucher Code: CAM0147, CAM0159, CAM0160; 10.viii.2007, H. Goulet, Voucher Code: CAM0085; 19.ix.2007, H. Goulet, Voucher Code: CAM0005; 30.vii-10.viii.2007, H. Goulet, Voucher Code: CAM1013; Woodlawn, 45.375 -76.083, 6.viii.2008, L. Masner, Voucher Code: MIC000600.

#### Apanteles crassicornis

Taxon classificationAnimaliaHymenopteraBraconidae

(Provancher, 1886)

Fig. 6

##### Distribution.

NEA.

##### Material examined.

Ontario, Merivale, 45.325948 -75.719082, 17.viii.1930, J.J. de Gryse, Voucher Code: MIC000071; Ottawa, city garden, 45.356083 -75.706933, 1-19.ix.2007, H. Goulet, Voucher Code: CNC280561; 10.viii-1.ix.2007, H. Goulet, Voucher Code: MIC000067, MIC000069.

**Figure 6.**
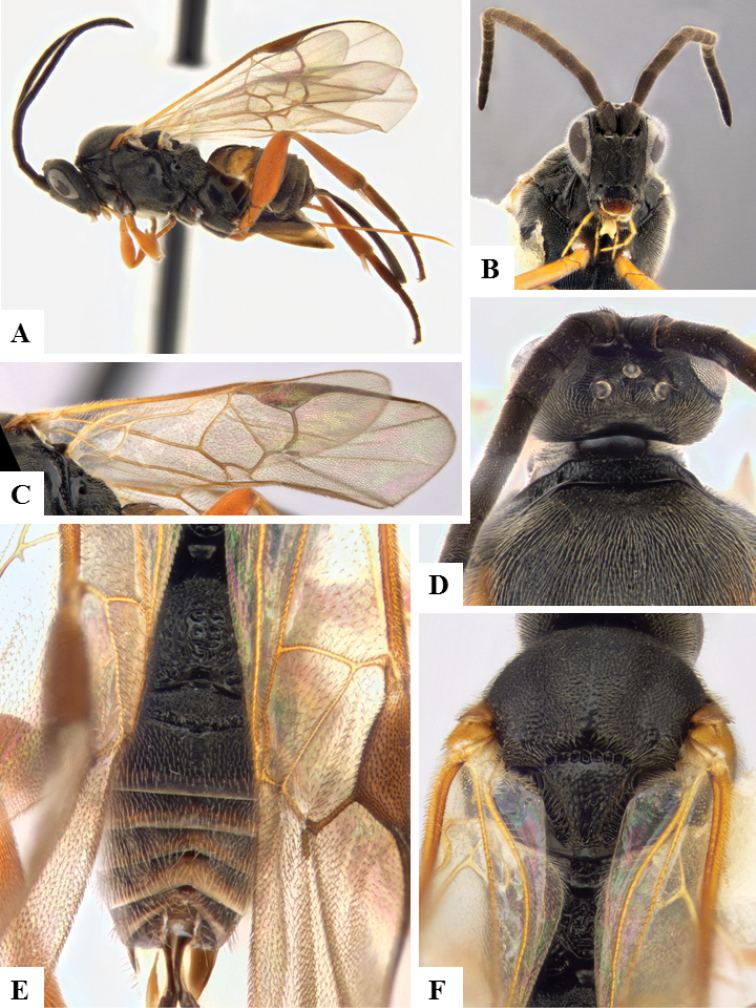
*Apanteles crassicornis*. **A** Habitus, lateral **B** Head, frontal **C** Wings **D** Head and mesosoma (partially), dorsal **E** Metasoma, dorsal **F** Mesosoma, dorsal.

#### Apanteles depressariae

Taxon classificationAnimaliaHymenopteraBraconidae

Muesebeck, 1931

##### Distribution.

NEA.

##### Material examined.

Ontario, Ottawa, Hogs Back, 45.372817 -75.698000, 11-17.vii.1951, C.D. Miller, Voucher Code: MIC000074; 45.372865 -75.697930, 11-16.vii.1951, Miller, C.O., Voucher Code: CNC280565; Quebec, Kirk’s Ferry, 45.541575 -75.817381, 24.v.1950, Bierne, Voucher Code: MIC000075.

#### Apanteles epinotiae

Taxon classificationAnimaliaHymenopteraBraconidae

Viereck, 1912

##### Distribution.

NEA.

##### Material examined.

Ontario, Ottawa, 45.406631 -75.701407, 22.vii.1954, W.R.M. Mason, Voucher Code: MIC000094.

#### Apanteles forbesi

Taxon classificationAnimaliaHymenopteraBraconidae

Viereck, 1910

##### Distribution.

NEA.

##### Material examined.

Ontario, Blackburn, 45.430270 -75.563015, 9.vi.1939, O. Peck, Voucher Code: MIC000100.

#### Apanteles fumiferanae

Taxon classificationAnimaliaHymenopteraBraconidae

Viereck, 1912

##### Distribution.

NEA, PAL.

##### Material examined.

Ontario, Ottawa, 45.356083 -75.706933, 23.vi.1945, F.I.S, Voucher Code: CNC280597; 45.3825 -75.7137, 23.vi.1945, Voucher Code: MIC000282; South March, 45.348507 -75.923123, 17.vi.1949, Voucher Code: MIC000272.

#### Apanteles jenniferae

Taxon classificationAnimaliaHymenopteraBraconidae

Fernandez-Triana, 2010

Fig. 7

##### Distribution.

NEA.

##### Material examined.

Ontario, Galetta, 45.433392 -76.250133, 28.vi.1951, Voucher Code: CNCHYM07234; Quebec, Old Chelsea, 45.503548 -75.797963, 19.vi.1940, Voucher Code: CNCHYM07232; Tenaga, 45.531020 -75.798359, 1.vii.1940, Voucher Code: CNCHYM07233.

**Figure 7.**
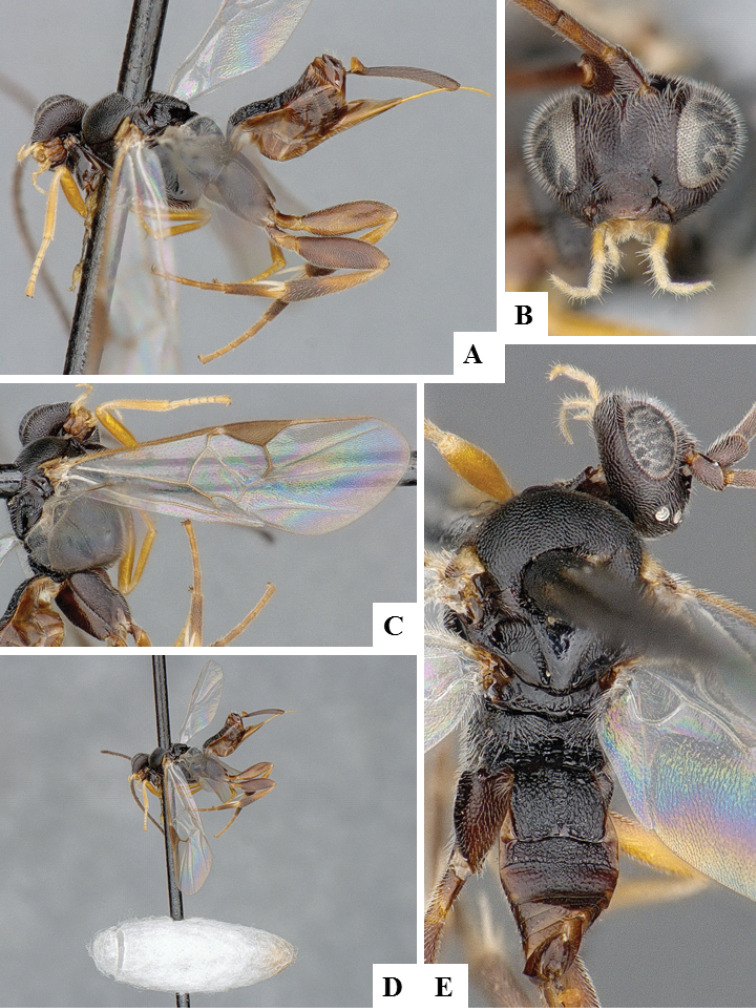
*Apanteles jenniferae*. **A** Habitus, lateral **B** Head, frontal **C** Wings **D** Adult wasp and its cocoon **E** Habitus, dorsal.

#### Apanteles laricellae

Taxon classificationAnimaliaHymenopteraBraconidae

Mason, 1959

Fig. 8

##### Distribution.

NEA.

##### Material examined.

Ontario, Kemptville Rideau, 45.016409 -75.646449, 21.v.1957, F.I.S., Voucher Code: MIC000120; 21.v.1957, F.I.S., Voucher Code: CNC474716; Mer Bleue, 45.393578 -75.512128, 1.vi.1960, C.D. Miller, Voucher Code: CNC280644; 45.393585 -75.512138, 6.vi.1960, C.D. Miller, Voucher Code: MIC000121; 7.vi.1960, C.D. Miller, Voucher Code: CNC474717; 45.393593 -75.512138, 1.vi.1960, C.D. Miller, Voucher Code: CNCHYM00142.

**Figure 8.**
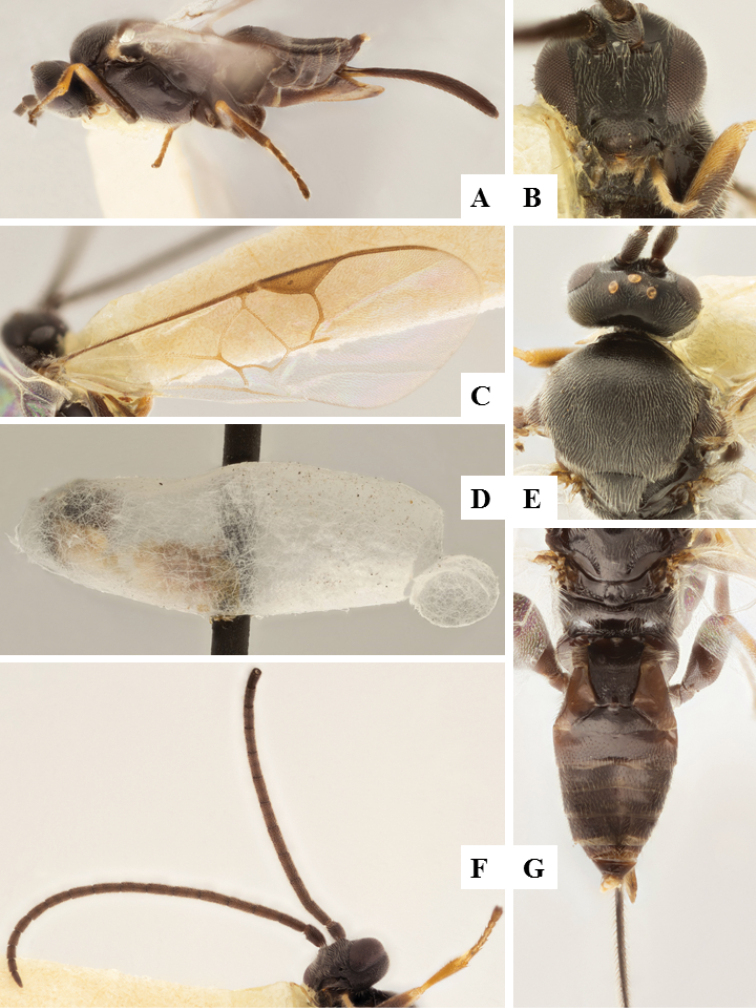
*Apanteles laricellae*. **A** Habitus, lateral **B** Head, frontal **C** Wings **D** Head and mesosoma (partially), dorsal **E** Metasoma, lateral **F** Metasoma, dorsal **F** Mesosoma, dorsal.

#### Apanteles morrisi

Taxon classificationAnimaliaHymenopteraBraconidae

Mason, 1974

Fig. 9

##### Distribution.

NEA, PAL.

##### Material examined.

Ontario, North Gower to Smith Falls, 1 km N of Rd 6 & Montague Bdy Rd, 45.033 -75.9, 15.vi.2004, Bennett & Barnes, Voucher Code: HYM00001061.

**Figure 9.**
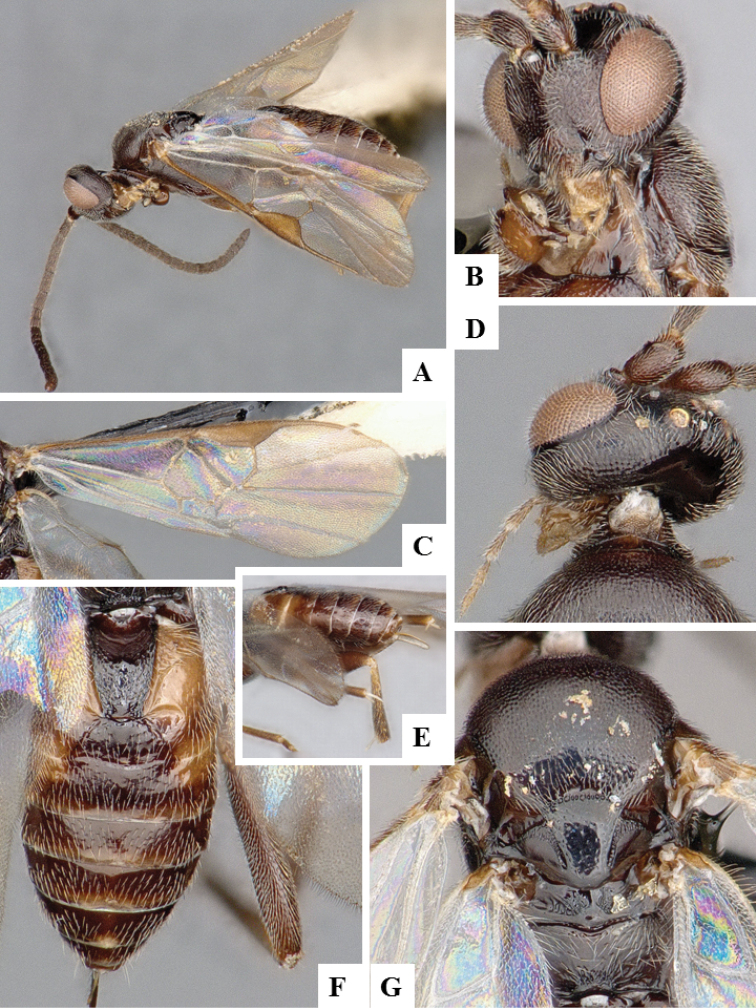
*Apanteles morrisi*. **A** Habitus, lateral **B** Head, frontal **C** Wings **D** Head and mesosoma, dorsal **E** Metasoma dorsal **F** Head and antenna, dorsal.

#### Apanteles nephoptericis

Taxon classificationAnimaliaHymenopteraBraconidae

(Packard, 1864)

##### Distribution.

NEA.

##### Material examined.

Ontario, Ottawa, 45.356083 -75.706933, 15.viii.1955, W. R. M. Mason, Voucher Code: CNC280686; 45.3825 -75.7137, 1.ix.1955, W. R. M. Mason, Voucher Code: CNCHYM00172; 15.viii.1955, W. R. M. Mason, Voucher Code: CNCHYM00173, MIC000151; 45.406631 -75.701407, 6.ix.1954, R. Lambert, Voucher Code: MIC000154, MIC000155; 7.ix.1955, W.R.M. Mason, Voucher Code: MIC000152, MIC000153; 1.ix.1955, W.R.M. Mason, Voucher Code: CNC474776-CNC474783; 15.viii.1955, W.R.M. Mason, Voucher Code: CNC474788, CNC474789, CNC474790; 17.viii.1955, W.R.M. Mason, Voucher Code: CNC474784, CNC474785, CNC474786, CNC474787; 20.ix.1955, W.R.M. Mason, Voucher Code: CNC474718-CNC474748, CNC474791; 6.ix.1955, W.R.M. Mason, Voucher Code: CNC474763, CNC474764, CNC474765, CNC474766, CNC474767, CNC474768, CNC474769, CNC474770, CNC474771, CNC474772, CNC474773, CNC474774, CNC474775; 7.ix.1955, W.R.M. Mason, Voucher Code: CNC474749, CNC474750, CNC474751, CNC474752, CNC474753, CNC474754, CNC474755, CNC474756, CNC474757, CNC474758, CNC474759, CNC474760, CNC474761, CNC474762.

#### Apanteles petrovae

Taxon classificationAnimaliaHymenopteraBraconidae

Walley, 1937

Fig. 10

##### Distribution.

NEA, PAL.

##### Material examined.

Ontario, Constance Bay, 45.486218 -76.073461, 14.v.1946, F.I.S. No.4-19, Voucher Code: CNC474792; 45.486248 -76.073504, 16.v.1934, G.S. Walley, Ottawa, city garden, 45.3561 -75.7069, 5.v-5.vi.2008, H. Goulet, Voucher Code: CAM1000, CAM1002, CAM1006, CAM1008, CAM1009; 45.3561 -75.707, 26.vi.2007, H. Goulet, Voucher Code: CAM0073; 30.v.2007, H. Goulet, Voucher Code: CAM0019.

**Figure 10.**
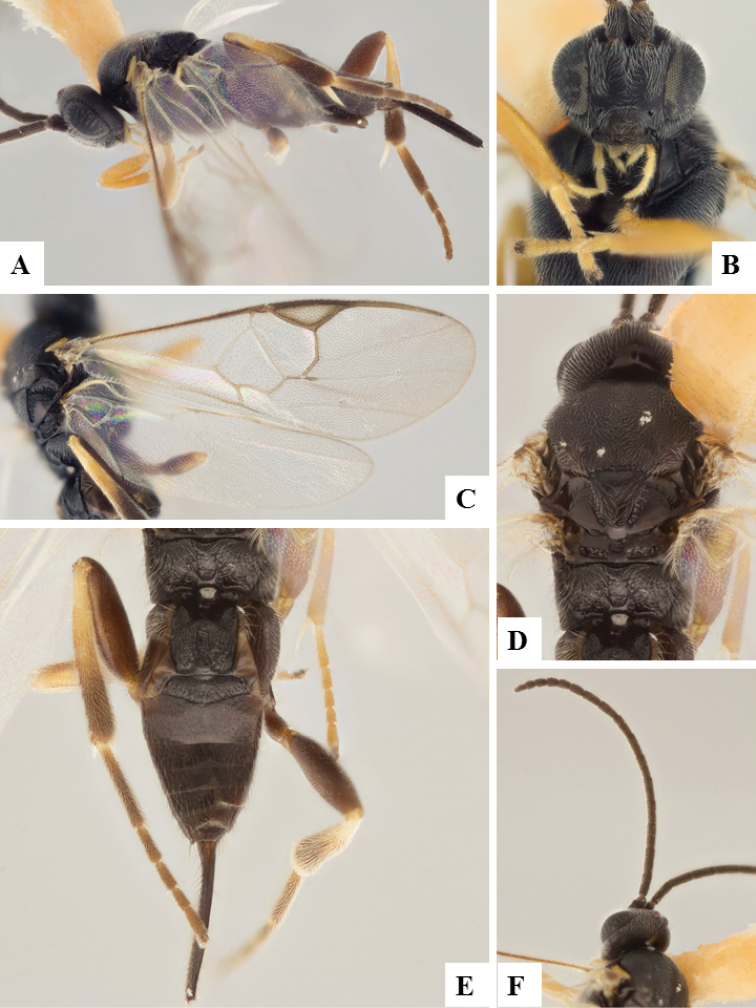
*Apanteles petrovae*. **A** Habitus, lateral **B** Head, frontal **C** Wings **D** Cocoon **E** Head and mesosoma (partially), dorsal **F** Head and antenna, ventrally **G** Metasoma, dorsal.

#### Apanteles polychrosidis

Taxon classificationAnimaliaHymenopteraBraconidae

Viereck, 1912

Fig. 11

##### Distribution.

NEA.

##### Material examined.

Quebec, Aylmer, 45.4 -75.85, 10.vii.2004, S. Laplante, Voucher Code: HYM00001646.

**Figure 11.**
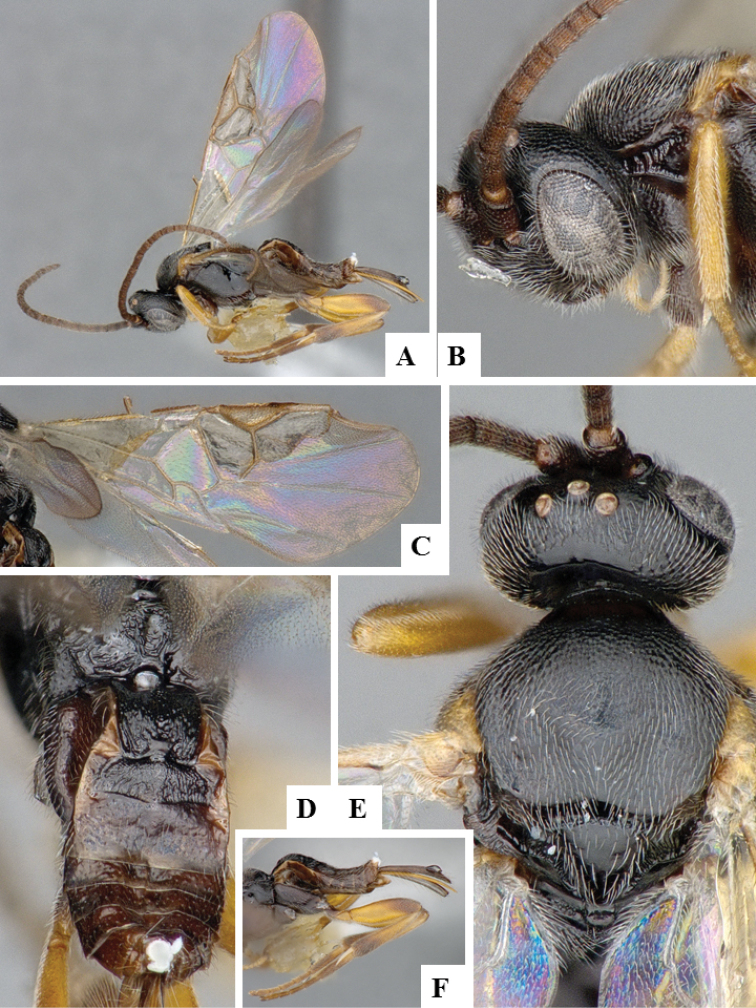
*Apanteles polychrosidis*. **A** Habitus, lateral **B** Head, lateral **C** Wings **D** Metasoma, dorsal **E** Head and mesosoma (partially), dorsal **F** Metasoma, lateral.

#### Apanteles jft01

Taxon classificationAnimaliaHymenopteraBraconidae

##### Distribution.

NEA.

##### Notes.

This species corresponds in BOLD to BIN BOLD:AAA6373, with specimens found across Canada and US.

##### Material examined.

Ontario, 5 km NW of Almonte, Hwy 49, Burnt Land, Alvar Prov. Park, Almonte, 45.2549 -76.14, 29.v.2008, Goulet & Fernandez, Voucher Code: CAM0353, CAM0357, CAM0361, CAM0364; Ottawa, city garden, 45.3561 -75.707, 2.vii.2007, H. Goulet, Voucher Code: CAM0128; 30.v.2007, H. Goulet, Voucher Code: CAM0012, CAM0017; Woodlawn, 45.375 -76.083, 6.viii.2008, L. Masner, Voucher Code: MIC000599, MIC000601.

#### Apanteles jft04

Taxon classificationAnimaliaHymenopteraBraconidae

##### Distribution.

NEA.

##### Material examined.

Ontario, Ottawa, city garden, 45.3561 -75.707, 15.vii.2007, H. Goulet, Voucher Code: CAM0123.

#### Apanteles jft09

Taxon classificationAnimaliaHymenopteraBraconidae

##### Distribution.

NEA.

##### Notes.

This species corresponds in BOLD to BIN BOLD:AAA8872, with all specimens collected in southern Ontario.

##### Material examined.

Ontario, Ottawa, city garden, 45.3561 -75.7069, 28.vii.2009, L. Masner, Voucher Code: CNCH1024; 30.vii-10.viii.2007, H. Goulet, Voucher Code: CAM1014; 45.3561 -75.707, 1.ix.2007, H. Goulet, Voucher Code: CAM0080, CAM0156, CAM0162; 10.viii.2007, H. Goulet, Voucher Code: CAM0032, CAM0035, CAM0037; 13.vii.2007, H. Goulet, Voucher Code: CAM0064; 16.vi.2007, H. Goulet, Voucher Code: CAM0111; 19.ix.2007, H. Goulet, Voucher Code: CAM0006, CAM0010; 23.vii.2007, H. Goulet, Voucher Code: CAM0045, CAM0047, CAM0052; 26.vi.2007, H. Goulet, Voucher Code: CAM0066, CAM0067; 30.vii.2007, H. Goulet, Voucher Code: CAM0028; 8.ix.2007, H. Goulet, Voucher Code: CAM0107; 45.356100 -75.707000, 8.ix.2007, H. Goulet, Voucher Code: CAM0102.

#### Apanteles jft10

Taxon classificationAnimaliaHymenopteraBraconidae

##### Distribution.

NEA.

##### Notes.

This species corresponds in BOLD to BIN BOLD:AAA8871, with all specimens collected in North America.

##### Material examined.

Ontario, 5 km NW of Almonte, Hwy 49, Burnt Land, Alvar Prov. Park, Almonte, 45.2549 -76.14, 29.v.2008, Goulet & Fernandez, Voucher Code: CAM0334, CAM0336, CAM0337, CAM0338, CAM0339, CAM0354, CAM0358, CAM0359.

#### Apanteles jft12

Taxon classificationAnimaliaHymenopteraBraconidae

##### Distribution.

NEA.

##### Notes.

This species corresponds in BOLD to BIN BOLD:AAB8748, with all specimens collected in Canada.

##### Material examined.

Ontario, Island Park, Ottawa, 45.407035 -75.747564, 14.x.2008, L. Masner, Voucher Code: MIC000603; Ottawa, city garden, 45.3561 -75.7069, 24-30.v.2007, H. Goulet, Voucher Code: CAM0872; 45.3561 -75.707, 26.vi.2007, H. Goulet, Voucher Code: CAM0068, CAM0069, CAM0070, CAM0071; 30.v.2007, H. Goulet, Voucher Code: CAM0018; 8.vi.2007, H. Goulet, Voucher Code: CAM0065.

#### Apanteles jft14

Taxon classificationAnimaliaHymenopteraBraconidae

##### Distribution.

NEA.

##### Notes.

This species corresponds in BOLD to BIN BOLD:AAD2306, with all specimens collected in eastern Canada.

##### Material examined.

Ontario, Ottawa, city garden, 45.3561 -75.707, 1.ix.2007, H. Goulet, Voucher Code: CAM0043, CAM0090, CAM0148.

#### Apanteles jft15

Taxon classificationAnimaliaHymenopteraBraconidae

##### Distribution.

NEA.

##### Notes.

This species corresponds in BOLD to BIN BOLD:ACL9543, with all specimens collected in North America.

##### Material examined.

Ontario, Ottawa, city garden, 45.3561 -75.7069, 1-19.ix.2007, H. Goulet, Voucher Code: CAM0871; 45.3561 -75.707, 19.ix.2007, H. Goulet, Voucher Code: CAM0003.

#### Apanteles jft25

Taxon classificationAnimaliaHymenopteraBraconidae

##### Distribution.

NEA.

##### Notes.

This species corresponds in BOLD to BIN BOLD:AAA9844, with all specimens collected found in Ottawa.

##### Material examined.

Ontario, Ottawa, city garden, 45.3561 -75.707, 1.ix.2007, H. Goulet, Voucher Code: CAM0077, CAM0078, CAM0079, CAM0081, CAM0086, CAM0088, CAM0089, CAM0152, CAM0155, CAM0157, CAM0158, CAM0164, CAM0165, CAM0167, CAM0168; 10.viii.2007, H. Goulet, Voucher Code: CAM0029, CAM0031, CAM0033, CAM0034, CAM0038, CAM0040, CAM0116; 13.vii.2007, H. Goulet, Voucher Code: CAM0059; 15.vii.2007, H. Goulet, Voucher Code: CAM0121, CAM0125; 30.vii.2007, H. Goulet, Voucher Code: CAM0027, CAM0097, CAM0118, CAM0119; 45.356100 -75.707000, 10.viii.2007, H. Goulet, Voucher Code: CAM0041; Woodlawn, 45.375 -76.083, 17.viii.2008, L. Masner, Voucher Code: MIC000602; 8.ix.2008, L. Masner, Voucher Code: MIC000604.

#### Apanteles jft26

Taxon classificationAnimaliaHymenopteraBraconidae

##### Distribution.

NEA.

##### Notes.

This species corresponds in BOLD to BIN BOLD:AAA8770, with all specimens collected in North America.

##### Material examined.

Ontario, 5 km NW of Almonte, Hwy 49, Burnt Land, Alvar Prov. Park, Almonte, 45.2549 -76.14, 29.v.2008, Goulet & Fernandez, Voucher Code: CAM0363; Ottawa, city garden, 45.3561 -75.707, 1.ix.2007, H. Goulet, Voucher Code: CAM0083; 30.v.2007, H. Goulet, Voucher Code: CAM0014, CAM0016.

#### Apanteles jft27

Taxon classificationAnimaliaHymenopteraBraconidae

##### Distribution.

NEA.

##### Notes.

This species corresponds in BOLD to BIN BOLD:ACF4409, with all specimens collected in southern Ontario.

##### Material examined.

Ontario, Ottawa, city garden, 45.3561 -75.707, 1.ix.2007, H. Goulet, Voucher Code: CAM0150; 10.viii.2007, H. Goulet, Voucher Code: CAM0030; 13.vii.2007, H. Goulet, Voucher Code: CAM0055; 15.vii.2007, H. Goulet, Voucher Code: CAM0122, CAM0126; 23.vii.2007, H. Goulet, Voucher Code: CAM0050; Woodlawn, 45.375 -76.083, 8.ix.2008, L. Masner, Voucher Code: MIC000605.

#### Apanteles jft28

Taxon classificationAnimaliaHymenopteraBraconidae

##### Distribution.

NEA.

##### Notes.

This species corresponds in BOLD to BIN BOLD:AAF7782, with all specimens collected in eastern Canada.

##### Material examined.

Ontario, Ottawa, city garden, 45.3561 -75.707, 15.vii.2007, H. Goulet, Voucher Code: CAM0127.

#### Apanteles jft31

Taxon classificationAnimaliaHymenopteraBraconidae

##### Distribution.

NEA, PAL.

##### Notes.

This species corresponds in BOLD to BIN BOLD:AAB0096, with specimens collected in the Nearctic (Canada, US), and the Palearctic (Russia).

##### Material examined.

Ontario, mixed forest, 45.2347 -75.624, 19-29.vi.2007, A. Bennett, Voucher Code: CAM0533; 7-19.vi.2007, A. Bennett, Voucher Code: CAM0535, CAM0536; Ottawa, city garden, 45.3561 -75.7069, 16-26.vi.2007, H. Goulet, Voucher Code: CAM0898; 24-30.v.2007, H. Goulet, Voucher Code: CAM0873; 45.3561 -75.707, 13.vii.2007, H. Goulet, Voucher Code: CAM0056; 15.vii.2007, H. Goulet, Voucher Code: CAM0124; 16.vi.2007, H. Goulet, Voucher Code: CAM0105, CAM0108, CAM0109, CAM0110, CAM0112; 2.vii.2007, H. Goulet, Voucher Code: CAM0129, CAM0130, CAM0131, CAM0132, CAM0133, CAM0134, CAM0135, CAM0136, CAM0137, CAM0138, CAM0139, CAM0140, CAM0141, CAM0142, CAM0143, CAM0144, CAM0145; 26.vi.2007, H. Goulet, Voucher Code: CAM0075.

#### Apanteles jft37

Taxon classificationAnimaliaHymenopteraBraconidae

##### Distribution.

NEA.

##### Notes.

This species corresponds in BOLD to BIN BOLD:AAC3220, with all specimens collected in North America.

##### Material examined.

Ontario, Ottawa, city garden, 45.3561 -75.7069, 23.vii.2009, L. Masner, Voucher Code: CNCH0459; 45.3561 -75.707, 1.ix.2007, H. Goulet, Voucher Code: CAM0082; 13.vii.2007, H. Goulet, Voucher Code: CAM0061; 23.vii.2007, H. Goulet, Voucher Code: CAM0051; 30.vii.2007, H. Goulet, Voucher Code: CAM0098; Woodlawn, 45.375 -76.083, 6.viii.2008, L. Masner, Voucher Code: MIC000598.

#### Apanteles sp. 1 nr conanchetorum

Taxon classificationAnimaliaHymenopteraBraconidae

##### Distribution.

NEA.

##### Notes.

This species is morphologically close to *Apanteles conanchetorum* Viereck, 1917 but DNA barcodes are different (BINS: BOLD:AAC5506 and BOLD:AAC5507) and it has been considered as a separate species by Fernandez-Triana et al. (2014).

##### Material examined.

Ontario, Ottawa city, Slack road, 45.321539 -75.730767, 5.x.2007, L. Masner, Voucher Code: CAM0096.

#### Apanteles sp.1 nr ensiger

Taxon classificationAnimaliaHymenopteraBraconidae

##### Distribution.

NEA.

##### Notes.

The specimens of ‘*Apanteles ensiger* (Say, 1836)’ that have rendered DNA barcodes comprise two BINS (BOLD:ACE6783 and BOLD:AAA3764) and have been considered as separate species by Fernandez-Triana et al. (2014).

##### Material examined.

Ontario, 2 km SW of Innisville, 45.054942 -76.250619, 26.vi.1991, Sharkey & Read, Voucher Code: GOU0303; 5 km NW of Almonte, Hwy 49, Burnt Land, Alvar Prov. Park, Almonte, 45.255 -76.14, 29.v.2008, Goulet & Fernandez, Voucher Code: CAM0335, CAM0348; Blackburn, 45.436469 -75.549278, 9.vi.1939, O. Peck, Voucher Code: MIC000083; Britannia, Ottawa, 45.362878 -75.794003, 20.vi.1947, G. Shewell, Voucher Code: MIC000082; mixed forest near Manotick, Ottawa, 45.235 -75.624, 19-29.vi.2007, A. Bennett, Voucher Code: CAM0521, CAM0522; 29.vi-16.vii.2007, A. Bennett, Voucher Code: CAM0525, CAM0526, CAM0527, CAM0528, CAM0529, CAM0530, CAM0531; 7-19.vi.2007, A. Bennett, Voucher Code: CAM0523, CAM0524; North Gower to Smith Falls, 1 km N of Rd 6 & Montague Bdy Rd, 45.033 -75.9, 15.vi.2004, Bennett & Barnes, Voucher Code: HYM00001003; Ottawa, city garden, 45.356 -75.707, 1.ix.2007, H. Goulet, Voucher Code: CAM0154, CAM0161; 10.viii.2007, H. Goulet, Voucher Code: CAM0114; 13.vii.2007, H. Goulet, Voucher Code: CAM0022; 19.ix.2007, H. Goulet, Voucher Code: CAM0009; 26.vi.2007, H. Goulet, Voucher Code: CAM0074; 30.vii.2007, H. Goulet, Voucher Code: CAM0024, CAM0025; Quebec, Gatineau Park, 45.600556 -76.042647, 15.vi.1977, L. Masner, Voucher Code: CNC474715; 45.600572 -76.042647, 15.vi.1977, L. Masner, Voucher Code: MIC000090.

#### Apanteles sp.2 nr ensiger

Taxon classificationAnimaliaHymenopteraBraconidae

##### Distribution.

NEA.

##### Notes.

The specimens of ‘*Apanteles ensiger* (Say, 1836)’ that have rendered DNA barcodes comprise two BINS (BOLD:ACE6783 and BOLD:AAA3764) and have been considered as separate species by Fernandez-Triana et al. (2014).

##### Material examined.

Ontario, 5 km NW of Almonte, Hwy 49, Burnt Land, Alvar Prov. Park, Almonte, 45.255 -76.14, 29.v.2008, Goulet & Fernandez, Voucher Code: CAM0340, CAM0341, CAM0342, CAM0344, CAM0345, CAM0346, CAM0347, CAM0349, CAM0350, CAM0351, CAM0352, CAM0356, CAM0360, MIC000666; Ottawa, city garden, 45.356 -75.707, 1.ix.2007, H. Goulet, Voucher Code: CAM0146, CAM0151, CAM0163, CAM0166; 10.viii.2007, H. Goulet, Voucher Code: CAM0039, CAM0113, CAM0115; 19.ix.2007, H. Goulet, Voucher Code: CAM0007, CAM0008; 23.vii.2007, H. Goulet, Voucher Code: CAM0054; 26.vi.2007, H. Goulet, Voucher Code: CAM0062, CAM0072, CAM0076; 30.v.2007, H. Goulet, Voucher Code: CAM0011, CAM0013; 30.vii.2007, H. Goulet, Voucher Code: CAM0120; 6.vii.2009, L. Masner, Voucher Code: CNCH1025; 8.ix.2007, H. Goulet, Voucher Code: CAM0099, CAM0103, CAM0106; 45.356000 -75.707000, 8.ix.2007, H. Goulet, Voucher Code: CAM0101.

#### Choeras consimilis

Taxon classificationAnimaliaHymenopteraBraconidae

(Viereck, 1911)

Fig. 12

##### Distribution.

NEA.

##### Material examined.

Ontario, Ottawa, 45.3825 -75.7137, 1.ix.1952, J. F. McAlpine, Voucher Code: CNCHYM00278; Quebec, Gatineau Park, 45.600555 -76.042647, 13.vi.1966, D.P. Pielou, Voucher Code: CNC474797; 14.vii.1966, D.P. Pielou, Voucher Code: CNC474794; 15.vi.1965, W.G. Mathewman, Voucher Code: CNC474795; 23.vi.1966, D.P. Pielou, Voucher Code: CNC474793; 4.vii.1966, D.P. Pielou, Voucher Code: CNC474796; 45.60057 -76.042647, 16.vi.1967, W. G. Matthewman, Voucher Code: CNCHYM00272; 22.vi.1966, D. P. Pielou, Voucher Code: CNCHYM00277; 4.vii.1967, W. G. Matthewman, Voucher Code: CNCHYM00279.

**Figure 12.**
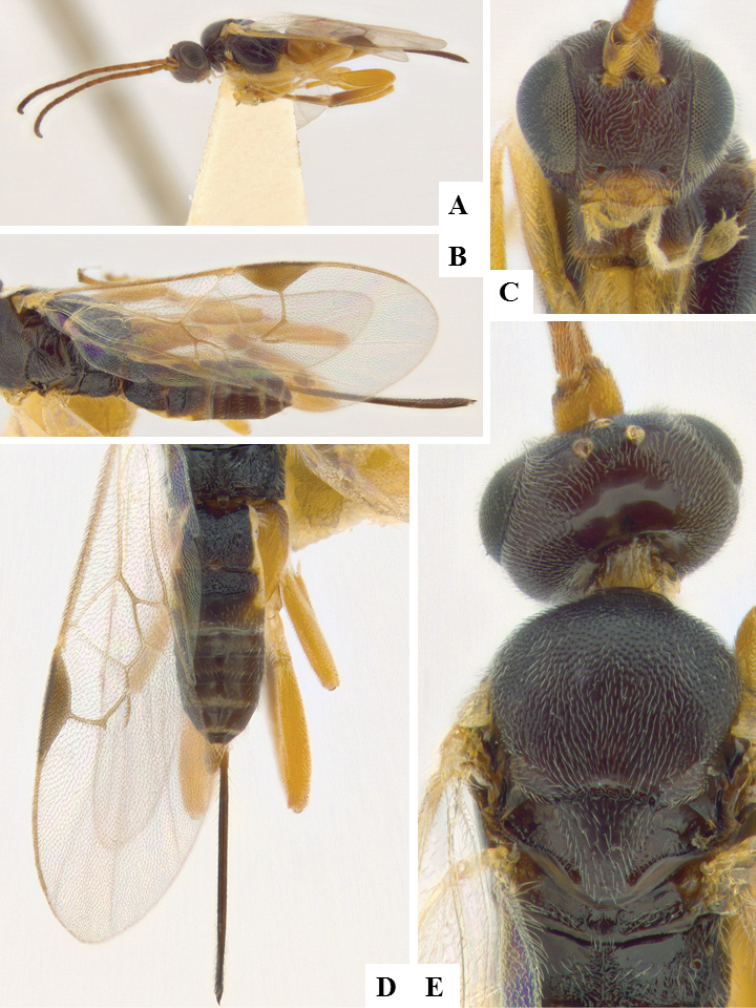
*Choeras consimilis*. **A** Habitus, lateral **B** Wings **C** Head, frontal **D** Metasoma dorsal **F** Head and mesosoma, dorsal.

#### Choeras parasitellae

Taxon classificationAnimaliaHymenopteraBraconidae

(Bouché, 1834)

##### Distribution.

NEA, PAL.

##### Notes.

This is the first record of this species for the Nearctic region.

##### Material examined.

Ontario, Ottawa, city garden, 45.3561 -75.7069, 16-26.vi.2007, H. Goulet, Voucher Code: CAM0908; 5.vi-2.vii.2008, H. Goulet, Voucher Code: CAM0906.

#### Choeras jft25

Taxon classificationAnimaliaHymenopteraBraconidae

##### Distribution.

NEA.

##### Notes.

This species corresponds in BOLD to BIN BOLD:AAD7965, with all specimens collected in southern Ontario.

##### Material examined.

Ontario, mixed forest, 45.235 -75.624, 19-29.vi.2007, A. Bennett, Voucher Code: CAM0565.

#### Choeras jft26

Taxon classificationAnimaliaHymenopteraBraconidae

##### Distribution.

NEA.

##### Notes.

This species corresponds in BOLD to BIN BOLD:AAD7964, with all specimens collected in Canada.

##### Material examined.

Ontario, mixed forest, 45.235 -75.624, 7-19.vi.2007, A. Bennett, Voucher Code: CAM0564.

#### Choeras jft34

Taxon classificationAnimaliaHymenopteraBraconidae

##### Distribution.

NEA.

##### Notes.

This species corresponds in BOLD to BIN BOLD:AAD7963, with all specimens collected in eastern Canada.

##### Material examined.

Ontario, mixed forest, 45.235 -75.624, 29.vi-16.vii.2007, A. Bennett, Voucher Code: CAM0566; Ottawa, city garden, 45.356 -75.707, 1-19.ix.2007, H. Goulet, Voucher Code: CAM0914; 1.ix.2007, H. Goulet, Voucher Code: CAM0084; 10.viii-1.ix.2007, H. Goulet, Voucher Code: CAM0913; 26.vi-13.vii.2007, H. Goulet, Voucher Code: CAM0916, CAM0918; Woodlawn, 45.375 -76.083, 6.viii.2008, L. Masner, Voucher Code: MIC000624, MIC000625.

#### Clarkinella canadensis

Taxon classificationAnimaliaHymenopteraBraconidae

Mason, 1981

Fig. 13

##### Distribution.

NEA.

##### Notes.

This species is only known from the type locality (Ottawa), where it has been rarely collected. The status of this species as a potential member of the Species Candidate Lists of COSEWIC was assessed by Fernandez Triana (2014).

##### Material examined.

Ontario, Ottawa, city garden, 45.356100 -75.707000, 30.vii.2007, H. Goulet, Voucher Code: CAM0263; 8.ix.2007, H. Goulet, Voucher Code: CAM0262; Ottawa, 45.356083 -75.706933, 28.vii.1959, S.M. Clark, 45.406631 -75.701407, 28.vii.1959, S.M. Clark, Voucher Code: CNC15769.

**Figure 13.**
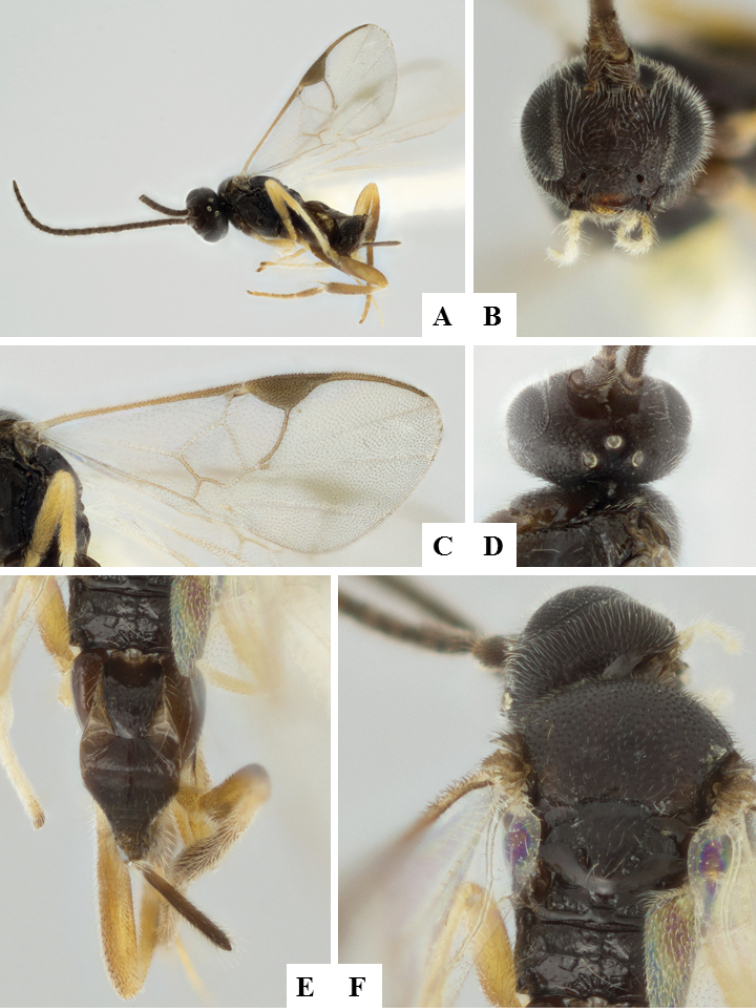
*Clarkinella canadensis*. **A** Habitus, lateral **B** Head, frontal **C** Wings **D** Head dorsal **E** Metasoma, dorsal **F** Head and mesosoma, dorsal.

#### Cotesia acronyctae

Taxon classificationAnimaliaHymenopteraBraconidae

(Riley, 1871)

Fig. 14

##### Distribution.

NEA.

##### Material examined.

Ontario, Ottawa, 45.406631 -75.701407, 4.x.1941, F.I.S., Voucher Code: CNC474798, CNC474799.

**Figure 14.**
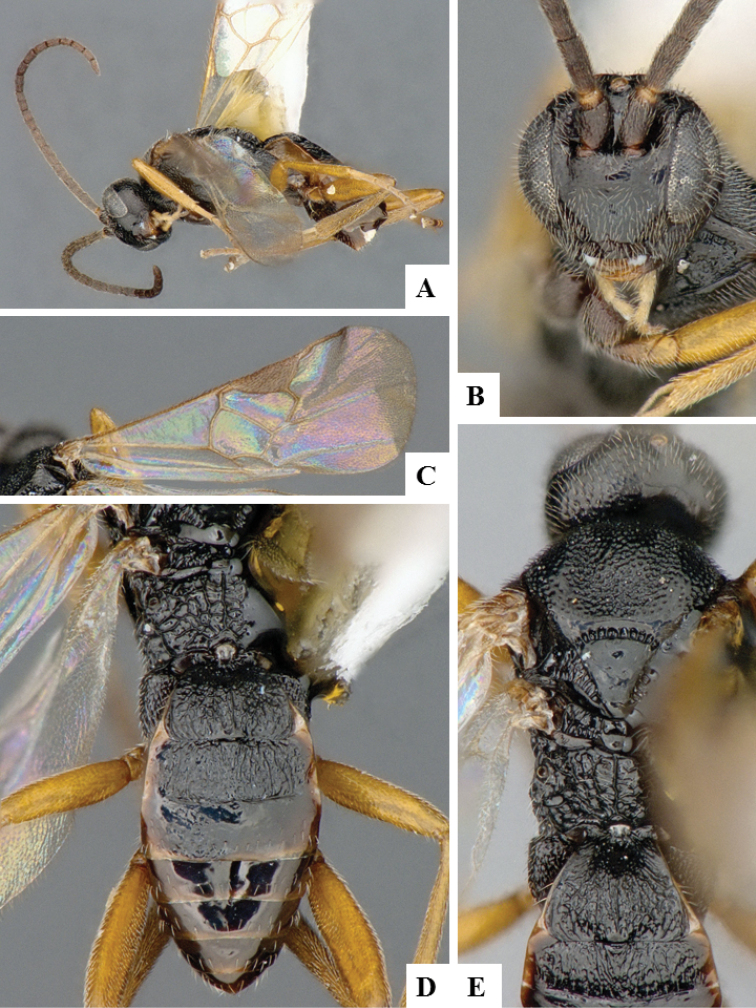
*Cotesia acronyctae*. **A** Habitus, lateral **B** Head, frontal **C** Wings **D** Metasoma, dorsal **E** Head and mesosoma, dorsal.

#### Cotesia atalantae

Taxon classificationAnimaliaHymenopteraBraconidae

(Packard, 1881)

Fig. 15

##### Distribution.

NEA.

##### Material examined.

Ontario, 6mi. W Richmond, 45.193269 -75.961153, 27.vii.1973, J.E.H. Martin, Voucher Code: CNC474807, CNC474808, CNC474809, CNC474810, CNC474811, CNC474812, CNC474813, CNC474814, CNC474815, CNC474816, CNC474817, CNC474818, CNC474819, CNC474820, CNC474821, CNC474822, CNC474823, CNC474824, CNC474825, CNC474826, CNC474827, CNC474828, CNC474829, CNC474830, CNC474831, CNC474832, CNC474833, CNC474834, CNC474835, CNC474836, CNC474837, CNC474838, CNC474839, CNC474840, CNC474841, CNC474842, CNC474843, CNC474844, CNC474845, CNC474846, CNC474847, CNC474848, CNC474849, CNC474850, CNC474851, CNC474852, CNC474853, CNC474854, CNC474855, CNC474856, CNC474857, CNC474858, CNC474859, CNC474860, CNC474861, CNC474862, CNC474863, CNC474864, CNC474865, CNC474866, CNC474867, CNC474868, CNC474869, CNC474870, CNC474871, CNC474872, CNC474873, CNC474874; 45.194311 -75.838992, 27.vii.1973, J. E. H. Martin, Voucher Code: CNCHYM00361; Ottawa, 45.356083 -75.706933, 31.vii.1939, Hobbs, G.A., Voucher Code: CNC280772; 45.406631 -75.701407, 31.vii.1939, G.A. Hobbs, Voucher Code: CNC474800, CNC474801, CNC474802, CNC474803, CNC474804, CNC474805, CNC474806.

**Figure 15.**
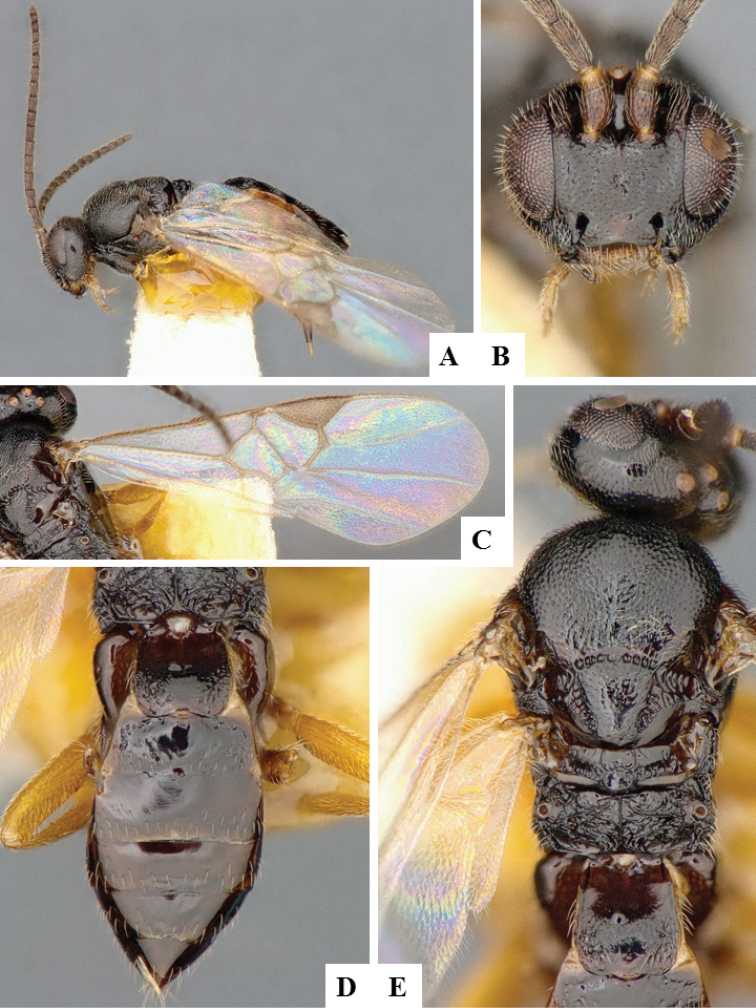
*Cotesia atalantae*. **A** Habitus, lateral **B** Head, frontal **C** Wings **D** Metasoma, dorsal **E** Head and mesosoma, dorsal.

#### Cotesia cerurae

Taxon classificationAnimaliaHymenopteraBraconidae

(Muesebeck, 1926)

##### Distribution.

NEA.

##### Material examined.

Ontario, Stittsville, 45.258796 -75.92113, 21.i.1946, Voucher Code: CNCHYM00373; 23.i.1946, Voucher Code: CNCHYM00376; 24.i.1945, Voucher Code: CNCHYM00375; 25.i.1946, Voucher Code: CNCHYM00374; 45.259037 -75.920958, 24.i.1946, F.I.S , Voucher Code: CNC280776.

#### Cotesia cingiliae

Taxon classificationAnimaliaHymenopteraBraconidae

(Muesebeck, 1931)

##### Distribution.

NEA.

##### Material examined.

Ontario, Merivale, 45.340734 -75.727462, 7.ix.1948, Voucher Code: CNCHYM00381; 45.340750 -75.727475, 7.ix.1948, F.I.S., Voucher Code: CNC280779.

#### Cotesia clisiocampae

Taxon classificationAnimaliaHymenopteraBraconidae

(Ashmead, 1903)

##### Distribution.

NEA.

##### Material examined.

Ontario, Mer Bleue, Ottawa, 45.393578 -75.512128, 27.v.1927-27.v.1997, G.S. Walley, Voucher Code: CNC280781; 45.393585 -75.512138, 17.v.1927, G.S. Walley, Voucher Code: CNC474883, CNC474884, CNC474885, CNC474886, CNC474887, CNC474888, CNC474889, CNC474890, CNC474891, CNC474892, CNC474893, CNC474894; 21.v.1927, G.S. Walley, Voucher Code: CNC474875, CNC474876, CNC474877, CNC474878, CNC474879, CNC474880, CNC474881, CNC474882; 27.v.1927, G.S. Walley, Voucher Code: CNC474895, CNC474896; 45.393593 -75.512138, 27.v.1937, G. S. Walley, Voucher Code: CNCHYM00386.

#### Cotesia congregata

Taxon classificationAnimaliaHymenopteraBraconidae

(Say, 1836)

##### Distribution.

NEA, NEO.

##### Material examined.

Ontario, Ottawa, city garden, 45.3561 -75.707, 1.ix.2007, H. Goulet, Voucher Code: CAM0268; Ottawa, 45.3825 -75.7137, 10.vii.1944, M. d`Aoust, Voucher Code: CNCHYM00396; 26.i.1942, F. I. Survey, Voucher Code: CNCHYM00394; 45.406631 -75.701407, 27.i.1942, F.I.S., Voucher Code: CNC474900; 30.i.1942, F.I.S., Voucher Code: CNC474899; 21.viii.1944, F.I.S., Voucher Code: CNC474897, CNC474898.

#### Cotesia crambi

Taxon classificationAnimaliaHymenopteraBraconidae

(Weed, 1887)

##### Distribution.

NEA.

##### Material examined.

Ontario, mixed forest near Manotick, Ottawa, 45.235 -75.624, 29.vi-16.vii.2007, A. Bennett, Voucher Code: CAM0542; Ottawa, city garden, 45.356 -75.707, 10.viii-1.ix.2007, H. Goulet, Voucher Code: CAM1034; 30.vii-10.viii.2007, H. Goulet, Voucher Code: CAM0893.

#### Cotesia depressa

Taxon classificationAnimaliaHymenopteraBraconidae

(Viereck, 1912)

##### Distribution.

NEA.

##### Material examined.

Quebec, Gatineau Park, 45.600555 -76.042647, 15.viii.1946, F.I.S., Voucher Code: CNCHYM00415.

#### Cotesia diacrisiae

Taxon classificationAnimaliaHymenopteraBraconidae

(Gahan, 1917)

##### Distribution.

NEA.

##### Material examined.

Quebec, Gatineau Park, 45.600556 -76.042647, 15.viii.1946, F.I.S., Voucher Code: CNC474901.

#### Cotesia fiskei

Taxon classificationAnimaliaHymenopteraBraconidae

(Viereck, 1910)

##### Distribution.

NEA.

##### Material examined.

Ontario, Stittsville, 45.258675 -75.921130, 1.viii.1951, F.I.S., Voucher Code: CNC474910, CNC474911, CNC474912, CNC474913, CNC474914, CNC474915, CNC474916, CNC474917, CNC474918, CNC474919, CNC474920, CNC474921; 31.vii.1951, F.I.S., Voucher Code: CNC474902, CNC474903, CNC474904, CNC474905, CNC474906, CNC474907, CNC474908, CNC474909; 45.258796 -75.92113, 31.vii.1951, Voucher Code: CNCHYM00466.

#### Cotesia flaviconchae

Taxon classificationAnimaliaHymenopteraBraconidae

(Riley, 1881)

##### Distribution.

NEA.

##### Material examined.

Ontario, Ottawa, 45.356083 -75.706933, 15.x.1959, Martin, J.E.H., Voucher Code: CNC280802; 45.3825 -75.7137, 16.x.1959, J. E. H. Martin, Voucher Code: CNCHYM00470; 19.x.1959, J. E. H. Martin, Voucher Code: CNCHYM00468; 23.x.1959, J. E. H. Martin, Voucher Code: CNCHYM00469; 45.406631 -75.701407, 15.x.1959, J.E.H. Martin, Voucher Code: CNC474922, CNC474923, CNC474924, CNC474925, CNC474926; 16.x.1959, J.E.H. Martin, Voucher Code: CNC474927, CNC474928, CNC474929, CNC474930, CNC474931, CNC474932, CNC474933, CNC474934, CNC474935, CNC474936, CNC474937, CNC474938, CNC474939, CNC474940, CNC474941, CNC474942, CNC474943, CNC474944, CNC474945; 19.x.1959, J.E.H. Martin, Voucher Code: CNC474946, CNC474947, CNC474948; 23.x.1959, J.E.H. Martin, Voucher Code: CNC474949, CNC474950; 45.406633 -75.701408, x.1959, J.E.H. Martin, Voucher Code: CNC474951.

#### Cotesia glomerata

Taxon classificationAnimaliaHymenopteraBraconidae

(Linnaeus, 1758)

##### Distribution.

Cosmopolitan.

##### Material examined.

Ontario, Long Swamp, Ottawa, 45.3825 -75.7137, 7.ix.1943, E. G. lester, Voucher Code: CNCHYM00489; 45.419163 -75.709648, 7.ix.1963, Lester, E.G., Voucher Code: CNC280801.

#### Cotesia halisidotae

Taxon classificationAnimaliaHymenopteraBraconidae

(Muesebeck, 1931)

##### Distribution.

NEA.

##### Material examined.

Ontario, Ottawa, 45.406633 -75.701408, 1908, Voucher Code: CNC474952; 30.v.1908, Voucher Code: CNC474953.

#### Cotesia hemileucae

Taxon classificationAnimaliaHymenopteraBraconidae

(Riley, 1881)

##### Distribution.

NEA.

##### Material examined.

Ontario, Carlsbad Springs, 45.369133 -75.456226, 10.viii.1946, F.I.S., Voucher Code: CNC474958, CNC474959, CNC474960; 6.viii.1946, F.I.S., Voucher Code: CNC474954, CNC474955, CNC474956, CNC474957; 45.369194 -75.456140, 6.viii.1946, F.I.S., Voucher Code: CNC280807; 45.369254 -75.456097, 6.viii.1946, Voucher Code: CNCHYM00510.

#### Cotesia hyphantriae

Taxon classificationAnimaliaHymenopteraBraconidae

(Riley, 1887)

##### Distribution.

NEA, PAL.

##### Material examined.

Ontario, Leitrum, 45.329824 -75.598377, 10.viii.1946, F.I.S., Voucher Code: CNC474968; 12.viii.1946, F.I.S., Voucher Code: CNC474964, CNC474965; 15.viii.1946, F.I.S., Voucher Code: CNC474966; 19.viii.1946, F.I.S., Voucher Code: CNC474967; 24.viii.1946, F.I.S., Voucher Code: CNC474962, CNC474963; Ottawa, 45.406631 -75.701407, 10.viii.1950, D.W. Peters, Voucher Code: CNC474961.

#### Cotesia laeviceps

Taxon classificationAnimaliaHymenopteraBraconidae

(Ashmead, 1890)

##### Distribution.

NEA.

##### Material examined.

Ontario, Ottawa, 45.3825 -75.7137, 25.v.1943, G. S. Walley, Voucher Code: CNCHYM00557; 45.406631 -75.701407, 25.v.1943, G.S. Walley, Voucher Code: CNC474969, CNC474970, CNC474971, CNC474972, CNC474973, CNC474974, CNC474975, CNC474976, CNC474977, CNC474978, CNC474979, CNC474980, CNC474981, CNC474982, CNC474983, CNC474984, CNC474985, CNC474986, CNC474987, CNC474988, CNC474989, CNC474990, CNC474991, CNC474992, CNC474993, CNC474994, CNC474995, CNC474996, CNC474997, CNC474998, CNC474999, CNC475000, CNC475001, CNC475002, CNC475003, CNC475004, CNC475005, CNC475006, CNC475007, CNC475008, CNC475009, CNC475010, CNC475011, CNC475012.

#### Cotesia melanoscela

Taxon classificationAnimaliaHymenopteraBraconidae

(Ratzeburg, 1844)

Fig. 16

##### Distribution.

NEA. OTL, PAL.

##### Notes.

This is mostly a Holarctic species, with some records from the Oriental region due to being a parasitoid of the Gypsy Moth, *Lymantria dispar* (Linnaeus, 1758).

##### Material examined.

Ontario, Kilbirnie, 45.247853 -75.724400, 26.iii.1981, Voucher Code: CNC475048; 31.iii.1981, Voucher Code: CNC475046, CNC475047.

**Figure 16.**
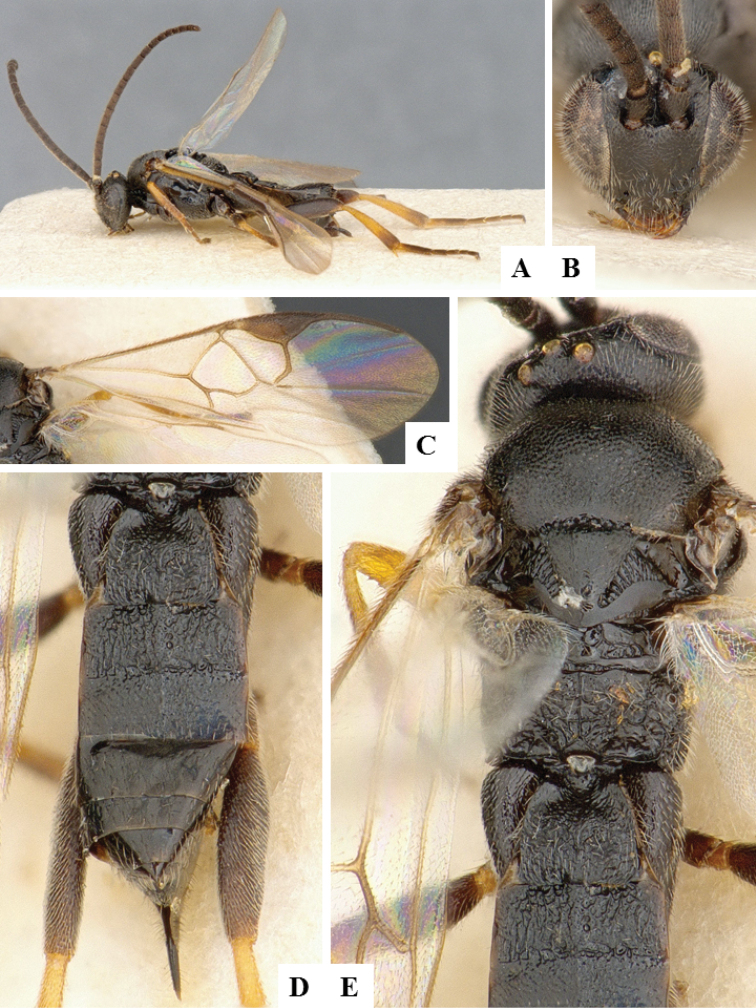
*Cotesia melanoscela*. **A** Habitus, lateral **B** Head, frontal **C** Wings **D** Metasoma, dorsal **E** Head and mesosoma, dorsal.

#### Cotesia nemoriae

Taxon classificationAnimaliaHymenopteraBraconidae

(Ashmead, 1898)

##### Distribution.

NEA.

##### Material examined.

Ontario, Ottawa, 45.406631 -75.701407, 14.vi.1943, Voucher Code: CNC475049; Quebec, Old Chelsea, 45.503548 -75.797963, 25.v.1942, Voucher Code: CNCHYM00605; 45.541317 -75.867939, 25.v.1942, F.I.S., Voucher Code: CNC475050.

#### Cotesia parastichtidis

Taxon classificationAnimaliaHymenopteraBraconidae

(Muesebeck, 1921)

##### Distribution.

NEA.

##### Material examined.

Ontario, Ottawa, city garden, 45.356 -75.707, H. Goulet, Voucher Code: CAM1038; 45.356083 -75.706933, malaise trap, 8-16.vi.2007, Goulet, H. , Voucher Code: CNC280831; Ottawa, 45.3825 -75.7137, 1.iv.1941, Voucher Code: CNCHYM00636; 45.406633 -75.701408, 1.vi.1941, F.I.S., Voucher Code: CNC475054, CNC475055; 31.v.1941, F.I.S., Voucher Code: CNC475052, CNC475053.

#### Cotesia phobetri

Taxon classificationAnimaliaHymenopteraBraconidae

(Rohwer, 1915)

##### Distribution.

NEA.

##### Material examined.

Ontario, Ottawa, 45.406631 -75.701407, 23.ii.1948, F.I.S., Voucher Code: CNC475056; 3.ix.1947, F.I.S., Voucher Code: CNC475059, CNC475060.

#### Cotesia schizurae

Taxon classificationAnimaliaHymenopteraBraconidae

Ashmead, 1898

##### Distribution.

NEA.

##### Material examined.

Ontario, Ottawa, 45.356083 -75.706933, 6.viii.1959, Downes, A.J., Voucher Code: CNC280849; 45.3825 -75.7137, 6.viii.1959, A. J. Downes, Voucher Code: CNCHYM00699; 45.406631 -75.701407, 6.viii.1959, A.J. Downes, Voucher Code: CNC475061, CNC475062, CNC475063, CNC475064, CNC475065.

#### Cotesia xylina

Taxon classificationAnimaliaHymenopteraBraconidae

(Say, 1836)

##### Distribution.

NEA.

##### Material examined.

Ontario, Ottawa, city garden, 45.356 -75.707, 23.vii.2009, L. Masner, Voucher Code: CNCH0464; Quebec, Gatineau Park, 45.60057 -76.042647, 24.v.2007, L. Masner, Voucher Code: HYM00001130.

#### Cotesia jft02

Taxon classificationAnimaliaHymenopteraBraconidae

##### Distribution.

NEA.

##### Notes.

This species corresponds in BOLD to BIN BOLD:ABY4229, with all specimens collected in North America.

##### Material examined.

Ontario, mixed forest, 45.235 -75.624, 7-19.vi.2007, A. Bennett, Voucher Code: CAM0538; Ottawa, city garden, 45.356 -75.707, 10.viii-1.ix.2007, H. Goulet, Voucher Code: CAM1033; 20-29.ix.2008, H. Goulet, Voucher Code: CAM1036; 26.vi-13.vii.2007, H. Goulet, Voucher Code: HYM00000999.

#### Cotesia jft03

Taxon classificationAnimaliaHymenopteraBraconidae

##### Distribution.

NEA.

##### Notes.

This undescribed species, so far only recorded from Canada seems to be an important parasitoid (at least locally) of the Diamondback Moth, *Plutella xylostella* (Linnaeus, 1758). This species corresponds in BOLD to BIN BOLD:AAA9378.

##### Material examined.

Ontario, North Gower to Smith Falls, 1 km N of Rd 6 & Montague Bdy Rd, 45.033 -75.9, 15.vi.2004, Bennett & Barnes, Voucher Code: HYM00001204, HYM00001205, HYM00001206; Ottawa, CNC breeding program, Ottawa, 45.3825 -75.7137, 31.iii.2008, Jose L. Fernandez Triana, Voucher Code: CPWH-0033, CPWH-0034, CPWH-0035, CPWH-0036, CPWH-0037, CPWH-0038, CPWH-0039, CPWH-0040, CPWH-0041, CPWH-0042, CPWH-0043, CPWH-0044, CPWH-0045, CPWH-0046, CPWH-0047, CPWH-0048, CPWH-0049, CPWH-0050, CPWH-0051, CPWH-0052; Ottawa, city garden, 45.3561 -75.7069, 30.vii-10.viii.2007, H. Goulet, Voucher Code: CNCH0510; 45.356100 -75.706900, 30.vii-10.viii.2007, H. Goulet, Voucher Code: CNCH0509, CNCH0511.

#### Cotesia jft07

Taxon classificationAnimaliaHymenopteraBraconidae

##### Distribution.

NEA, PAL.

##### Notes.

This species seems to be related to the European *Cotesia salebrosa* (Marshall, 1885), or perhaps it is that same species (in which case it would be the first record of the species for the Nearctic). Pending further study of more material from other localities, we prefer to keep it as an undescribed for the time being. This species corresponds in BOLD to BIN BOLD:ABZ3751.

##### Material examined.

Ontario, North Gower to Smith Falls, 1 km N of Rd 6 & Montague Bdy Rd, 45.033 -75.9, 15.vi.2004, Bennett & Barnes, Voucher Code: HYM00001132, HYM00001133, HYM00001135, HYM00001136, HYM00001137, HYM00001138, HYM00001139, HYM00001140, HYM00001141, HYM00001142, HYM00001143, HYM00001144, HYM00001145; Ottawa, city garden, 45.356 -75.707, 19.ix-8.xi.2007, H. Goulet, Voucher Code: HYM00000997; Quebec, Chelsea, near old visitor center, 45.541075 -75.867938, 11.xi.2008, J. Fernandez, Voucher Code: CAM0569, CAM0570.

#### Cotesia jft09

Taxon classificationAnimaliaHymenopteraBraconidae

##### Distribution.

NEA, PAL.

##### Notes.

This species seems to be related to a complex of species, from both Europe and North America. For the time being is left as an undescribed species, until more studies of the Holarctic fauna are carried out. This species corresponds in BOLD to BIN BOLD:AAA6099.

##### Material examined.

Ontario, 1.6 km SW of Manion Corners, Carleton Co., 45.25 -76.083, 1.iv.2004, R. Layberry, Voucher Code: CNCH0950; 25.iii.2004, R. Layberry, Voucher Code: CNCH0951.

#### Cotesia jft20

Taxon classificationAnimaliaHymenopteraBraconidae

##### Distribution.

NEA.

##### Notes.

This species corresponds in BOLD to BIN BOLD:AAD6124, with all specimens collected in southern Ontario.

##### Material examined.

Ontario, Ottawa, city garden, 45.3561 -75.7069, 8-16.vi.2007, H. Goulet, Voucher Code: CAM1037.

#### Cotesia jft22

Taxon classificationAnimaliaHymenopteraBraconidae

##### Distribution.

NEA.

##### Notes.

This species corresponds in BOLD to BIN BOLD:AAF0583, with all specimens collected in North America.

##### Material examined.

Ontario, Ottawa, city garden, 45.3561 -75.7069, 8-16.vi.2007, H. Goulet, Voucher Code: CAM1039.

#### Cotesia jft42

Taxon classificationAnimaliaHymenopteraBraconidae

##### Distribution.

NEA.

##### Notes.

This species corresponds in BOLD to BIN BOLD:AAR9059, with only a single specimen known from Ottawa.

##### Material examined.

Ontario, mixed forest, 45.235 -75.624, 29.vi-16.vii.2007, A. Bennett, Voucher Code: CAM0540.

#### Cotesia jft44

Taxon classificationAnimaliaHymenopteraBraconidae

##### Distribution.

NEA.

##### Notes.

This species corresponds in BOLD to BIN BOLD:AAR9061, with only a single specimen known from Ottawa.

##### Material examined.

Ontario, mixed forest, 45.235 -75.624, 29.vi-16.vii.2007, A. Bennett, Voucher Code: CAM0541.

#### Cotesia jft53

Taxon classificationAnimaliaHymenopteraBraconidae

##### Distribution.

NEA.

##### Notes.

This species corresponds in BOLD to BIN BOLD:ABX6366, with all specimens collected in North America.

##### Material examined.

Ontario, 5 km NW of Almonte, Hwy 49, Burnt Land, Alvar Prov. Park, Almonte, 45.255 -76.14, 29.v.2008, Goulet & Fernandez, Voucher Code: CAM0332, CAM0333.

#### Cotesia

Taxon classificationAnimaliaHymenopteraBraconidae

Whitfield26

##### Distribution.

NEA.

##### Notes.

This species corresponds in BOLD to BIN BOLD:AAH2149, with all specimens collected in southern Ontario.

##### Material examined.

Ontario, mixed forest, 45.234700 -75.624000, 29.vi-16.vii.2007, A. Bennett, Voucher Code: CAM0539; Ottawa, city garden, 45.356100 -75.706900, 16-26.vi.2007, H. Goulet, Voucher Code: CAM0897.

#### Diolcogaster auripes

Taxon classificationAnimaliaHymenopteraBraconidae

(Provancher, 1886)

##### Distribution.

NEA.

##### Material examined.

Ontario, Aylmer West, 45.4 -75.85, 16.viii.1972, Voucher Code: CNCHYM00790; Innisville, 45.055468 -76.250497, 14.viii.1963, W.R.M. Mason, Voucher Code: CNC475090, CNC475091; mixed forest, 45.2347 -75.624, 29.vi-16.vii.2007, A. Bennett, Voucher Code: CAM0546; Ottawa , 45.419164 -75.709650, 11.vii.1951, Guppy, J.C., Voucher Code: CNC280871; Ottawa, 45.3825 -75.7137, 11.vii.1957, J. C. Guppy, Voucher Code: CNCHYM00787; 45.406631 -75.701407, 28.vi.1947, W.R.M. Mason, Voucher Code: CNC475087; Stittsville, 45.258675 -75.921130, 5.vi.1963, W.R.M. Mason, Voucher Code: CNC475089; 45.258796 -75.92113, 14.vi.1969, W. R. M. Mason, Voucher Code: CNCHYM00789; Thurso, 45.596990 -75.243571, 20.viii.1958, L.A. Kelton, Voucher Code: CNC475088; Quebec, Hull, 45.428309 -75.713353, 13.vi.1965, Voucher Code: CNCHYM00788; 45.428550 -75.714554, Malaise trap, vi.1965, Voucher Code: CNC475092; Old Chelsea, 45.500055 -75.814616, 26.v.1964, J.R. Vockeroth, Voucher Code: CNC475093; Thurso, 45.59729 -75.243828, 20.viii.1985, L. A. Kelton, Voucher Code: CNCHYM00786.

#### Diolcogaster claritibia

Taxon classificationAnimaliaHymenopteraBraconidae

(Papp, 1959)

Fig. 17

##### Distribution.

NEA, PAL.

##### Material examined.

Ontario, CEF, DBM Field Cage Trials, 45.389959 -75.711949, 23.vi.2010, P. Mason, S. Girardoz, Voucher Code: CNCHYM01690, CNCHYM01691, CNCHYM01692, CNCHYM01693, CNCHYM01694, CNCHYM01695.

**Figure 17.**
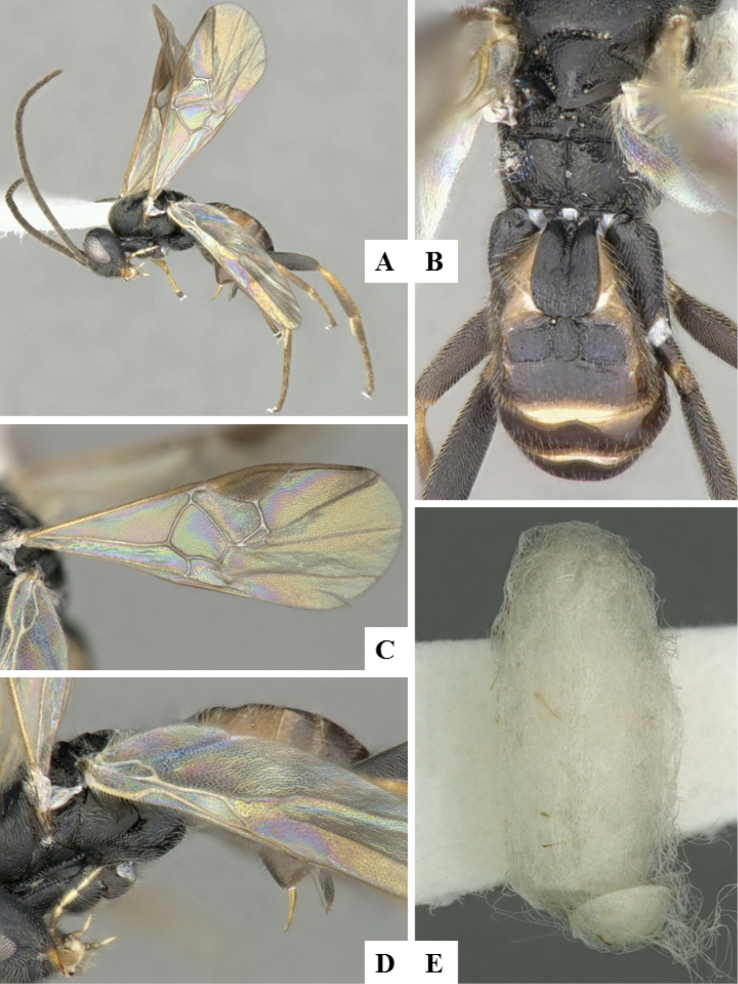
*Diolcogaster claritibia*. **A** Habitus, lateral **B** Mesosoma (partially) and metasoma dorsal **C** Wings **D** Mesosoma (partially) and metasoma, lateral **E** Cocoon.

#### Diolcogaster facetosa

Taxon classificationAnimaliaHymenopteraBraconidae

(Weed, 1888)

##### Distribution.

NEA.

##### Material examined.

Ontario, Innisville, 45.054942 -76.250619, 29.viii.1963, W. R. M. Mason, Voucher Code: CNCHYM00822; 45.055468 -76.250497, 28.vii.1963, W.R.M. Mason, Voucher Code: CNC475104; 31.v.1963, W.R.M. Mason, Voucher Code: CNC475103; Ottawa, 45.406631 -75.701407, 15.vii.1987, W.R.M. Mason, Voucher Code: CNC475099; 20.vii.1955, W.R.M. Mason, Voucher Code: CNC475105, CNC475106, CNC475107; 26.vii.1955, W.R.M. Mason, Voucher Code: CNC475108, CNC475109, CNC475110, CNC475111, CNC475112, CNC475113, CNC475114, CNC475115, CNC475116; Stittsville, 45.258675 -75.921130, 22.vii.1963, W.R.M. Mason, Voucher Code: CNC475102; 27.v.1963, W.R.M. Mason, Voucher Code: CNC475100; 5.vi.1963, W.R.M. Mason, Voucher Code: CNC475101; Woodlawn, 45.375 -76.083, 6.viii.2008, L. Masner, Voucher Code: MIC000622; Quebec, Aylmer West, 45.395345 -75.844876, Malaise trap, 1.vii.1973, Voucher Code: CNC475118; 6-13.vii.1973, Voucher Code: CNC475117; Aylmer, 45.395345 -75.844876, 10.vi.1924, C.H. Curran, Voucher Code: CNC475095; 27.vii.1924, C.H. Curran, Voucher Code: CNC475097; 29.v.1924, C.H. Curran, Voucher Code: CNC475094; 8.vi.1924, C.H. Curran, Voucher Code: CNC475096; Old Chelsea, 45.541315 -75.867938, 13.v.1965, G.S. Walley, Voucher Code: CNC475119; Wakefield, 45.631572 -75.924033, 16.iv.1938, F.I.S., Voucher Code: CNC475120.

#### Diolcogaster jft30

Taxon classificationAnimaliaHymenopteraBraconidae

##### Distribution.

NEA.

##### Notes.

This species corresponds in BOLD to BIN BOLD:AAB0185, with all specimens collected in North America.

##### Material examined.

Ontario, 5 km NW of Almonte, Hwy 49, Burnt Land, Alvar Prov. Park, Almonte, 45.2549 -76.14, 29.v.2008, Goulet & Fernandez, Voucher Code: CAM0366, CAM0367.

#### Diolcogaster jft35

Taxon classificationAnimaliaHymenopteraBraconidae

##### Distribution.

NEA.

##### Notes.

This species corresponds in BOLD to BIN BOLD:AAX9631, with only a single specimen known from Ottawa.

##### Material examined.

Ontario, mixed forest, 45.2347 -75.624, 19-29.vi.2007, A. Bennett, Voucher Code: CAM0545.

#### Diolcogaster jft38

Taxon classificationAnimaliaHymenopteraBraconidae

##### Distribution.

NEA.

##### Notes.

This species corresponds in BOLD to BIN BOLD:AAI6267, with only a single specimen known from Ottawa.

##### Material examined.

Quebec, Gatineau Park, 45.60057 -76.042647, 24.v.2007, L. Masner, Voucher Code: HYM00001284.

#### Diolcogaster jft41

Taxon classificationAnimaliaHymenopteraBraconidae

##### Distribution.

NEA.

##### Notes.

This species corresponds in BOLD to BIN BOLD:AAB0192, with all specimens collected in Canada.

##### Material examined.

Ontario, mixed forest, 45.2347 -75.624, 29.vi-16.vii.2007, A. Bennett, Voucher Code: CAM0547, CAM0548, CAM0549.

#### Distatrix carolinae

Taxon classificationAnimaliaHymenopteraBraconidae

Fernandez-Triana, 2010

Fig. 18

##### Distribution.

NEA.

##### Notes.

The status of this species as a potential member of the Species Candidate Lists of COSEWIC was assessed by Fernandez Triana (2014).

##### Material examined.

Quebec, Summit King Mt. Old Chelsea, 45.500147 -75.814703, 26.vi.1977, M. Sandborne, Voucher Code: CNC23940.

**Figure 18.**
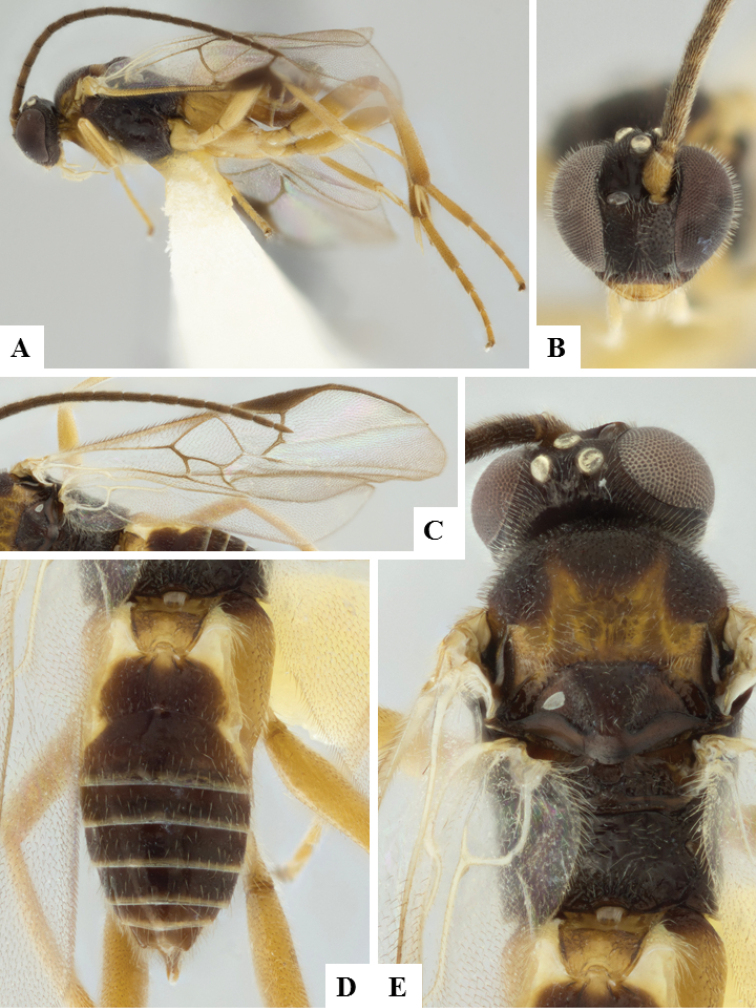
*Distatrix carolinae*. **A** Habitus, lateral **B** Head, frontal **C** Wings **D** Metasoma, dorsal **E** Head and mesosoma, dorsal.

#### Dolichogenidea absona

Taxon classificationAnimaliaHymenopteraBraconidae

(Muesebeck, 1965)

Fig. 19

##### Distribution.

NEA.

##### Material examined.

Ontario, Mer Bleue, 45.393593 -75.512138, 26.ii.1961, Freeman & Lewis, Voucher Code: MIC000241, MIC000242.

**Figure 19.**
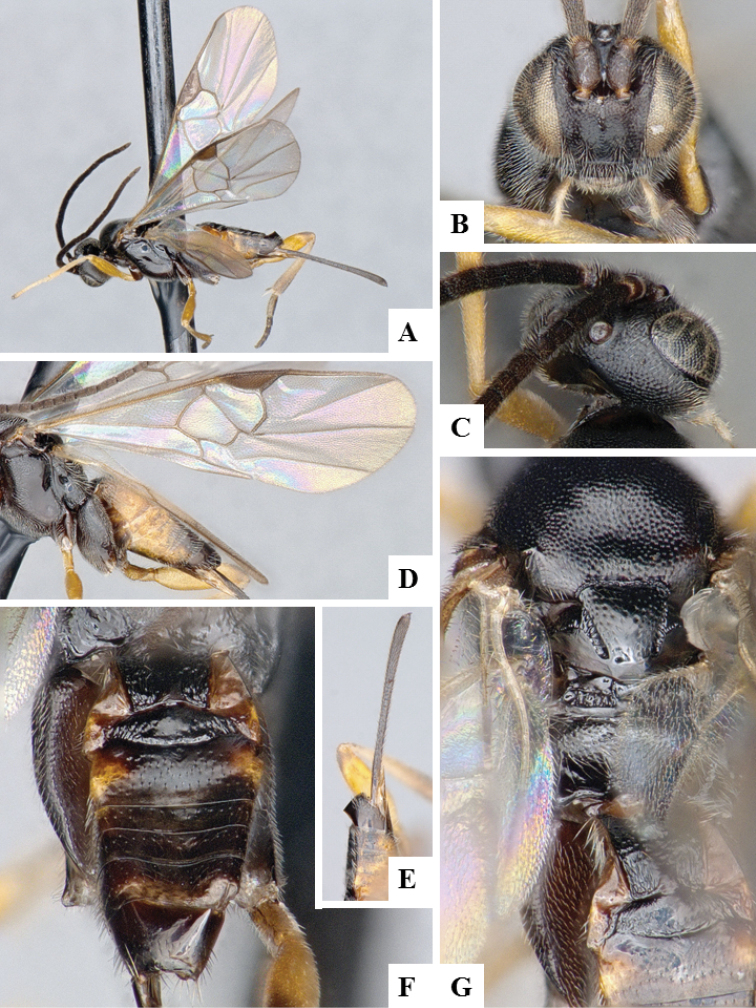
*Dolichogenidea absona*. **A** Habitus, lateral **B** Head, frontal **C** Head, frontal **D** Wings **E** Ovipositor sheaths **F** Metasoma, dorsal **G** Mesosoma, dorsal.

#### Dolichogenidea cacoeciae

Taxon classificationAnimaliaHymenopteraBraconidae

(Riley, 1881)

Fig. 20

##### Distribution.

NEA.

##### Material examined.

Ontario, Merivale, 45.325948 -75.719082, 22.vi.1935, E.G. Lester, Voucher Code: CNC475188, CNC475189, CNC475190, CNC475191; Ottawa, 45.356083 -75.706933, 9.iv.1953, C.D. Miller, Voucher Code: CNC280909; 45.3825 -75.7137, 14.xi.1969, A. Sauve, Voucher Code: CNCHYM00994; 45.406631 -75.701407, 12.v.1969, A. Sauve, Voucher Code: CNC475161, CNC475162; 13.xi.1969, A. Sauve, Voucher Code: CNC475142, CNC475143, CNC475144, CNC475145, CNC475146, CNC475147, CNC475148, CNC475149, CNC475150; 14.xi.1969, A. Sauve, Voucher Code: CNC475130, CNC475131, CNC475132, CNC475133, CNC475134, CNC475135, CNC475136, CNC475137, CNC475138, CNC475139, CNC475140, CNC475141; 19.ii.1969, A. Sauve, Voucher Code: CNC475154, CNC475155, CNC475156, CNC475157, CNC475158, CNC475159, CNC475160; 20.ii.1969, A. Sauve, Voucher Code: CNC475151, CNC475152, CNC475153; 21.ii.1969, A. Sauve, Voucher Code: CNC475163; 5.v.1944, G.S. Walley, Voucher Code: CNC475193, CNC475194, CNC475195; 6.v.1944, G.S. Walley, Voucher Code: CNC475196, CNC475197, CNC475198, CNC475199, CNC475200, CNC475201; 9.iv.1953, C.D. Miller, Voucher Code: CNC475121, CNC475122, CNC475123, CNC475124, CNC475125, CNC475126, CNC475127, CNC475128, CNC475129; Quebec, Hull, 45.428550 -75.714554, 2.v.1965, G.S.Walley, Voucher Code: CNC475192.

**Figure 20.**
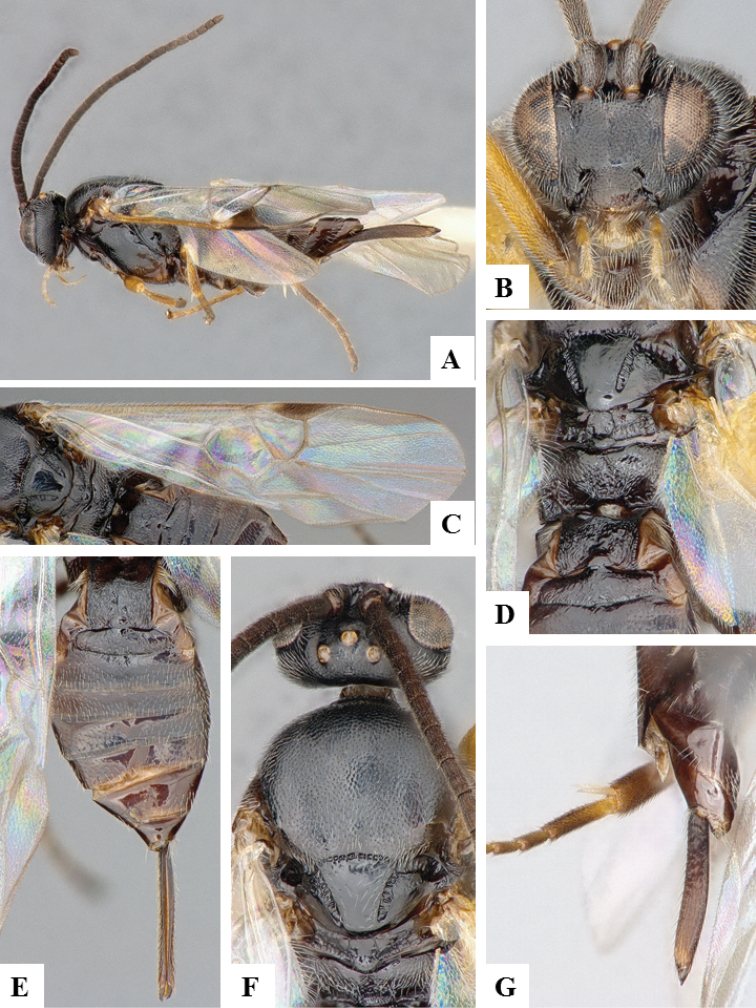
*Dolichogenidea cacoeciae*. **A** Habitus, lateral **B** Head, frontal **C** Wings **D** Mesosoma and metasoma (partially), dorsal **E** Metasoma, dorsal **F** Head and mesosoma, dorsal **G** Ovipositor sheaths.

#### Dolichogenidea paralechiae

Taxon classificationAnimaliaHymenopteraBraconidae

(Muesebeck, 1932)

Fig. 21

##### Distribution.

NEA.

##### Material examined.

Ontario, Kemptville, 45.016409 -75.646449, 9.v.1952, Voucher Code: CNC475203, CNC475204; Stittsville, 45.258675 -75.921130, 23.vi.1951, F.I.S., Voucher Code: CNC475202.

**Figure 21.**
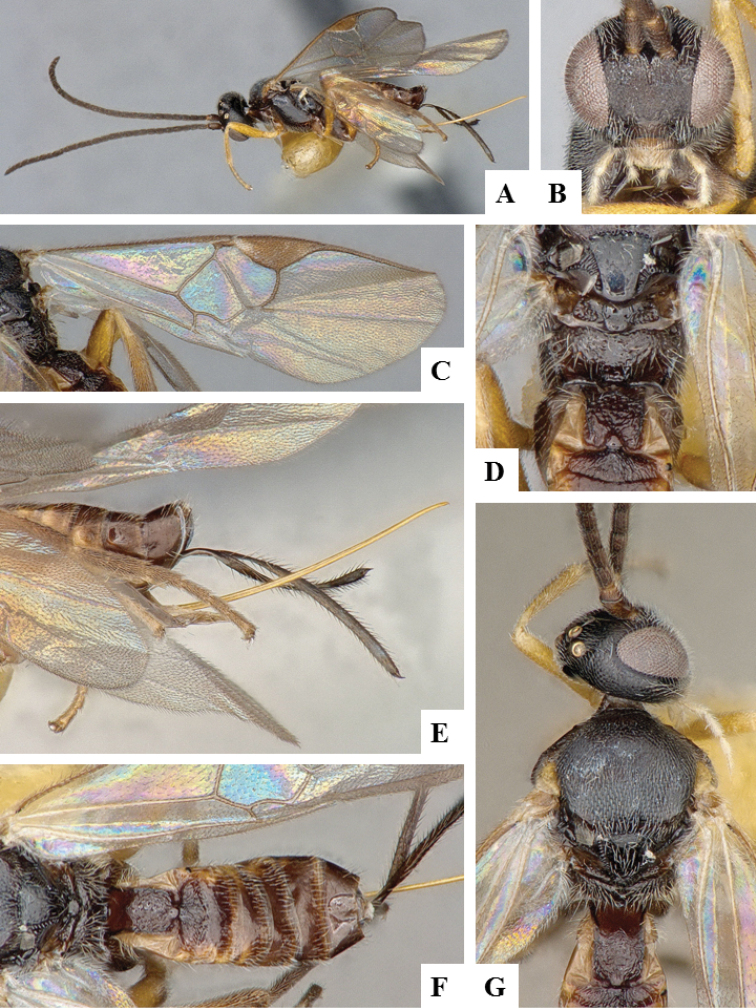
*Dolichogenidea paralechiae*. **A** Habitus, lateral **B** Head, frontal **C** Wings **D** Mesosoma and metasoma (partially), dorsal **E** Metasoma, lateral **F** Metasoma, dorsal **E** Head and mesosoma, dorsal.

#### Dolichogenidea renaulti

Taxon classificationAnimaliaHymenopteraBraconidae

(Mason, 1974)

Fig. 22

##### Distribution.

NEA.

##### Material examined.

Ontario, South March, 45.337746 -75.957675, 1.vi.1964, C.D. Miller, Voucher Code: CNC475214; 10.vi.1963, C.D. Miller, Voucher Code: CNC280950; 10.vi.1964, C.D. Miller, Voucher Code: CNC475211; 16.v.1963, C.D. Miller, Voucher Code: CNC475205, CNC475206, CNC475207; 16.vi.1964, C.D. Miller, Voucher Code: CNC475212; 17.v.1963, C.D. Miller, Voucher Code: CNC475208, CNC475209; 19.vi.1961, C.D. Miller, Voucher Code: CNC475216; 23.vi.1961, C.D. Miller, Voucher Code: CNC475215; 24.vi.1963, C.D. Miller, Voucher Code: CNC475210; 28.vi.1961, C.D. Miller, Voucher Code: CNC475217, CNC475218; 4.vi.1964, C.D. Miller, Voucher Code: CNC475213; 45.348507 -75.923123, 29.v.1961, C. Miller, Voucher Code: MIC000262; 45.348508 -75.923125, 16.v.1963, C. Miller, Voucher Code: MIC000256; 27.v.1965, C. Miller, Voucher Code: MIC000257.

**Figure 22.**
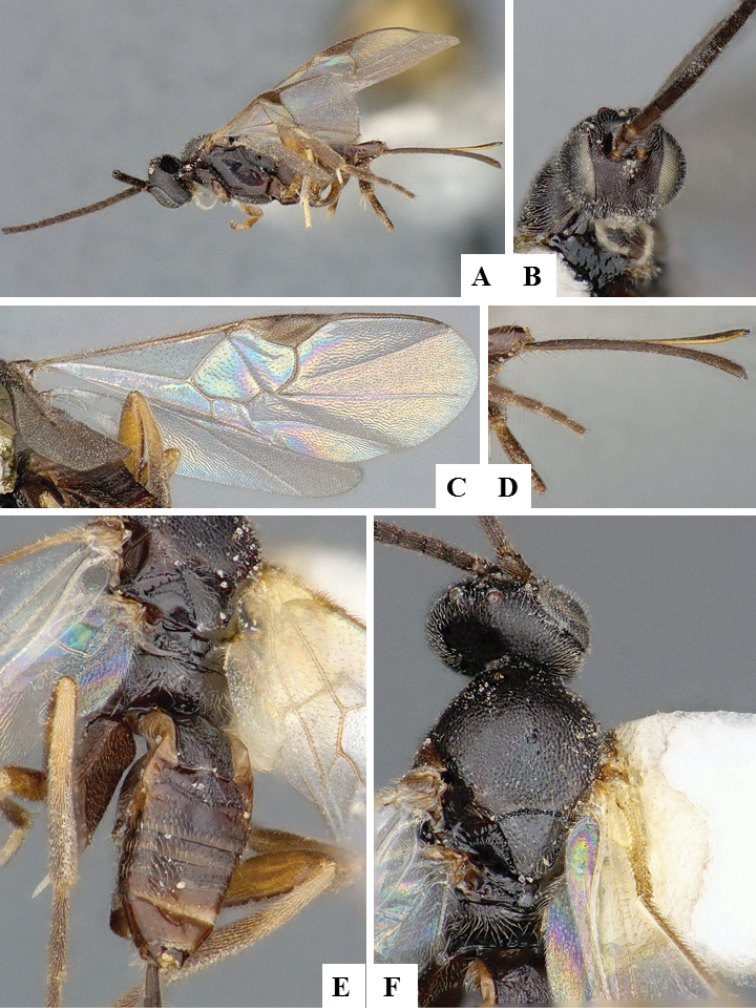
*Dolichogenidea renaulti*. **A** Habitus, lateral **B** Head, frontal **C** Wings **D** Ovipositor sheaths **E** Mesosoma (partially) and metasoma, dorsal **F** Head and mesosoma, dorsal.

#### Dolichogenidea solenobiae

Taxon classificationAnimaliaHymenopteraBraconidae

(Walley, 1935)

Fig. 23

##### Distribution.

NEA.

##### Material examined.

Ontario, Ottawa, 45.3825 -75.7137, 31.iii.1945, G. S. Walley, Voucher Code: CNCHYM01141.

**Figure 23.**
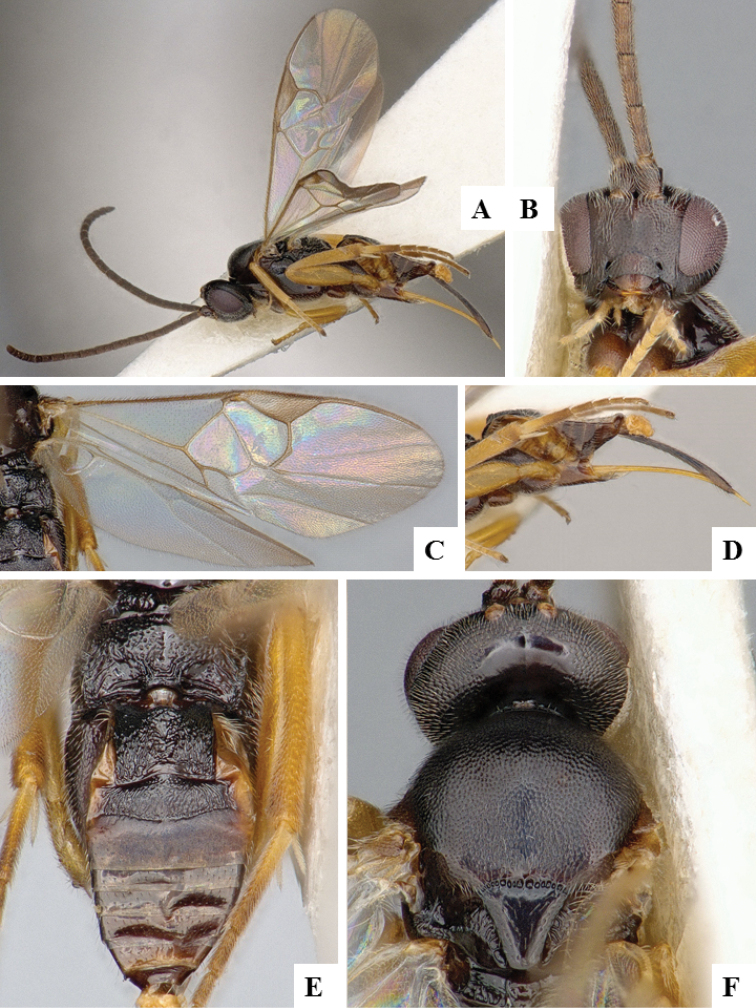
*Dolichogenidea solenobiae*. **A** Habitus, lateral **B** Head, frontal **C** Wings **D** Ovipositor sheaths and ovipositor **E** Metasoma, dorsal **F** Head and mesosoma, dorsal.

#### Dolichogenidea thujae

Taxon classificationAnimaliaHymenopteraBraconidae

(Muesebeck, 1935)

Fig. 24

##### Distribution.

NEA.

##### Material examined.

Ontario, Fallowfield, 45.267410 -75.829929, 18.vii.1960, C.D. Miller, Voucher Code: CNC475222; 19.vii.1960, C.D. Miller, Voucher Code: CNCHYM01157; 45.267531 -75.829886, 28.vii.1960, C.D. Miller, Voucher Code: CNC280957; Lanark, 45.018287 -76.365297, 27.vi.1951, Voucher Code: CNCHYM01159; 45.018354 -76.365257, 26.vi.1951, F.I.S., Voucher Code: CNC475224; 27.vi.1951, F.I.S., Voucher Code: CNC475223; Quebec, Aylmer, 45.395345 -75.844876, 14.vii.1960, C.D. Miller, Voucher Code: CNC475220; 15.vii.1960, C.D. Miller, Voucher Code: CNC475219; 18.vii.1960, C.D. Miller, Voucher Code: CNC475221; 45.4 -75.85, 19.vii.1960, Voucher Code: CNCHYM01158.

**Figure 24.**
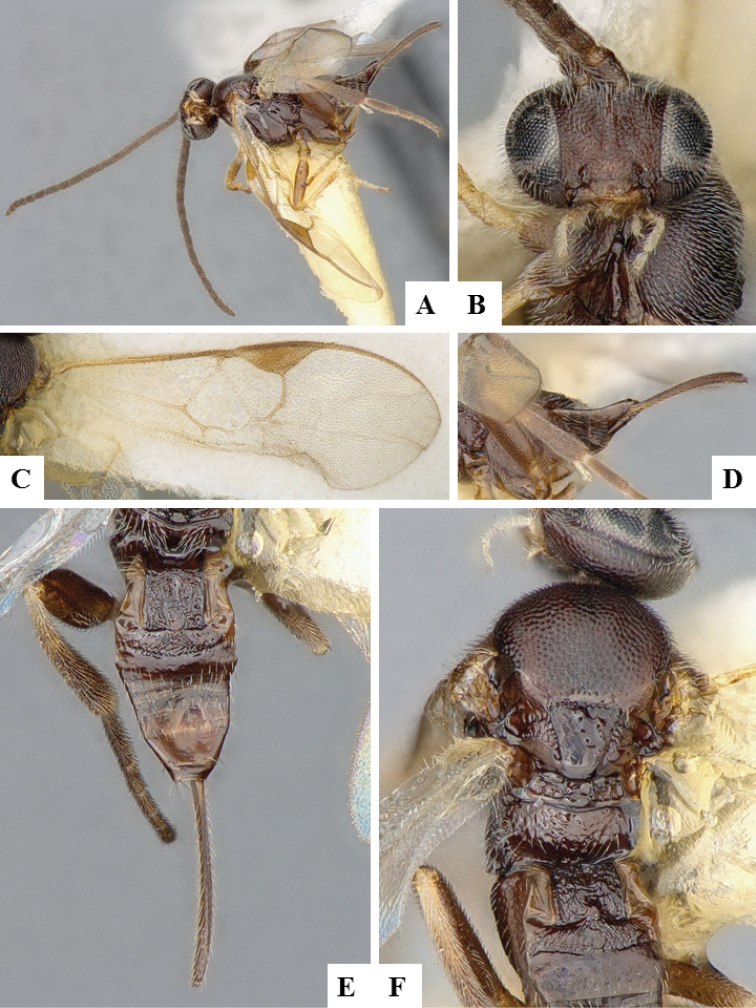
*Dolichogenidea thujae*. **A** Habitus, lateral **B** Head, frontal **C** Wings **D** Ovipositor sheaths and ovipositor **E** Metasoma, dorsal **F** Head and mesosoma, dorsal.

#### Dolichogenidea jft02

Taxon classificationAnimaliaHymenopteraBraconidae

##### Distribution.

NEA.

##### Notes.

This species corresponds in BOLD to BIN BOLD:AAA4312, with all specimens collected in North America.

##### Material examined.

Ontario, mixed forest, 45.2347 -75.624, 7-19.vi.2007, A. Bennett, Voucher Code: CAM0534.

#### Dolichogenidea jft12

Taxon classificationAnimaliaHymenopteraBraconidae

##### Distribution.

NEA.

##### Notes.

This species corresponds in BOLD to BIN BOLD:AAC9784, with all specimens collected in eastern Canada.

##### Material examined.

Ontario, Ottawa, city garden, 45.3561 -75.707, 1.ix.2007, H. Goulet, Voucher Code: CAM0044, CAM0087, CAM0149; Woodlawn, 45.375 -76.083, 8.ix.2008, L. Masner, Voucher Code: MIC000606.

#### Dolichogenidea jft13

Taxon classificationAnimaliaHymenopteraBraconidae

##### Distribution.

NEA.

##### Notes.

This species corresponds in BOLD to BIN BOLD:AAC0737, with all specimens collected in Canada.

##### Material examined.

Ontario, Ottawa, city garden, 45.3561 -75.707, 13.vii.2007, H. Goulet, Voucher Code: CAM0057; 23.vii.2007, H. Goulet, Voucher Code: CAM0048.

#### Dolichogenidea jft14

Taxon classificationAnimaliaHymenopteraBraconidae

##### Distribution.

NEA.

##### Notes.

This species corresponds in BOLD to BIN BOLD:AAA5986, with all specimens collected in Canada.

##### Material examined.

Ontario, Ottawa, city garden, 45.3561 -75.7069, 16.vii.2009, L. Masner, Voucher Code: CNCH1020.

#### Dolichogenidea jft18

Taxon classificationAnimaliaHymenopteraBraconidae

##### Distribution.

NEA.

##### Notes.

This species corresponds in BOLD to BIN BOLD:AAH2146, with all specimens collected in eastern Canada.

##### Material examined.

Ontario, Ottawa, city garden, 45.3561 -75.707, 13.vii.2007, H. Goulet, Voucher Code: CAM0060.

#### Glyptapanteles militaris

Taxon classificationAnimaliaHymenopteraBraconidae

(Walsh, 1861)

##### Distribution.

NEA, NEO, PAL.

##### Material examined.

Ontario, Ottawa, City Garden, 45.381015 -75.715397, Malaise trap, 10.viii-1.ix.2007, H. Goulet, Voucher Code: CNC482042, CNC482043, CNC482044, CNC482045, CNC482046, CNC482047, CNC482048, CNC482049, CNC482050, CNC482051, CNC482052, CNC482053, CNC482054, CNC482055, CNC482056, CNC482057, CNC482058, CNC482059, CNC482060, CNC482061, CNC482062, CNC482063, CNC482064, CNC482065, CNC482066, CNC482067, CNC482068; 23-30.vii.2007, H. Goulet, Voucher Code: CNC482079, CNC482080, CNC482081, CNC482082, CNC482083, CNC482084, CNC482085, CNC482086, CNC482087, CNC482088, CNC482089, CNC482090, CNC482091, CNC482092, CNC482093, CNC482094, CNC482095, CNC482096, CNC482097, CNC482098, CNC482099, CNC482100, CNC482101, CNC482102, CNC482103, CNC482104, CNC482105, CNC482106, CNC482107, CNC482108, CNC482109, CNC482110, CNC482111, CNC482112, CNC482113, CNC482114, CNC482115, CNC482116, CNC482117, CNC482118, CNC482119, CNC482120, CNC482121, CNC482122, CNC482123, CNC482124, CNC482125, CNC482126, CNC482127, CNC482128, CNC482129, CNC482130, CNC482131, CNC482132, CNC482133, CNC482134; 24-30.v.2007, H. Goulet, Voucher Code: CNC482073, CNC482074, CNC482075, CNC482076; 26.vi-13.vii.2007, H. Goulet, Voucher Code: CNC482078; 30.vii-10.viii.2007, H. Goulet, H.Goulet, Voucher Code: CNC481889, CNC481890, CNC481891, CNC481928, CNC481929, CNC481930, CNC481931, CNC481932, CNC481933, CNC481934, CNC481935, CNC481936, CNC481937, CNC481938, CNC481939, CNC481940, CNC481941, CNC481942, CNC481943, CNC481944, CNC481945, CNC481946, CNC481947, CNC481948, CNC481949, CNC481950, CNC481951, CNC481952, CNC481953, CNC481954, CNC481955, CNC481956, CNC481957, CNC481958, CNC481959, CNC481960, CNC481961, CNC481962, CNC481963, CNC481964, CNC481965, CNC481966, CNC481967, CNC481968, CNC481969, CNC481970, CNC481971, CNC481972, CNC481973, CNC481974, CNC481975, CNC481976, CNC481977, CNC481978, CNC481979, CNC481980, CNC481981, CNC481982, CNC481983, CNC481984, CNC481985, CNC481986, CNC481987, CNC481988, CNC481997, CNC481998, CNC481999, CNC482000, CNC482001, CNC482002, CNC482003, CNC482004, CNC482005, CNC482006, CNC482007, CNC482008, CNC482009, CNC482010, CNC482011, CNC482012, CNC482013, CNC482014, CNC482015, CNC482016, CNC482017, CNC482018, CNC482019, CNC482020, CNC482021, CNC482022, CNC482023, CNC482024, CNC482025, CNC482026, CNC482027, CNC482028, CNC482029, CNC482030, CNC482031, CNC482032, CNC482033, CNC482034, CNC482035, CNC482036, CNC482037, CNC482038, CNC482039, CNC482040, CNC482041, CNC481878, CNC481879, CNC481880, CNC481881, CNC481882, CNC481883, CNC481884, CNC481885, CNC481886, CNC481887, CNC481888; 8-16.vi.2007, H. Goulet, Voucher Code: CNC482077; Ottawa, city garden, 45.381015 -75.715397, Malaise trap, 1-19.ix.2007, H. Goulet, Voucher Code: CNC475269, CNC475270, CNC475271, CNC475272; 10.viii-1.ix.2007, H. Goulet, Voucher Code: CNC481892, CNC481893, CNC481894, CNC481895, CNC481896, CNC481897, CNC481898, CNC481899, CNC481900, CNC481901, CNC481902, CNC481903, CNC481904, CNC481905, CNC481906, CNC481907, CNC481908, CNC481909, CNC481910, CNC481911, CNC481912, CNC481913, CNC481914, CNC481915, CNC481916, CNC481917, CNC481918, CNC481919, CNC481920, CNC481921, CNC481922, CNC481923, CNC481924, CNC481925, CNC481926, CNC481927; 23-30.vii.2007, H. Goulet, Voucher Code: CNC475244, CNC475245, CNC475246, CNC475247, CNC475248, CNC475249, CNC475250, CNC475251, CNC475252, CNC475253, CNC475254, CNC475255, CNC475256, CNC475257, CNC475258, CNC475259, CNC475260, CNC475261, CNC475262, CNC475263, CNC475264, CNC475265; 24-30.v.2007, H. Goulet, Voucher Code: CNC481852, CNC481853, CNC481854; 26.vi-13.vii.2007, H. Goulet, Voucher Code: CNC481855; 30.vii-10.viii.2007, H. Goulet, Voucher Code: CNC475238, CNC475239, CNC475240, CNC475241, CNC475242, CNC475243, CNC475273; 8-16.vi.2007, H. Goulet, Voucher Code: CNC475266, CNC475267, CNC475268; Ottawa, 45.381015 -75.715397, Malaise trap, 10.viii-1.ix.2007, H. Goulet, Voucher Code: CNC481856, CNC481857, CNC481858, CNC481859, CNC481860, CNC481861, CNC481862, CNC481863, CNC481864, CNC481865, CNC481866, CNC481867, CNC481868, CNC481869, CNC481870, CNC481871, CNC481872, CNC481873, CNC481874, CNC481875, CNC481876, CNC481877; 21.vii.1957, Guppy, J.C., J. C. Guppy, J.C. Guppy, Voucher Code: CNC280985, CNCHYM01317, CNC475225, CNC475226, CNC475227, CNC475228, CNC475229, CNC475230, CNC475231, CNC475232, CNC475233, CNC475234, CNC475235, CNC475236, CNC475237.

#### Glyptapanteles pallipes

Taxon classificationAnimaliaHymenopteraBraconidae

(Reinhard, 1880)

Fig. 25

##### Distribution.

NEA, PAL.

##### Material examined.

Ontario, Ottawa, 45.406631 -75.701407, 30.ix.1925, D. D. Gray, ~, Voucher Code: CNC482135, CNC482136, CNC482137, CNC482138, CNC482139, CNC482140, CNC482141, CNC482142, CNC482143, CNC482144, CNC482145, CNC482146, CNC482147, CNC482148, CNC482149, CNC482150, CNC482161, CNC482162, CNC482163, CNC482164, CNC482165, CNC482166, CNC482167, CNC482168, CNC482169, CNC482170, CNC482171, CNC482172, CNC482173, CNC482174, CNC482175, CNC482176, CNC482177, CNC482178, CNC482179, CNC482180, CNC482181, CNC482182, CNC482183, CNC482184, CNC482185, CNC482186, CNC482187, CNC482188, CNC482189, CNC482190, CNC482191, CNC482192, CNC482193, CNC482194, CNC482195, CNC482196, CNC482197, CNC482198, CNC482199, CNC482200, CNC482201, CNC482202, CNC482203, CNC482204, CNC482205, CNC482206, CNC482207, CNC482208, CNC482209, CNC482210, CNC482211, CNC482212, CNC482213, CNC482214, CNC482215, CNC482216, CNC482217, CNC482218, CNC482219, CNC482220, CNC482151; Quebec, Cantley, Gatineau Co., 45.507888 -75.761995, 22.vii.1973, A.B. Cruins, M. B. Guins, Voucher Code: CNCHYM01322, CNC482152, CNC482153, CNC482154, CNC482155, CNC482156, CNC482157, CNC482158, CNC482159, CNC482160.

**Figure 25.**
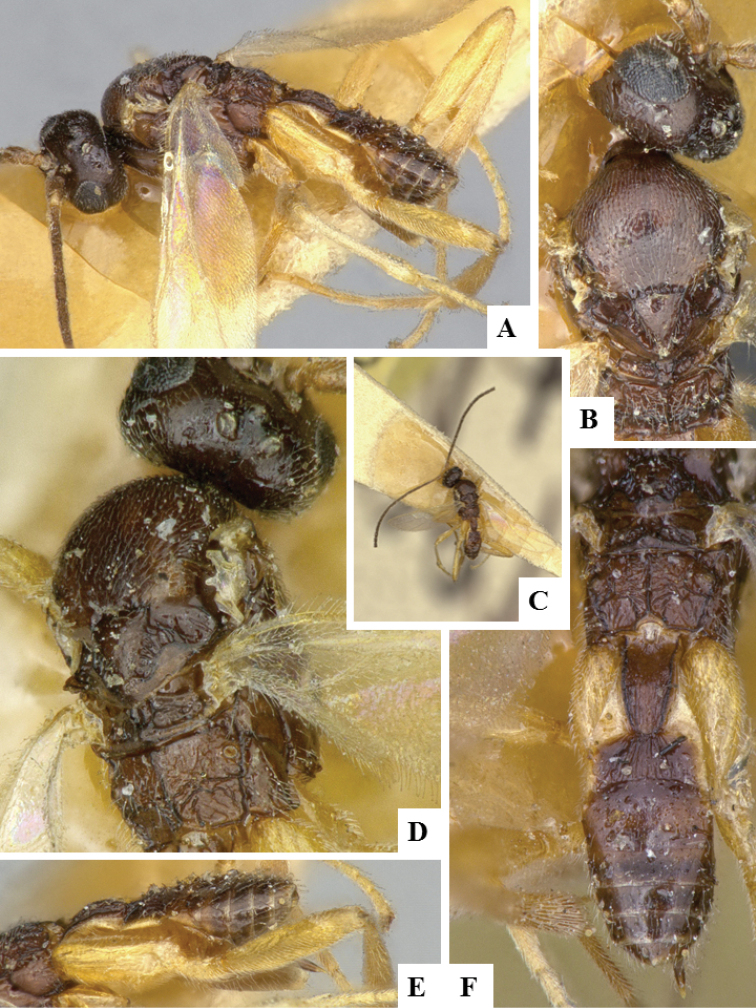
*Glyptapanteles pallipes*. **A** Habitus, lateral **B** Head and mesosoma, dorsal **C** Habitus, dorsal **D** Head and mesosoma, dorso-laterally **E** Metasoma, lateral **F** Metasoma, dorsal.

#### Glyptapanteles sp.

Taxon classificationAnimaliaHymenopteraBraconidae

##### Distribution.

NEA.

##### Notes.

This species corresponds in BOLD to BIN BOLD:AAA4784, with all specimens collected in North America.

##### Material examined.

Ontario, mixed forest, 45.2347 -75.624, 19-29.vi.2007, A. Bennett, Voucher Code: CAM0554; 7-19.vi.2007, A. Bennett, Voucher Code: CAM0555, CAM0556, CAM0557, CAM0558; Ottawa, city garden, 45.3561 -75.707, 30.vii.2007, H. Goulet, Voucher Code: CAM0264, CAM0265, CAM0274; Woodlawn, 45.375 -76.083, 17.viii.2008, L. Masner, Voucher Code: MIC000613; 6.viii.2008, L. Masner, Voucher Code: MIC000607, MIC000608, MIC000609, MIC000610, MIC000611, MIC000612; 8.ix.2008, L. Masner, Voucher Code: MIC000614, MIC000615, MIC000616, MIC000617

#### Hygroplitis melligaster

Taxon classificationAnimaliaHymenopteraBraconidae

(Provancher, 1886)

Fig. 26

##### Distribution.

NEA.

##### Material examined.

Ontario, 2 km SW of Innisville, 45.054942 -76.250619, 26.vi.1991, Sharkey & Read, Voucher Code: GOU0314; Aylmer West, 45.395345 -75.844876, Malaise trap, 1-3.viii.1972, Voucher Code: CNC482223; 20-24.vii.1972, Voucher Code: CNC482221, CNC482222; 45.4 -75.85, 22.vii.1972, Voucher Code: CNCHYM01376; Bells Corners, 45.322247 -75.833249, 26.vii.1939, O. Peck, Voucher Code: CNC482224, CNC482225; Galetta, 45.433392 -76.250086, 22.vii.1942, G. S. Walley, Voucher Code: CNC482226; Innisville, 45.055468 -76.250497, 12.vii.1963, W. R. M. Mason, Voucher Code: CNC482228; 16.vii.1963, W. R. M. Mason, Voucher Code: CNC482229; 18.vii.1963, W. R. M. Mason, Voucher Code: CNC482230, CNC482231, CNC482232; 22.vii.1963, W. R. M. Mason, Voucher Code: CNC482233, CNC482234, CNC482235, CNC482236, CNC482237, CNC482238, CNC482239, CNC482240, CNC482248; 25.vii.1963, W. R. M. Mason, Voucher Code: CNC482241, CNC482242, CNC482243, CNC482244; 28.vii.1963, W. R. M. Mason, Voucher Code: CNC482245, CNC482246, CNC482247; Kemptville, 45.016409 -75.64602, 28.vii.1983, W. Mason, Voucher Code: CNCHYM01375; 45.016409 -75.646449, Maple Forest, Malaise trap, 19-26.vii.1983, W. Mason, Voucher Code: CNC482259, CNC482260; 26-31.vii.1983, W. Mason, Voucher Code: CNC482261, CNC482262; Mer Bleue, Ottawa, 45.393594 -75.512139, 9.vii.1982, L. Dumouchel, Voucher Code: CNCHYM01378; Ottawa, 45.406631 -75.701407, Voucher Code: CNC482227; Stittsville, 45.258675 -75.921130, 18.vii.1963, W. R. M. Mason, Voucher Code: CNC482257; 2.vii.1963, W. R. M. Mason, Voucher Code: CNC482249; 22.vii.1963, W. R. M. Mason, Voucher Code: CNC482250; 25.vii.1963, W. R. M. Mason, Voucher Code: CNC482251, CNC482252; 28.vii.1963, W. R. M. Mason, Voucher Code: CNC482253; 9.viii.1963, W. R. M. Mason, Voucher Code: CNC482254, CNC482255, CNC482256; 45.258796 -75.92113, 22.viii.1963, W. R. M. Mason, Voucher Code: CNCHYM01364; 45.259037 -75.920958, 22.viii.1963, W. R. M. Mason, Voucher Code: CNC280997.

**Figure 26.**
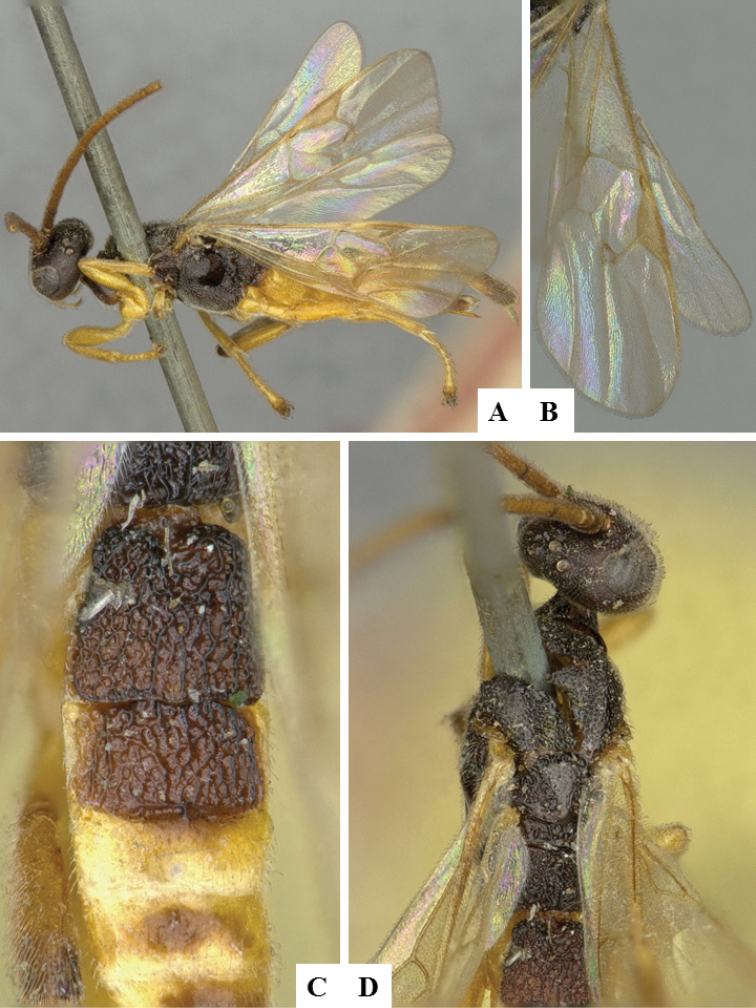
*Hygroplitis melligaster*. **A** Habitus, lateral **B** Wings **C** Metasoma (partially), dorsal **D** Head and mesosoma, dorsal.

#### Hypomicrogaster ecdytolophae

Taxon classificationAnimaliaHymenopteraBraconidae

(Muesebeck, 1922)

##### Distribution.

NEA, NEO.

##### Notes.

Valerio and Whitfield (2015) synonymized this species under *Hypomicrogaster zonaria*. However, based on the specimens and associated data that we have been able to see (e.g., DNA and host records), *Hypomicrogaster zonaria* (*sensu* Valerio and Whitfield 2015) seems to actually comprise several, distinct species. Pending future work on the New World fauna of *Hypomicrogaster*, we are considering in this paper *Hypomicrogaster ecdytolophae* as separate from *Hypomicrogaster zonaria*.

##### Material examined.

Ontario, Merivale, 45.340734 -75.727462, 19.vii.1943, G. S. Walley, Voucher Code: CNCHYM01386; 45.340749 -75.727473, 19.vii.1943, G.S. Walley, Voucher Code: CNC280999; Ottawa, city garden, 45.356100 -75.707000, 19.x.2007, H. Goulet, Voucher Code: CAM0260; Ottawa, 45.406631 -75.701407, 8.iii.1939, C. H. Young, Voucher Code: CNC482258.

#### Hypomicrogaster zonaria

Taxon classificationAnimaliaHymenopteraBraconidae

(Say, 1836)

##### Distribution.

NEA.

##### Notes.

In this paper we are considering this species as separate from *Hypomicrogaster ecdytolophae* (see comments above).

##### Material examined.

Ontario, Aylmer West, 45.395345 -75.844876, Malaise trap, 13-17.vii.1972, Voucher Code: CNC482280; 17-20.vii.1972, Voucher Code: CNC482281; 27-31.vii.1972, Voucher Code: CNC482279; 3-8.viii.1972, Voucher Code: CNC482278; Flint Hill nr. Kemptville, 45.016346 -75.646535, 16-23.viii.1983, Mason, Voucher Code: CNC482271, CNC482272, CNC482273, CNC482274, CNC482275, CNC482276, CNC482277; Kemptville, 45.016409 -75.64602, 30.viii.1983, W. Mason, Voucher Code: CNCHYM01441; 45.016409 -75.646449, Maple Forest, Malaise trap, 1-6.ix.1983, W. Mason, Voucher Code: CNC482301, CNC482302, CNC482303, CNC482304; 19-26.vii.1983, W. Mason, Voucher Code: CNC482294, CNC482295, CNC482296; 26-31.vii.1983, W. Mason, Voucher Code: CNC482291, CNC482292, CNC482293; Maple Forest, ~, Malaise trap, 29.vii.1983, W. Mason, Voucher Code: CNC482305, CNC482306, CNC482289, CNC482290; Maple Forest, Malaise trap, 30.vii.1983, W. Mason, Voucher Code: CNC482285, CNC482286, CNC482287, CNC482288; 6-13.ix.1983, W. Mason, Voucher Code: CNC482297, CNC482298, CNC482299, CNC482300; 9-16.vii.1983, W. Mason, Voucher Code: CNC482307, CNC482308, CNC482309, CNC482310, CNC482311, CNC482312, CNC482313, CNC482314, CNC482315, CNC482316, CNC482317, CNC482318, CNC482319, CNC482320, CNC482321, CNC482322, CNC482323, CNC482324, CNC482325, CNC482326; Nr. Kemptville, 45.016409 -75.646449, Sunny dec. for., 16-23.viii.1983, Dumouchel & Perkins, Voucher Code: CNC482282, CNC482283, CNC482284; Stittsville, Carleton Co., 45.258675 -75.921130, 23.viii.1977, M. Sanborne, Voucher Code: CNC482267; 24.ix.1981, M.Sanborne, Voucher Code: CNC482265, CNC482266; 26.viii.1977, M. Sanborne, Voucher Code: CNC482268; Stittsville, 45.258675 -75.921130, 3.ix.1963, W. R. M. Mason, Voucher Code: CNC482263, CNC482264; Quebec, Gatineau Park, Camp Fortune, 45.509059 -75.851290, Malaise trap, 16.viii.1982, L. Masner, Voucher Code: CNC482269, CNC482270.

#### Hypomicrogaster jft30

Taxon classificationAnimaliaHymenopteraBraconidae

##### Distribution.

NEA.

##### Notes.

This species corresponds in BOLD to BIN BOLD:AAD0217, with all specimens collected in southern Ontario.

##### Material examined.

Ontario, Ottawa, city garden, 45.356100 -75.707000, 1.xi.2007, H. Goulet, Voucher Code: CAM0259.

#### Hypomicrogaster jft31

Taxon classificationAnimaliaHymenopteraBraconidae

##### Distribution.

NEA.

##### Notes.

This species corresponds in BOLD to BIN BOLD:ACF0950, with all specimens collected in North America.

##### Material examined.

Ontario, Woodlawn, 45.375000 -76.083000, 8.ix.2008, L. Masner, Voucher Code: MIC000623.

#### Hypomicrogaster sp.

Taxon classificationAnimaliaHymenopteraBraconidae

##### Distribution.

NEA.

##### Notes.

This species corresponds in BOLD to BIN BOLD:AAD0218, with all specimens collected in eastern Canada. This seems to be another species related to *Hypomicrogaster zonaria* (*sensu* Valerio and Whitfield 2015).

##### Material examined.

Ontario, Ottawa, city garden, 45.356100 -75.707000, 1.ix.2007, H. Goulet, Voucher Code: CAM0267.

#### Illidops sp. jft 18

Taxon classificationAnimaliaHymenopteraBraconidae

##### Distribution.

NEA.

##### Notes.

This is the only specimen of this genus known to us from Ottawa.

##### Material examined.

Ontario, Constance Bay, 45.486308 -76.073461, 30.v.1939, G. S. Walley, Voucher Code: CNCHYM01550.

#### Lathrapanteles heleios

Taxon classificationAnimaliaHymenopteraBraconidae

Williams, 1985

Fig. 27

##### Distribution.

NEA.

##### Notes.

The status of this species as a potential member of the Species Candidate Lists of COSEWIC was assessed by Fernandez Triana (2014).

##### Material examined.

Ontario, Aylmer West, 45.400000 -75.850000, 27.viii.1972, Voucher Code: CNCHYM01571, CNCHYM01574; Mer Bleue, Ottawa, 45.393578 -75.512127, 10.vi.1975, H.J. Teskey, , 45.393585 -75.512138, 10.vi.1975, H. J. Teskey, Voucher Code: cnc482327, cnc482328, cnc482329, cnc482330, cnc482331, cnc482332, cnc482333, cnc482334, cnc482335, cnc482336, cnc482337, cnc482338, cnc482339, cnc482340, cnc482341, cnc482342, cnc482343, cnc482344, cnc482345, cnc482346; 45.393593 -75.512138, 10.vi.1975, H. J. Teskey, Voucher Code: CNCHYM01570, CNCHYM01573; Mer Bleue, 45.393578 -75.512127, 10.vi.1975, Teskay, H.J. , Voucher Code: CNC281023; Ottawa, city garden, 45.3561 -75.7069, 10.viii-1.ix.2007, H. Goulet, Voucher Code: CAM0895, CAM0903, CAM0904, CAM0905; 45.3561 -75.707, 1.ix.2007, H. Goulet, Voucher Code: CAM0266.

**Figure 27.**
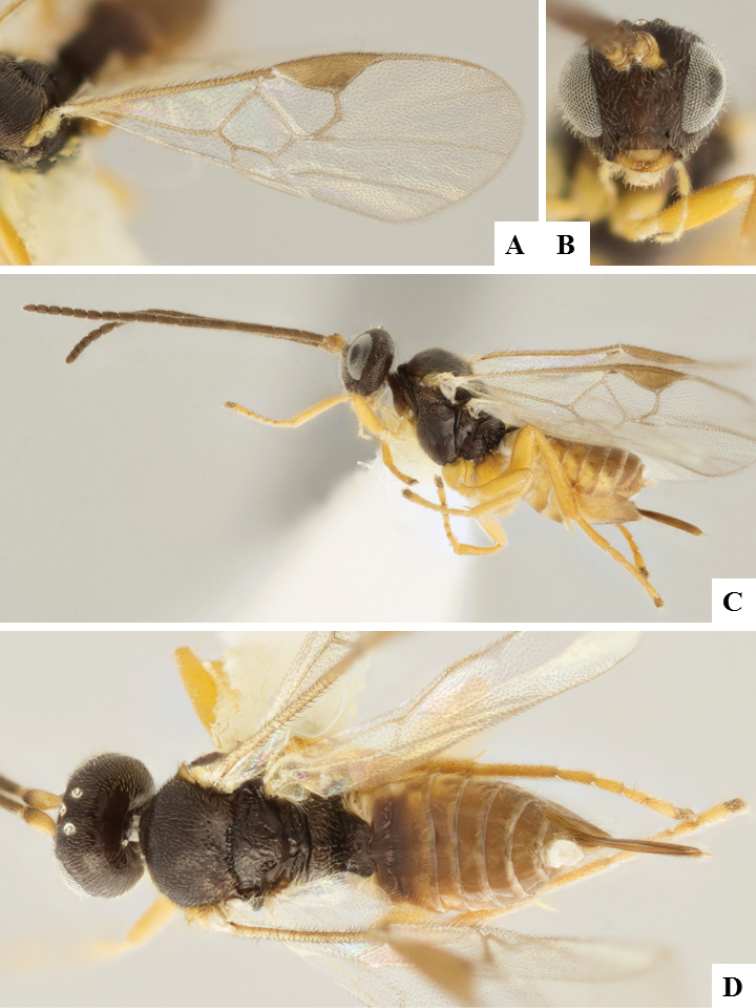
*Lathrapanteles heleios*. **A** Wings **B** Head, frontal **C** Habitus, lateral **D** Habitus, dorsal.

#### Lathrapanteles papaipemae

Taxon classificationAnimaliaHymenopteraBraconidae

(Muesebeck, 1921)

##### Distribution.

NEA.

##### Material examined.

Ontario, Aylmer West, 45.400000 -75.850000, Malaise trap, ~, 27.viii.1972, Voucher Code: CNCHYM01576, CNCHYM01575.

#### Microgaster canadensis

Taxon classificationAnimaliaHymenopteraBraconidae

Muesebeck, 1922

##### Distribution.

NEA.

##### Material examined.

Ontario, Constance Bay, 45.486218 -76.073461, 21.vii.1933, G. S. Walley, Voucher Code: cnc482347; Corkery, 45.284686 -76.102742, 3.vii.1948, F.I.S., Voucher Code: CNC482348.

#### Microgaster gelechiae

Taxon classificationAnimaliaHymenopteraBraconidae

Riley, 1869

Fig. 28

##### Distribution.

NEA.

##### Material examined.

Ontario, Jockvale, 45.266494 -75.74418, 8.v.1943, J. McDunmough, Voucher Code: CNCHYM01616; 45.266524 -75.744180, 8.v.1943, McDunnough, J., Voucher Code: CNC281031; Ottawa, 45.382500 -75.713700, 1.v.1975, Voucher Code: CNCHYM01615.

**Figure 28.**
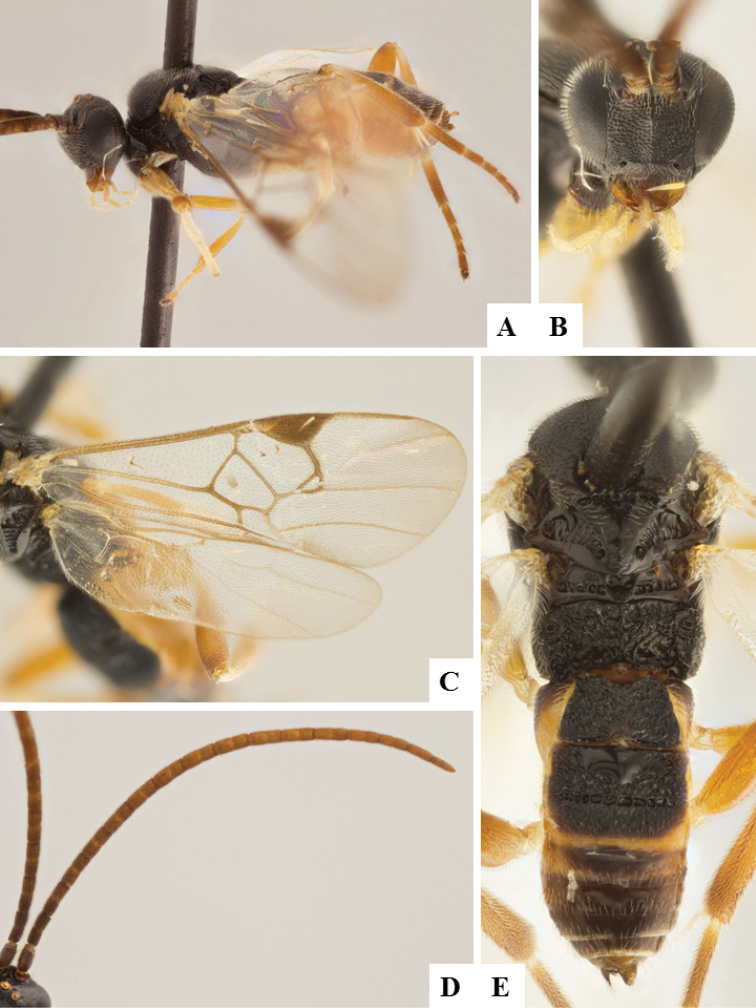
*Microgaster gelechiae*. **A** Habitus, lateral **B** Head, frontal **C** Wings **D** Antenna **E** Mesosoma and metasoma, dorsal.

#### Microgaster leechi

Taxon classificationAnimaliaHymenopteraBraconidae

Walley, 1935

Fig. 29

##### Distribution.

NEA.

##### Material examined.

Ontario, Innisville, 45.054942 -76.250619, 6.viii.1963, W. R. M. Mason, Voucher Code: CNCHYM01630; 45.055468 -76.250497, 28.vii.1963, W. R. M. Mason, Voucher Code: CNC482351; Merivale, 45.325948 -75.719082, 25.vii.1930, J. J. deGryse, Voucher Code: CNC482349; Ottawa, 45.406631 -75.701407, 27.vii.1947, W. R. M. Mason, Voucher Code: CNC482353; Stittsville, 45.258675 -75.921130, 22.viii.1963, W. R. M. Mason, Voucher Code: CNC482350; 45.258796 -75.92113, 3.ix.1963, W. R. M. Mason, Voucher Code: CNCHYM01631; Quebec, Hull, 45.428550 -75.714554, 16.viii.1894, Voucher Code: CNC482352.

**Figure 29.**
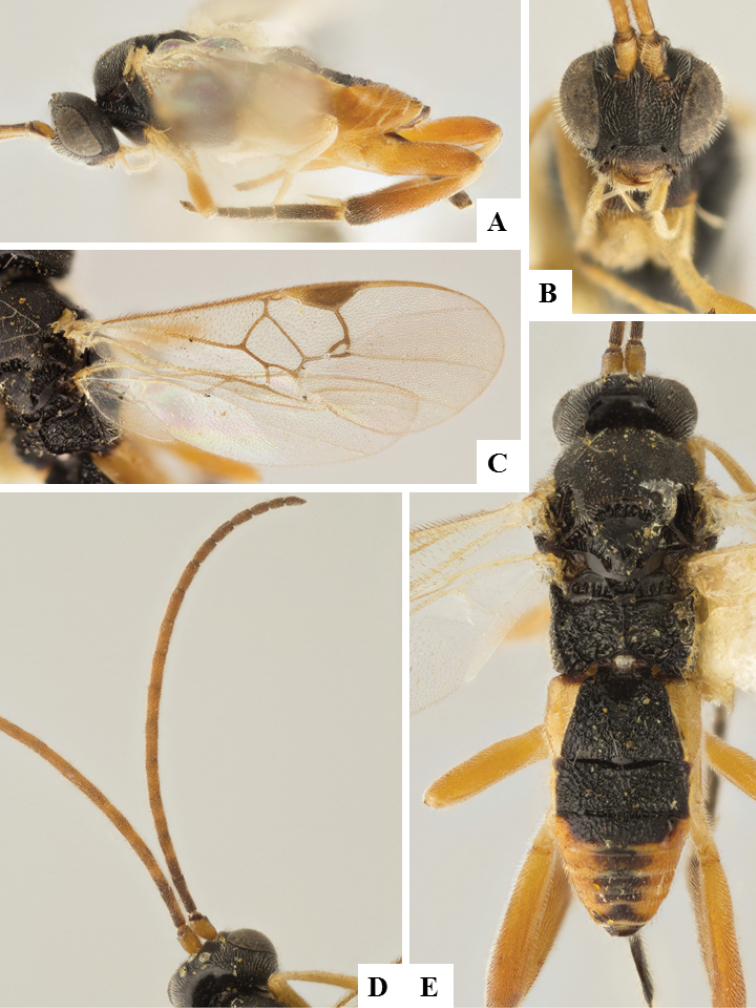
*Microgaster leechi*. **A** Habitus, lateral **B** Head, frontal **C** Wings **D** Antenna **E** Mesosoma and metasoma, dorsal.

#### Microgaster jft09

Taxon classificationAnimaliaHymenopteraBraconidae

##### Distribution.

NEA.

##### Notes.

This species corresponds in BOLD to BIN BOLD:ABZ2766, with all specimens collected in Canada.

##### Material examined.

Ontario, 5 km NW of Almonte, Hwy 49, Burnt Land, Alvar Prov. Park, Almonte, 45.255 -76.14, 29.v.2008, Goulet & Fernandez, Voucher Code: CAM0331; North Gower to Smith Falls, 1 km N of Rd 6 & Montague Bdy Rd, 45.033 -75.9, 15.vi.2004, Bennett & Barnes, Voucher Code: HYM00001332; Ottawa, city garden, 45.356 -75.707, 24-30.v.2007, H. Goulet, Voucher Code: CAM0934.

#### Microgaster jft10

Taxon classificationAnimaliaHymenopteraBraconidae

##### Distribution.

NEA, PAL.

##### Notes.

Specimens from both Europe and North America have rendered similar DNA barcodes, but we have not been able to match them to any described species – it remains as undescribed for the time being. This species corresponds in BOLD to BIN BOLD:ACE8790.

##### Material examined.

Ontario, Ottawa, city garden, 45.356 -75.707, 16-26.vi.2007, H. Goulet, Voucher Code: CAM0926, CAM0927, CAM0928; 8-16.vi.2007, H. Goulet, Voucher Code: CAM0922, CAM0923, CAM0925.

#### Microgaster jft11

Taxon classificationAnimaliaHymenopteraBraconidae

##### Distribution.

NEA.

##### Notes.

This species corresponds in BOLD to BIN BOLD:AAH3530, with all specimens collected in Canada.

##### Material examined.

Ontario, Ottawa, city garden, 45.356 -75.707, 5.vi-2.vii.2008, H. Goulet, Voucher Code: CAM0920.

#### Microgaster jft14

Taxon classificationAnimaliaHymenopteraBraconidae

##### Distribution.

NEA.

##### Notes.

This species corresponds in BOLD to BIN BOLD:ABY6384, with all specimens collected in Canada.

##### Material examined.

Ontario, Ottawa, city garden, 45.356 -75.707, 26.vi-13.vii.2007, H. Goulet, Voucher Code: CAM0931.

#### Microgaster jft17

Taxon classificationAnimaliaHymenopteraBraconidae

##### Distribution.

NEA.

##### Notes.

This species corresponds in BOLD to BIN BOLD:AAA7886, with all specimens collected in North America. See comments on species jft21 below.

##### Material examined.

Ontario, 2 km SW of Innisville, 45.054942 -76.250619, 19.vi.1991, Denis & Read, Voucher Code: GOU0259; 26.vi.1991, Denis & Read, Voucher Code: GOU0273; mixed forest, 45.235 -75.624, 19-29.vi.2007, A. Bennett, Voucher Code: CAM0561; 29.vi-16.vii.2007, A. Bennett, Voucher Code: CAM0563; 7-19.vi.2007, A. Bennett, Voucher Code: CAM0562; Ottawa, city garden, 45.356 -75.707, 10.viii-1.ix.2007, H. Goulet, Voucher Code: CAM0919; 13-23.vii.2007, H. Goulet, Voucher Code: CAM0932; 24-30.v.2007, H. Goulet, Voucher Code: CAM0933; 30.vii-10.viii.2007, H. Goulet, Voucher Code: CAM0921.

#### Microgaster jft21

Taxon classificationAnimaliaHymenopteraBraconidae

##### Distribution.

NEA.

##### Notes.

This species corresponds in BOLD to BIN BOLD:AAA7886, with all specimens collected in North America. *Microgaster* jft17 and jft21 seem to be close (based on morphology and DNA barcodes), but there are also some differences among specimens, and thus we have decided to keep them as different species for the time being.

##### Material examined.

Ontario, 2 km SW of Innisville, 45.054942 -76.250619, 19.vi.1991, Denis & Read, Voucher Code: GOU0260; Ottawa, city garden, 45.356 -75.707, 30.v-8.vi.2007, H. Goulet, Voucher Code: CAM0929; 8-16.vi.2007, H. Goulet, Voucher Code: CAM0924.

#### Microgaster jft23

Taxon classificationAnimaliaHymenopteraBraconidae

##### Distribution.

NEA.

##### Notes.

This species corresponds in BOLD to BIN BOLD:AAB8447, with all specimens collected in North America.

##### Material examined.

Ontario, Ottawa, city garden, 45.356100 -75.706900, 26.vi-13.vii.2007, H. Goulet, Voucher Code: CAM0930.

#### Microgaster nr. epagoges

Taxon classificationAnimaliaHymenopteraBraconidae

##### Distribution.

NEA.

##### Material examined.

Ontario, Mer Bleue, Ottawa, 45.393594 -75.512139, 19.vii.1963, J. R. Vockeroth, Voucher Code: CNCHYM01608; Stittsville, 45.258797 -75.921133, 10.ix.1963, W. R. M. Mason, Voucher Code: CNCHYM01613.

#### Microplitis autographae

Taxon classificationAnimaliaHymenopteraBraconidae

Muesebeck, 1922

##### Distribution.

NEA.

##### Material examined.

Ontario, Hazeldean, 45.300928 -75.893728, 24.vi.1946, Voucher Code: CNCHYM01708; Ottawa, 45.3825 -75.7137, 26.iv.1948, A. R. Brooks, Voucher Code: CNCHYM01713; 45.382500 -75.713700, 27.vii.1947, W. R. M. Mason, Voucher Code: CNCHYM01717; Quebec, Hull, 45.428311 -75.713353, 2.v.1945, G. S. Walley, Voucher Code: CNCHYM01714.

#### Microplitis gortynae

Taxon classificationAnimaliaHymenopteraBraconidae

Riley, 1881

##### Distribution.

NEA.

##### Material examined.

Ontario, Innisville, 45.054942 -76.250619, 12.v.1963, W. R. M. Mason, Voucher Code: CNCHYM01752.

#### Microplitis hyphantriae

Taxon classificationAnimaliaHymenopteraBraconidae

Ashmead, 1898

##### Distribution.

NEA.

##### Material examined.

Ontario, Mer Bleue, Ottawa, 45.393585 -75.512138, 11.vii.1923, F. Ide, Voucher Code: CNC482354.

#### Microplitis impressus

Taxon classificationAnimaliaHymenopteraBraconidae

(Wesmael, 1837)

##### Distribution.

NEA, PAL.

##### Material examined.

Quebec, Chelsea, 45.541075 -75.867938, 24.iv.1933, G. S. Walley, Voucher Code: CNCHYM01763; 24.v.1933, G. S. Walley, Voucher Code: CNCHYM01764; 45.541315 -75.867938, 21.iv.1933, G. S. Walley, Voucher Code: CNC482361, CNC482362, CNC482363.

#### Microplitis kewleyi

Taxon classificationAnimaliaHymenopteraBraconidae

Muesebeck, 1922

##### Distribution.

NEA.

##### Material examined.

Ontario, Ottawa, city garden, 45.3561 -75.7069, 10-13.x.2008, H. Goulet, Voucher Code: WMIC0210; 10.viii-1.ix.2007, H. Goulet, Voucher Code: WMIC0204, WMIC0205, WMIC0206, WMIC0215, WMIC0217, WMIC0220; 20-29.ix.2008, H. Goulet, Voucher Code: WMIC0211; 30.vii-10.viii.2007, H. Goulet, Voucher Code: WMIC0196; Voucher Code: WMIC0208; 45.3561 -75.707, 10.viii.2007, H. Goulet, Voucher Code: CAM0169; 19.ix.2007, H. Goulet, Voucher Code: CAM0195.

#### Microplitis maturus

Taxon classificationAnimaliaHymenopteraBraconidae

Weed, 1888

##### Distribution.

NEA.

##### Material examined.

Ontario, Ottawa, 45.406631 -75.701407, Voucher Code: CNC482364.

#### Microplitis plutellae

Taxon classificationAnimaliaHymenopteraBraconidae

Muesebeck, 1922

Fig. 30

##### Distribution.

NEA, OTL, PAL.

##### Material examined.

Ontario, Ottawa, CNC breeding program, Ottawa, 45.3825 -75.7137, 31.iii.2008, Jose L. Fernandez Triana, Voucher Code: CPWH-0014, CPWH-0015, CPWH-0016, CPWH-0017, CPWH-0018, CPWH-0019, CPWH-0020, CPWH-0021, CPWH-0022, CPWH-0023, CPWH-0024, CPWH-0025, CPWH-0026, CPWH-0027, CPWH-0028, CPWH-0029, CPWH-0030, CPWH-0031, CPWH-0032; Ottawa, city garden, 45.356 -75.707, 10.viii-1.ix.2007, H. Goulet, Voucher Code: WMIC0212; Voucher Code: WMIC0192; Ottawa, 45.382500 -75.713700, 1953, D. Harcourt, Voucher Code: CNCHYM01833; 45.406631 -75.701407, 1953, D. Harcourt, Voucher Code: CNC482365, CNC482366, CNC482367, CNC482368, CNC482369.

**Figure 30.**
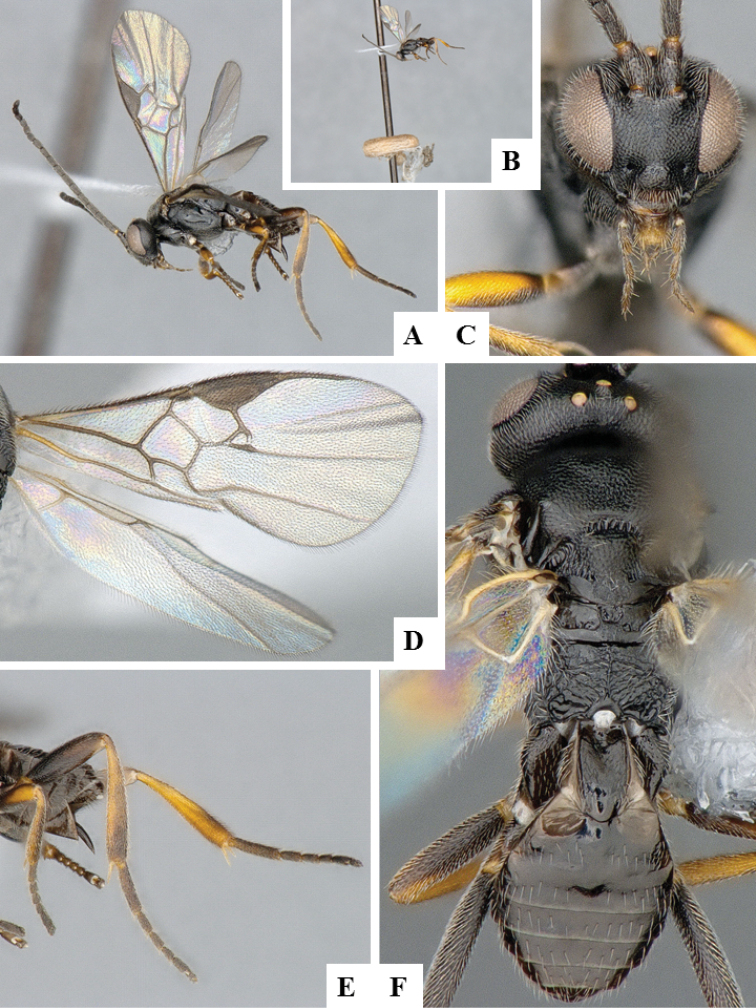
*Microplitis plutellae*. **A** Habitus, lateral **B** Glued specimen and cocoon **C** Head, frontal **D** Wings **E** Ovipositor sheaths **E** Habitus, dorsal.

#### Microplitis scutellatus

Taxon classificationAnimaliaHymenopteraBraconidae

Muesebeck, 1922

##### Distribution.

NEA.

##### Material examined.

Ontario, Carlsbad Springs, 45.369254 -75.456097, 5.iii.1941, Voucher Code: CNCHYM01845; Ottawa, 45.406631 -75.701407, Voucher Code: CNC482370.

#### Microplitis varicolor

Taxon classificationAnimaliaHymenopteraBraconidae

Viereck, 1917

##### Distribution.

NEA.

##### Notes.

DNA barcodes of some specimens from North America identified as *Microplits varicolor* sometimes match DNA barcodes of some European specimens identified as *Microplits mediator* (Haliday, 1834). This topic needs to be investigated further, but for the time being we are considering the two species as separate.

##### Material examined.

Ontario, 5 km NW of Almonte, Hwy 49, Burnt Land, Alvar Prov. Park, Almonte, 45.2549 -76.14, 29.v.2008, Goulet & Fernandez, Voucher Code: CAM0321; mixed forest, 45.2347 -75.624, 7-19.vi.2007, A. Bennett, Voucher Code: CAM0552; Ottawa, city garden, 45.356 -75.707, 28.vii.2009, L. Masner, Voucher Code: CNCH1023; 45.3561 -75.7069, 10.viii-1.ix.2007, H. Goulet, Voucher Code: WMIC0201, WMIC0203, WMIC0219, WMIC0222, WMIC0223, WMIC0224, WMIC0225, WMIC0226; 30.vii-10.viii.2007, H. Goulet, Voucher Code: WMIC0193, WMIC0194, WMIC0195, WMIC0197; 45.3561 -75.707, 10.viii.2007, H. Goulet, Voucher Code: CAM0091, CAM0093, CAM0094, CAM0095, CAM0170, CAM0171, CAM0172, CAM0173, CAM0175, CAM0176, CAM0177, CAM0178, CAM0248; 13.vii.2007, H. Goulet, Voucher Code: CAM0233; 16.v.2007, H. Goulet, Voucher Code: CAM0249, CAM0250; 16.vi.2007, H. Goulet, Voucher Code: CAM0191; 19.ix.2007, H. Goulet, Voucher Code: CAM0192, CAM0193, CAM0194, CAM0196, CAM0197, CAM0200, CAM0201, CAM0202; 19.x.2007, H. Goulet, Voucher Code: CAM0206, CAM0207, CAM0208, CAM0209; 30.v.2007, H. Goulet, Voucher Code: CAM0205, CAM0211, CAM0216, CAM0218, CAM0220, CAM0222, CAM0223, CAM0227; 30.vii.2007, H. Goulet, Voucher Code: CAM0180, CAM0182, CAM0183, CAM0185, CAM0186, CAM0187, CAM0188, CAM0189, CAM0190, CAM0235, CAM0236, CAM0237, CAM0239, CAM0240, CAM0241, CAM0243, CAM0244; 45.356100 -75.706900, H. Goulet, Voucher Code: WMIC0209; Ottawa, 45.406631 -75.701407, Dow’s Swamp, 3.viii.1947, W. R. M. Mason, Voucher Code: CNC482371; Woodlawn, 45.3754 -76.0827, 6.viii.2008, L. Masner, Voucher Code: MIC000620.

#### Microplitis jft07

Taxon classificationAnimaliaHymenopteraBraconidae

##### Distribution.

NEA.

##### Notes.

This species corresponds in BOLD to BIN BOLD:AAC0025, with all specimens collected in eastern Canada.

##### Material examined.

Ontario, mixed forest, 45.2347 -75.624, 7-19.vi.2007, A. Bennett, Voucher Code: CAM0551.

#### Microplitis jft16

Taxon classificationAnimaliaHymenopteraBraconidae

##### Distribution.

NEA.

##### Notes.

This species corresponds in BOLD to BIN BOLD:AAA2409, with all specimens collected in North America.

##### Material examined.

Ontario, Ottawa, Central Experimental Farm, 45.382500 -75.713700, 12.v.2000, Black & Goulet, Voucher Code: GOU0219; Ottawa, city garden, 45.356 -75.707, 10.viii-1.ix.2007, H. Goulet, Voucher Code: WMIC0200, WMIC0216, WMIC0218; 10.viii.2007, H. Goulet, Voucher Code: CAM0092, CAM0174; 13.vii.2007, H. Goulet, Voucher Code: CAM0234; 19.ix.2007, H. Goulet, Voucher Code: CAM0198, CAM0199; 30.v.2007, H. Goulet, Voucher Code: CAM0203, CAM0204, CAM0210, CAM0212, CAM0213, CAM0215, CAM0224, CAM0225, CAM0226, CAM0229, CAM0230; 30.vii-10.viii.2007, H. Goulet, Voucher Code: WMIC0198, WMIC0199; 30.vii.2007, H. Goulet, Voucher Code: CAM0179, CAM0181, CAM0238, CAM0242, CAM0245, CAM0246, CAM0247; 45.356000 -75.707000, H. Goulet, Voucher Code: WMIC0207; 45.3561 -75.7069, H. Goulet, Voucher Code: WMIC0191; Woodlawn, 45.3754 -76.0827, 8.ix.2008, L. Masner, Voucher Code: MIC000618, MIC000621.

#### Microplitis jft19

Taxon classificationAnimaliaHymenopteraBraconidae

##### Distribution.

NEA.

##### Notes.

This species corresponds in BOLD to BIN BOLD:AAD2718, with all specimens collected in Ottawa.

##### Material examined.

Ontario, 5 km NW of Almonte, Hwy 49, Burnt Land, Alvar Prov. Park, Almonte, 45.255 -76.14, 29.v.2008, Goulet & Fernandez, Voucher Code: CAM0320, CAM0322, CAM0324, CAM0325, CAM0327, CAM0328, CAM0329.

#### Microplitis jft21

Taxon classificationAnimaliaHymenopteraBraconidae

##### Distribution.

NEA.

##### Notes.

This species corresponds in BOLD to BIN BOLD:AAE8603, with all specimens collected in eastern North America.

##### Material examined.

Ontario, Ottawa, city garden, 45.356 -75.707, 30.v.2007, H. Goulet, Voucher Code: CAM0228, CAM0231; 30.vii.2007, H. Goulet, Voucher Code: CAM0184.

#### Microplitis jft23

Taxon classificationAnimaliaHymenopteraBraconidae

##### Distribution.

NEA.

##### Notes.

This species corresponds in BOLD to BIN BOLD:ABZ3353, with all specimens collected in southern Ontario.

##### Material examined.

Ontario, Ottawa, city garden, 45.356 -75.707, 30.v.2007, H. Goulet, Voucher Code: CAM0221.

#### Microplitis jft25

Taxon classificationAnimaliaHymenopteraBraconidae

##### Distribution.

NEA.

##### Notes.

This species corresponds in BOLD to BIN BOLD:AAF5357, with all specimens collected in southern Ontario.

##### Material examined.

Ontario, Woodlawn, 45.375400 -76.082700, 8.ix.2008, L. Masner, Voucher Code: MIC000619.

#### Microplitis jft34

Taxon classificationAnimaliaHymenopteraBraconidae

##### Distribution.

NEA.

##### Notes.

This species corresponds in BOLD to BIN BOLD:AAK6461, with all specimens collected in Canada.

##### Material examined.

Ontario, Ottawa, city garden, 45.356 -75.707, 30.vii-10.viii.2007, H. Goulet, Voucher Code: WMIC0221.

#### Microplitis jft47

Taxon classificationAnimaliaHymenopteraBraconidae

##### Distribution.

NEA.

##### Notes.

This species corresponds in BOLD to BIN BOLD:AAX4115, with all specimens collected in southern Ontario.

##### Material examined.

Ontario, 5 km NW of Almonte, Hwy 49, Burnt Land, Alvar Prov. Park, Almonte, 45.255 -76.14, 29.v.2008, Goulet & Fernandez, Voucher Code: CAM0326.

#### Microplitis jft50

Taxon classificationAnimaliaHymenopteraBraconidae

##### Distribution.

NEA.

##### Notes.

This species corresponds in BOLD to BIN BOLD:AAX4123, with all specimens collected in eastern Canada.

##### Material examined.

Ontario, mixed forest, 45.235 -75.624, 19-29.vi.2007, A. Bennett, Voucher Code: CAM0550.

#### Microplitis jft52

Taxon classificationAnimaliaHymenopteraBraconidae

##### Distribution.

NEA.

##### Notes.

This species corresponds in BOLD to BIN BOLD:AAH6443, with all specimens collected in eastern Canada.

##### Material examined.

Ontario, North Gower to Smith Falls, 1 km N of Rd 6 & Montague Boundary Rd, 45.033 -75.9, 15.vi.2004, Bennett & Barnes, Voucher Code: HYM00001235, HYM00001236.

#### Microplitis jft53

Taxon classificationAnimaliaHymenopteraBraconidae

##### Distribution.

NEA.

##### Notes.

This species corresponds in BOLD to BIN BOLD:AAE8502, with all specimens collected in North America.

##### Material examined.

Ontario, 5 km NW of Almonte, Hwy 49, Burnt Land, Alvar Prov. Park, Almonte, 45.255 -76.14, 29.v.2008, Goulet & Fernandez, Voucher Code: CAM0323, CAM0330; Ottawa, city garden, 45.356 -75.707, 30.v.2007, H. Goulet, Voucher Code: CAM0214.

#### Microplitis jft56

Taxon classificationAnimaliaHymenopteraBraconidae

##### Distribution.

NEA.

##### Notes.

This species corresponds in BOLD to BIN BOLD:AAH3516, with all specimens collected in southern Ontario.

##### Material examined.

Ontario, Ottawa, city garden, 45.356 -75.707, 30.v.2007, H. Goulet, Voucher Code: CAM0217, CAM0219.

#### Microplitis jft60

Taxon classificationAnimaliaHymenopteraBraconidae

##### Distribution.

NEA.

##### Notes.

This species corresponds in BOLD to BIN BOLD:AAE8461, with all specimens collected in North America.

##### Material examined.

Ontario, mixed forest, 45.235 -75.624, 29.vi-16.vii.2007, A. Bennett, Voucher Code: CAM0553; Ottawa, city garden, 45.356 -75.707, 10.viii-1.ix.2007, H. Goulet, Voucher Code: WMIC0202, WMIC0213.

#### Microplitis nr. autographae

Taxon classificationAnimaliaHymenopteraBraconidae

##### Distribution.

NEA.

##### Material examined.

Ontario, Ottawa, 45.406633 -75.701408, 27.vii.1947, W.R.M. Mason, Voucher Code: CNC483613.

#### Pholetesor bedelliae

Taxon classificationAnimaliaHymenopteraBraconidae

(Viereck, 1911)

Fig. 31

##### Distribution.

NEA, NEO.

##### Material examined.

Ontario, 5 km NW of Almonte, Hwy 49, Burnt Land, Alvar Prov. Park, Almonte, 45.255 -76.14, 29.v.2008, Goulet & Fernandez, Voucher Code: CAM0437; Kinburn, 45.391853 -76.188798, 9.iii.1958, Freeman & Lewis, Voucher Code: CNC482372; Ottawa, city garden, 45.356 -75.707, 30.v.2007, H. Goulet, Voucher Code: CAM0278.

**Figure 31.**
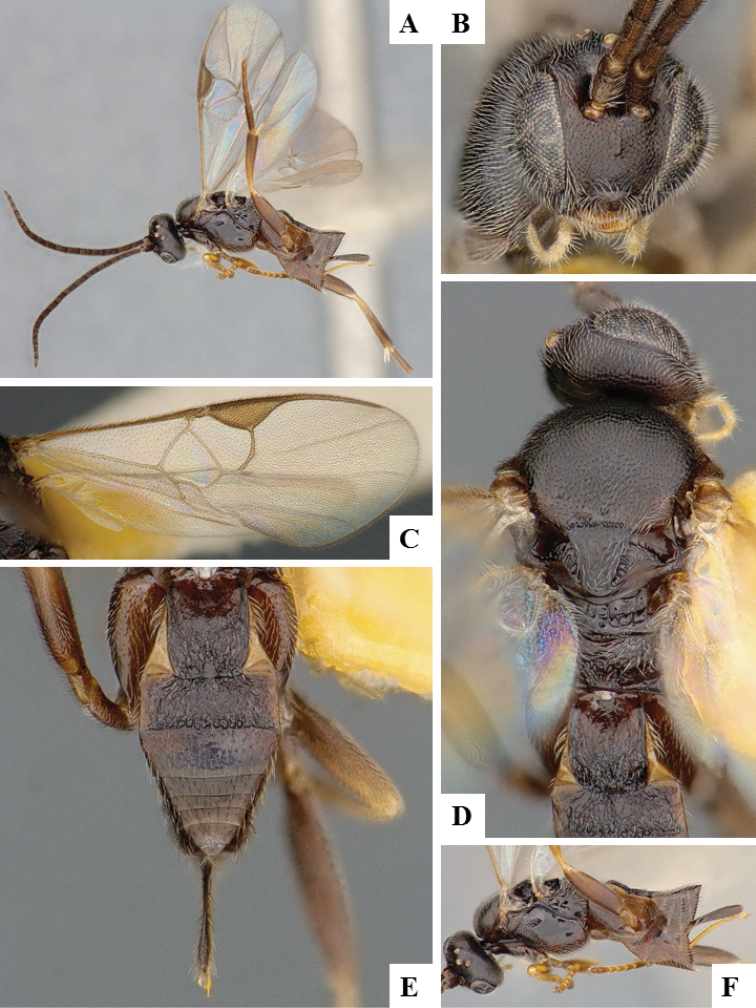
*Pholetesor bedelliae*. **A** Habitus, lateral **B** Head, frontal **C** Wings **D** Head and mesosoma, dorsal **E** Metasoma, dorsal **F** Mesosoma and metasoma, lateral.

#### Pholetesor masoni

Taxon classificationAnimaliaHymenopteraBraconidae

Whitfield, 2006

##### Distribution.

NEA, NEO.

##### Material examined.

Ontario, Aylmer West, 45.395345 -75.844876, Malaise trap, 30.v.1972, Voucher Code: CNC482374, CNC482375, CNC482376, CNC482377, CNC482378, CNC482379, CNC482380, CNC482381, CNC482382, CNC482383, CNC482384, CNC482385, CNC482386, CNC482387; Ottawa, 45.356083 -75.706933, 25.v.1944, McDunnough, J. , Voucher Code: CNC309874; 45.406631 -75.701407, 25.vii.1957, J. E. H. Martin, Voucher Code: CNC482373.

#### Pholetesor nanus

Taxon classificationAnimaliaHymenopteraBraconidae

(Reinhard, 1880)

##### Distribution.

NEA, PAL.

##### Notes.

This is the first record of this species for the Nearctic region.

##### Material examined.

Ontario, Bells Corners, 45.322133 -75.833301, 25.vii.1962, Miller, C.D. Voucher Code: CNC309876.

#### Pholetesor ornigis

Taxon classificationAnimaliaHymenopteraBraconidae

(Weed, 1887)

Fig. 32

##### Distribution.

NEA.

##### Material examined.

Ontario, 5 km NW of Almonte, Hwy 49, Burnt Land, Alvar Prov. Park, Almonte, 45.2549 -76.14, 29.v.2008, Goulet & Fernandez, Voucher Code: CAM0424, CAM0433; Aylmer, 45.395345 -75.844876, 18.vii.1960, C. D. Miller, Voucher Code: CNC482388, CNC482389, CNC482390; Bells Corners, 45.322247 -75.833249, 2.viii.1962, C. D. Miller, Voucher Code: CNC482396; 7.viii.1960, C. D. Miller, Voucher Code: CNC482395; Constance Bay, 45.486218 -76.073461, 4-5.viii.1955, T. N. Freeman, Voucher Code: CNC483512; 5.viii.1955, T. N. Freeman, Voucher Code: CNC482423, CNC483510, CNC483511; Fitzroy Harbour, 45.471703 -76.214077, 17.viii.1960, C.D. Miller, Voucher Code: CNC483564, CNC483565, CNC483566, CNC483567, CNC483568, CNC483569; 6.vi.1962, Freeman & Lewis, Voucher Code: CNC483571, CNC483572, CNC483573, CNC483574, CNC483575, CNC483576, CNC483577, CNC483578; 8-9.vi.1962, Freeman & Lewis, Voucher Code: CNC483580; 9.vi.1962, Freeman & Lewis, Voucher Code: CNC483579; Kemptville, 45.016409 -75.646449, 12.iii.1962, Voucher Code: CNC483582; 13.iii.1962, Voucher Code: CNC483583; 19.iii.1962, Voucher Code: CNC483584; Ottawa, 45.406631 -75.701407, 18.i.1961, Freeman & Lewis, Voucher Code: CNC482412; 2.vii.1960, J.R. Vockeroth, Voucher Code: CNC483581; 22.i.1961, Freeman & Lewis, Voucher Code: CNC482415, CNC482422; 25.i.1961, Freeman & Lewis, Voucher Code: CNC482410, CNC482411; 3.ii.1961, Freeman & Lewis, Voucher Code: CNC482413; 5.ii.1961, Freeman & Lewis, Voucher Code: CNC482414; Stittsville, 45.258675 -75.921130, 11.vii.1960, Voucher Code: CNC482406; 12.vii.1960, Voucher Code: CNC482405; 13.vii.1960, Voucher Code: CNC482403; 18.vii.1960, Voucher Code: CNC482399, CNC482400, CNC482401, CNC482402; 19.vii.1960, Voucher Code: CNC482404; 20.vii.1960, C. D. Miller, Voucher Code: CNC482397, CNC482398; 22.vii.1960, C.D. Miller, ~, Voucher Code: CNC483570, CNC482407; 23.vii.1960, Voucher Code: CNC482408; 25.vii.1960, Voucher Code: CNC482409; Woodlawn, 45.462683 -76.075555, 14.viii.1962, C. D. Miller, Voucher Code: CNC482419; Quebec, Aylmer, 45.395345 -75.844876, 12.vii.1960, C. D. Miller, Voucher Code: CNC482391, CNC482392, CNC482393, CNC482394; Gatineau Park, 45.60057 -76.042647, 24.v.2007, L. Masner, Voucher Code: HYM00001323; Gatineau, 45.478218 -75.701346, 2.viii.1960, C. D. Miller, Voucher Code: CNC482426, CNC483509; 29.vii.1960, C. D. Miller, Voucher Code: CNC483507; 3-8.viii.1960, C. D. Miller, Voucher Code: CNC482427; 3.viii.1960, C. D. Miller, Voucher Code: CNC482428, CNC482430, CNC483506; 31.vii.1960, C. D. Miller, Voucher Code: CNC482433, CNC483508; 4.viii.1960, C. D. Miller, Voucher Code: CNC482429; 8.viii.1960, C. D. Miller, Voucher Code: CNC482425, CNC482431, CNC482432; 9.viii.1960, C. D. Miller, Voucher Code: CNC482424; Hull, 45.428550 -75.714554, 10.viii.1962, C. D. Miller, Voucher Code: CNC482451; 11.viii.1962, C. D. Miller, Voucher Code: CNC483505; 11.viii.1965, C. D. Miller, Voucher Code: CNC483549; 12.viii.1960, C. D. Miller, Voucher Code: CNC483532, CNC483533; 13.v.1963, C. D. Miller, Voucher Code: CNC483521, CNC483522, CNC483523; 13.viii.1962, C. D. Miller, Voucher Code: CNC482450, CNC483501; 14.vii.1962, C. D. Miller, Voucher Code: CNC483518; 14.viii.1962, C. D. Miller, Voucher Code: CNC483519, CNC483520; 15.v.1963, C. D. Miller, Voucher Code: CNC482448; 16.viii.1962, C. D. Miller, Voucher Code: CNC483498; 17.vii.1963, C. D. Miller, Voucher Code: CNC483513, CNC483514, CNC483515, CNC483516, CNC483517; 17.viii.1962, C. D. Miller, Voucher Code: CNC483497; 2.viii.1960, C. D. Miller, Voucher Code: CNC483525, CNC483551, CNC483552, CNC483553, CNC483554, CNC483555, CNC483562, CNC483563; 2.viii.1962, C. D. Miller, Voucher Code: CNC482447; 23.viii.1965, C. D. Miller, Voucher Code: CNC483536; 27.vii.1960, C. D. Miller, Voucher Code: CNC483529, CNC483530; 28.vii.1960, C. D. Miller, Voucher Code: CNC483526; 28.viii.1962, C. D. Miller, Voucher Code: CNC483503; 29.vii.1960, C. D. Miller, Voucher Code: CNC483528; 3.viii.1960, C. D. Miller, Voucher Code: CNC483556, CNC483557, CNC483558, CNC483559; 3.viii.1962, C. D. Miller, Voucher Code: CNC482440, CNC482441, CNC482442, CNC482449, CNC483543, CNC483544; 30.vii.1962, C. D. Miller, Voucher Code: CNC483502; 30.viii.1962, C. D. Miller, Voucher Code: CNC482437, CNC482438, CNC482439, CNC483495, CNC483496, CNC483499, CNC483504; 31.vii.1960, C. D. Miller, Voucher Code: CNC483561; 31.vii.1962, C. D. Miller, Voucher Code: CNC483524; 31.viii.1965, C. D. Miller, Voucher Code: CNC483537, CNC483550; 4.iii.1957, Freeman & Lewis, Voucher Code: CNC482434, CNC482435; 4.viii.1960, C. D. Miller, Voucher Code: CNC483527; 4.viii.1965, C. D. Miller, Voucher Code: CNC483545; 5.viii.1960, C. D. Miller, Voucher Code: CNC483531, CNC483560; 6.viii.1965, C. D. Miller, Voucher Code: CNC483535, CNC483546, CNC483547, CNC483548; 7.viii.1962, C. D. Miller, Voucher Code: CNC482443, CNC482444, CNC482445, CNC482446, CNC483538, CNC483539, CNC483540, CNC483541, CNC483542; 8.viii.1960, C. D. Miller, Voucher Code: CNC483534; 8.xi.1960, T. N. Freeman, Voucher Code: CNC482436; 9-30.viii.1962, C. D. Miller, Voucher Code: CNC483500; Val Tetreau, 45.419217 -75.745275, 18.i.1961, Freeman & Lewis, Voucher Code: CNC482420; 27.i.1961, Freeman & Lewis, Voucher Code: CNC482421; 8.viii.1960, C. D. Miller, Voucher Code: CNC482416, CNC482417, CNC482418.

**Figure 32.**
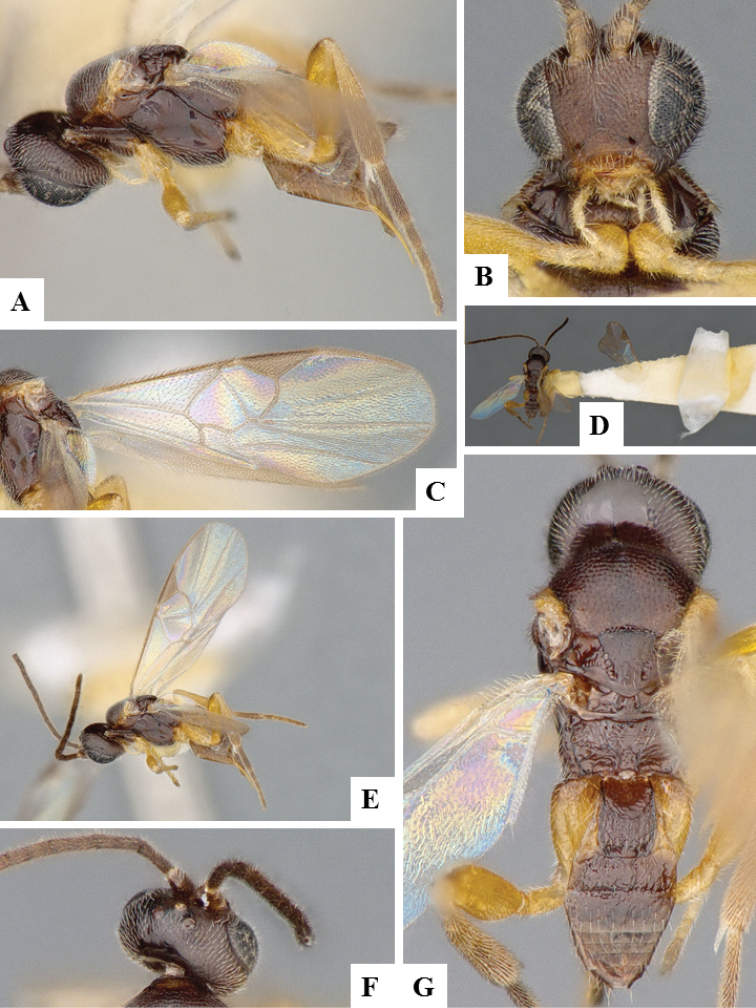
*Pholetesor ornigis*. **A** Head and mesosoma, lateral **B** Head, frontal **C** Wings **D** Glued specimen and cocoon **E** Habitus, lateral **F** Head, dorsal **G** Habitus, dorsal.

#### Pholetesor pinifoliellae

Taxon classificationAnimaliaHymenopteraBraconidae

Whitfield, 2006

##### Distribution.

NEA.

##### Material examined.

Ontario, Ottawa, 45.356083 -75.706933, 28.vii.1950, Voucher Code: CNC Type 19982 and one additional specimen without any voucher code.

#### Pholetesor rhygoplitoides

Taxon classificationAnimaliaHymenopteraBraconidae

Whitfield, 2006

Fig. 33

##### Distribution.

NEA.

##### Material examined.

Ontario, Mer Bleue, 45.393578 -75.512127, 19.vii.1963, Chillcott, J.G, Voucher Code: CNC309878; 45.393593 -75.512138, 18.vii.1963, J. G. Chillcott, Voucher Code: CNCHYM03189.

**Figure 33.**
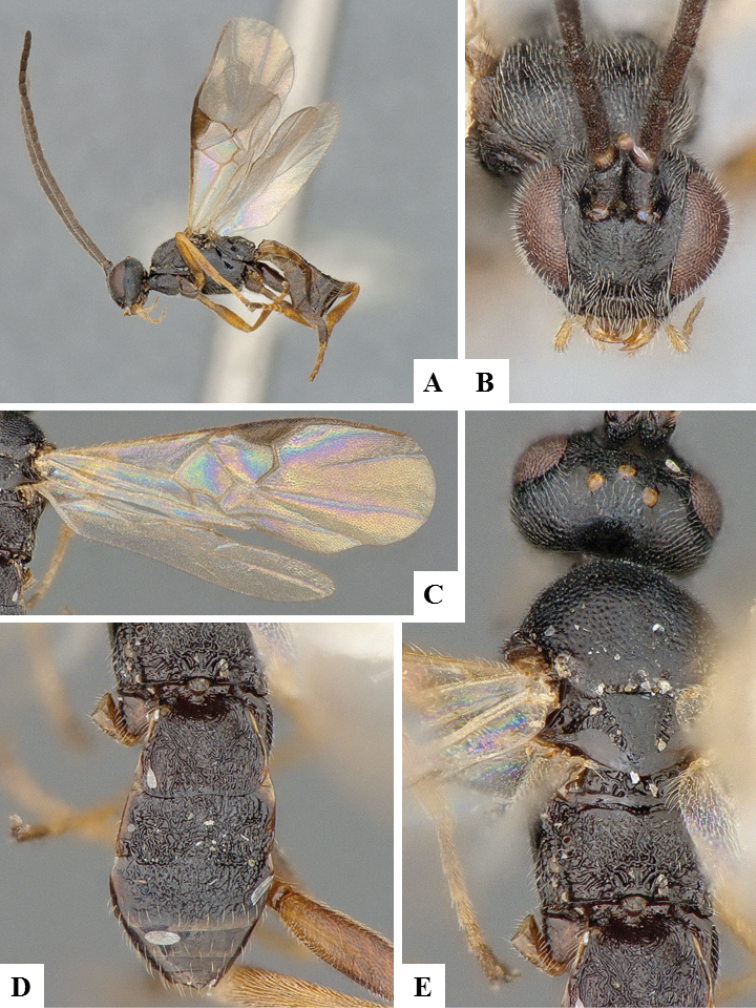
*Pholetesor rhygoplitoides*. **A** Habitus, lateral **B** Head, frontal **C** Wings **D** Metasoma, dorsal **E** Head and mesosoma, dorsal.

#### Pholetesor salicifoliellae

Taxon classificationAnimaliaHymenopteraBraconidae

Mason, 1959

Fig. 34

##### Distribution.

NEA.

##### Material examined.

Ontario, 5 km NW of Almonte, Hwy 49, Burnt Land, Alvar Prov. Park, Almonte, 45.2549 -76.14, 29.v.2008, Goulet & Fernandez, Voucher Code: CAM0343, CAM0355, CAM0362, CAM0420, CAM0425.

**Figure 34.**
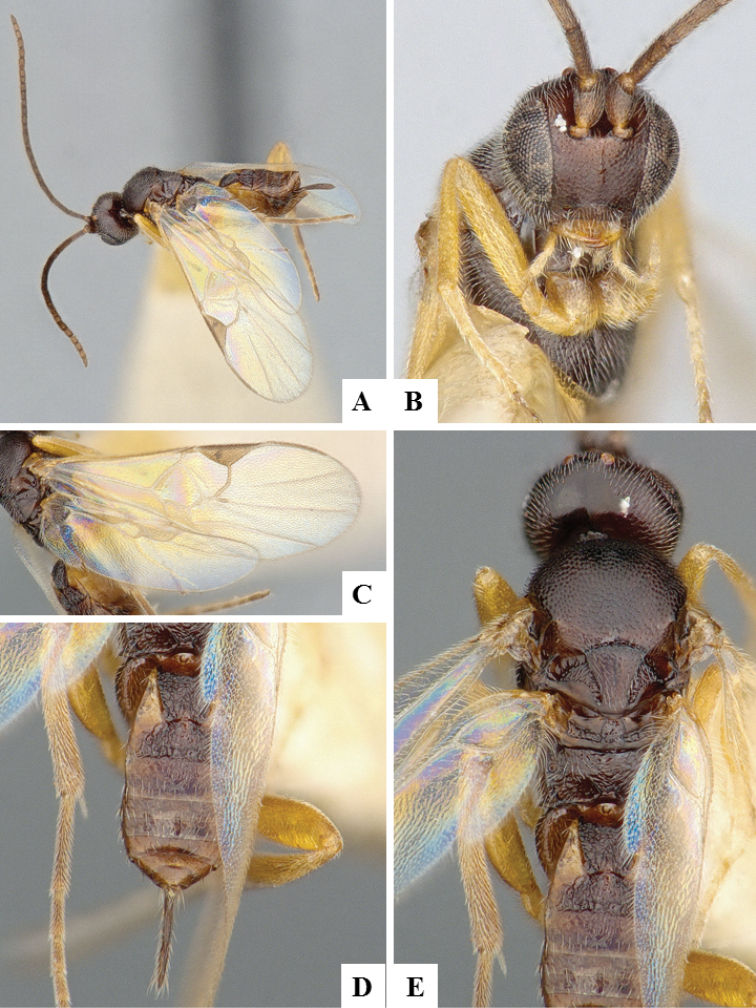
*Pholetesor salicifoliellae*. **A** Habitus, lateral **B** Head, frontal **C** Wings **D** Metasoma, dorsal **E** Head and mesosoma, dorsal.

#### Pholetesor viminetorum

Taxon classificationAnimaliaHymenopteraBraconidae

(Wesmael, 1837)

##### Distribution.

NEA, PAL.

##### Material examined.

Ontario, 5 km NW of Almonte, Hwy 49, Burnt Land, Alvar Prov. Park, Almonte, 45.255 -76.14, 29.v.2008, Goulet & Fernandez, Voucher Code: CAM0365, CAM0414, CAM0415, CAM0416, CAM0417, CAM0418, CAM0419, CAM0421, CAM0422, CAM0423, CAM0426, CAM0427, CAM0428, CAM0429, CAM0430, CAM0431, CAM0432, CAM0434, CAM0435, CAM0438.

#### Pholetesor jft03

Taxon classificationAnimaliaHymenopteraBraconidae

##### Distribution.

NEA.

##### Notes.

This species corresponds in BOLD to BIN BOLD:ABZ3171, with all specimens collected in eastern North America.

##### Material examined.

Ontario, Ottawa, city garden, 45.3561 -75.7069, 30.vii-10.viii.2007, H. Goulet, Voucher Code: CAM0900.

#### Pholetesor jft04

Taxon classificationAnimaliaHymenopteraBraconidae

##### Distribution.

NEA.

##### Notes.

This species corresponds in BOLD to BIN BOLD:AAE0349, with all specimens collected in eastern Canada.

##### Material examined.

Ontario, Ottawa, city garden, 45.3561 -75.7069, 8-16.vi.2007, H. Goulet, Voucher Code: CAM0896; 45.3561 -75.707, 23.vii.2007, H. Goulet, Voucher Code: CAM0283, CAM0284; 30.v.2007, H. Goulet, Voucher Code: CAM0279; 8.vi.2007, H. Goulet, Voucher Code: CAM0285; 45.356100 -75.706900, 30.vii-10.viii.2007, H. Goulet, Voucher Code: CAM0901.

#### Pholetesor jft05

Taxon classificationAnimaliaHymenopteraBraconidae

##### Distribution.

NEA.

##### Notes.

This species corresponds in BOLD to BIN BOLD:AAD5198, with all specimens collected in North America.

##### Material examined.

Ontario, Ottawa, city garden, 45.3561 -75.707, 1.ix.2007, H. Goulet, Voucher Code: CAM0281; 16.vi.2007, H. Goulet, Voucher Code: CAM0002, CAM0004; 23.vii.2007, H. Goulet, Voucher Code: CAM0282.

#### Pholetesor jft09

Taxon classificationAnimaliaHymenopteraBraconidae

##### Distribution.

NEA.

##### Notes.

This species corresponds in BOLD to BIN BOLD:AAA8732, with all specimens collected in North America.

##### Material examined.

Ontario, Ottawa, city garden, 45.356100 -75.707000, 8.ix.2007, H. Goulet, Voucher Code: CAM0100.

#### Pholetesor jft12

Taxon classificationAnimaliaHymenopteraBraconidae

##### Distribution.

NEA.

##### Notes.

This species corresponds in BOLD to BIN BOLD:AAB5026, with all specimens collected in Canada.

##### Material examined.

Ontario, Ottawa, city garden, 45.356100 -75.706900, 23-30.vii.2007, H. Goulet, Voucher Code: CAM0899.

#### Protapanteles paleacritae

Taxon classificationAnimaliaHymenopteraBraconidae

(Riley, 1881)

Fig. 35

##### Distribution.

NEA.

##### Material examined.

Ontario, Carp, 45.349117 -76.041225, 23.vii.1942, Voucher Code: CNC483585; Greely, 45.259544 -75.568696, 25.vi.1946, Voucher Code: CNC483586.

**Figure 35.**
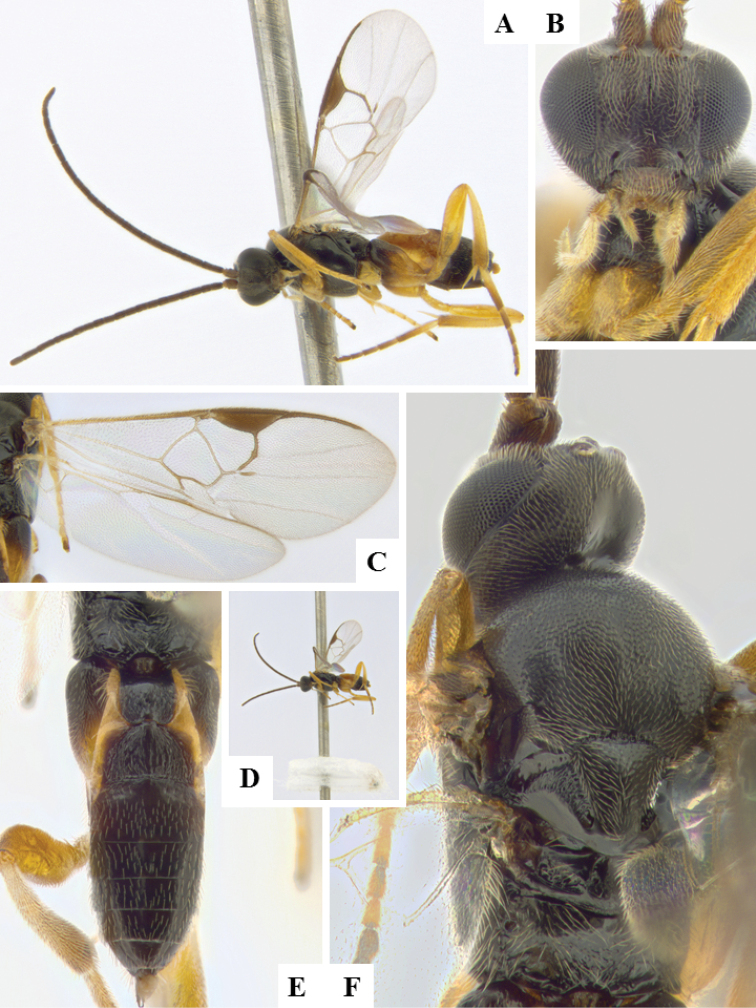
*Protapanteles paleacritae*. **A** Habitus, lateral **B** Head, frontal **C** Wings **D** Glued specimen and cocoon **E** Metasoma, dorsal **F** Head and mesosoma, dorsal.

#### Protapanteles phlyctaeniae

Taxon classificationAnimaliaHymenopteraBraconidae

(Muesebeck, 1929)

Fig. 36

##### Distribution.

NEA.

##### Material examined.

Ontario, Ottawa, 45.3825 -75.7137, 18.vii.1950, E. Munroe, Voucher Code: CNCHYM03246.

**Figure 36.**
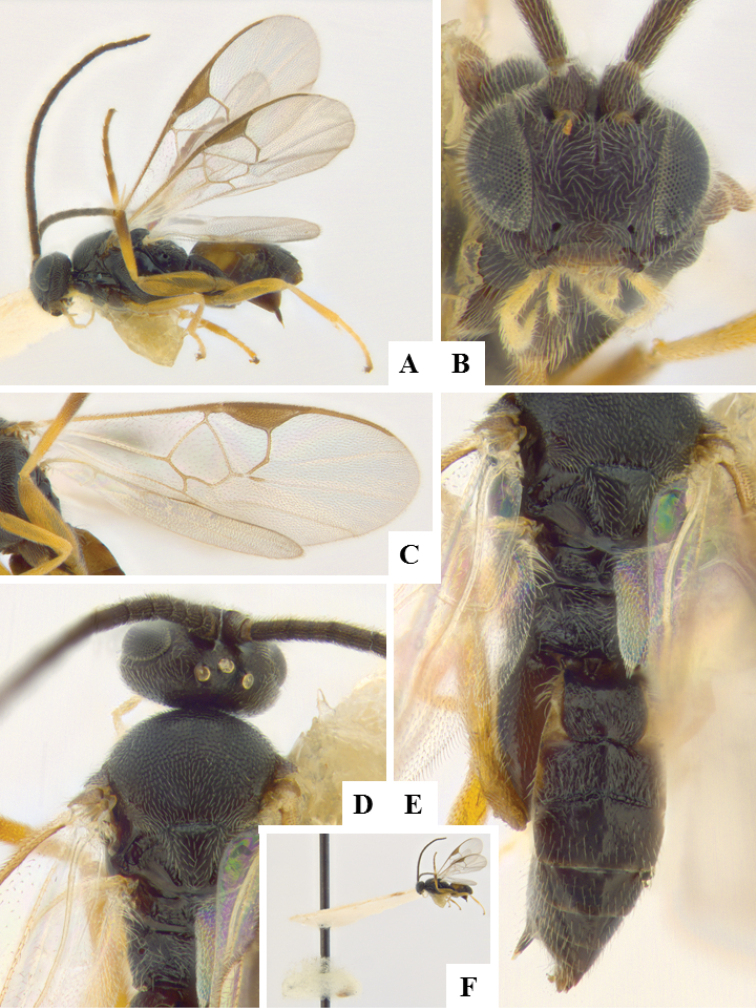
*Protapanteles phlyctaeniae*. **A** Habitus, lateral **B** Head, frontal **C** Wings **D** Head and mesosoma, dorsal **E** Mesosoma (partially) and metasoma, dorsal **F** Glued specimen and cocoon.

#### Protapanteles cf. anchisiades

Taxon classificationAnimaliaHymenopteraBraconidae

##### Distribution.

NEA, PAL.

##### Notes.

This species seems to be part of a complex related to *Protapanteles anchisiades* (Nixon, 1973), from both European and North American specimens. For the time being is left as an undescribed species, until more studies of the Holarctic fauna are carried out. This species corresponds in BOLD to BIN BOLD:AAA7147.

##### Material examined.

Ontario, Ottawa, city garden, 45.356100 -75.706900, 23.vii.2009, L. Masner, Voucher Code: CNCH0461.

#### Protapanteles jft02

Taxon classificationAnimaliaHymenopteraBraconidae

##### Distribution.

NEA, PAL.

##### Notes.

This species seems to be part of a complex related to *Protapanteles anchisiades* (Nixon, 1973), from both European and North American specimens. For the time being is left as an undescribed species, until more studies of the Holarctic fauna are carried out. This species corresponds in BOLD to BIN BOLD:AAA7147.

##### Material examined.

Ontario, Ottawa, city garden, 45.3561 -75.7069, 16.vi.2007, H. Goulet, Voucher Code: HYM00000998; 45.3561 -75.707, 1.ix.2007, H. Goulet, Voucher Code: CAM0269; 1.xi.2007, H. Goulet, Voucher Code: CAM0271; 30.vii.2007, H. Goulet, Voucher Code: CAM0275.

#### Protomicroplitis calliptera

Taxon classificationAnimaliaHymenopteraBraconidae

(Say, 1836)

Fig. 37

##### Distribution.

NEA.

##### Notes.

The status of this species as a potential member of the Species Candidate Lists of COSEWIC was assessed by Fernandez Triana (2014).

**Figure 37.**
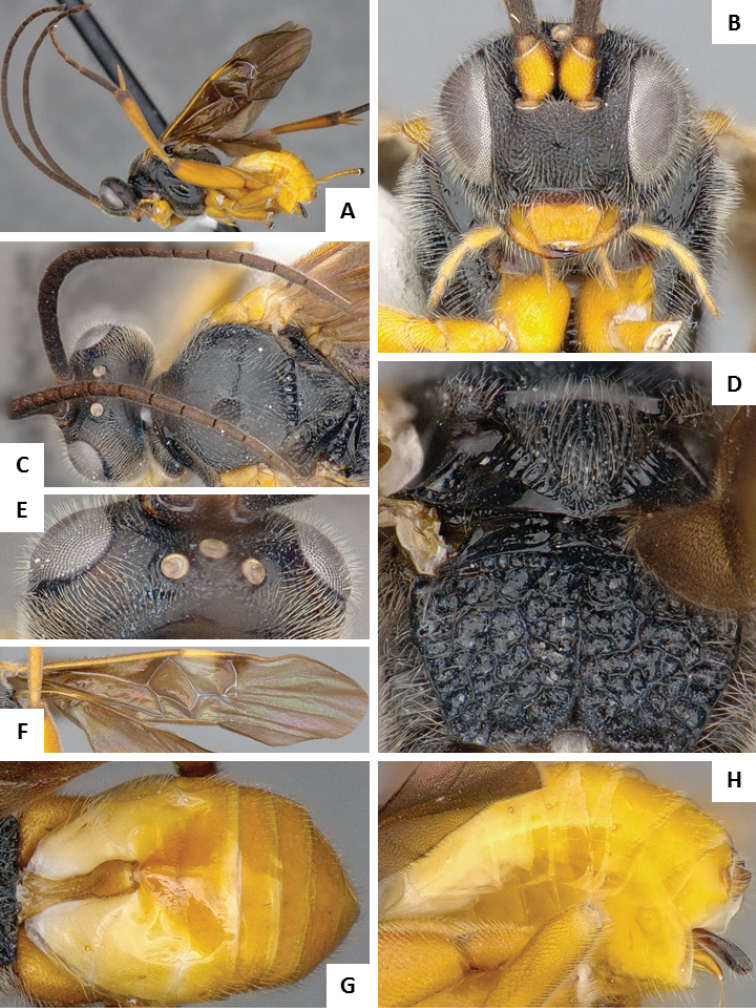
*Protomicroplitis calliptera*. **A** Habitus, lateral **B** Head, frontal **C** Head and mesosoma (partially), dorsal **D** Propodeum **E** Head, dorsal **F** Wings **G** Metasoma, dorsal **H** Metasoma, lateral.

##### Material examined.

Ontario, Stittsville, 45.258675 -75.921130, 27.viii.1963, W.R.M. Mason, Voucher Code: CNC483587; 30.vi.1963, W.R.M. Mason, Voucher Code: CNC483588; 45.258796 -75.92113, 10.ix.1963, W. R. M. Mason, Voucher Code: CNCHYM03255; 45.259039 -75.920958, 10.ix.1963, Mason, W.R.M, Voucher Code: CNC309895.

#### Pseudapanteles gouleti

Taxon classificationAnimaliaHymenopteraBraconidae

Fernandez-Triana, 2010

Fig. 38

##### Distribution.

NEA.

##### Notes.

The status of this species as a potential member of the Species Candidate Lists of COSEWIC was assessed by Fernandez Triana (2014).

##### Material examined.

Ontario, Innisville, 45.054942 -76.250619, 6.viii.1963, W. R. M. Mason, Voucher Code: CNCHYM07228; Ottawa, city garden, 45.356 -75.707, 1.ix.2007, H. Goulet, Voucher Code: CAM0254, CAM0258; 10.viii.2007, H. Goulet, Voucher Code: CAM0255, CAM0256, CAM0257; 23-30.vii.2007, H. Goulet, Voucher Code: CAM0883; 23.vii.2007, H. Goulet, Voucher Code: CAM0251, CAM0252, CAM0253; 30.vii-10.viii.2007, H. Goulet, Voucher Code: CAM0874, CAM0875; 45.3561 -75.7069, 23-30.vii.2007, H. Goulet, Voucher Code: CAM0876; 45.399881 -75.697287, Malaise trap, 13-23.vii.2007, H. Goulet, Voucher Code: CNC23941; Ottawa, 45.356083 -75.706933, malaise trap, 13-23.vii.2007, H. Goulet, , Twp. Nepean, Ont, 45.335400 -75.723784, 10.v-25.viii.1949, Tripp, H.A., Voucher Code: CNC309905; Quebec, Hull, 45.428309 -75.713353, 31.viii.1965, Voucher Code: CNCHYM07230.

**Figure 38.**
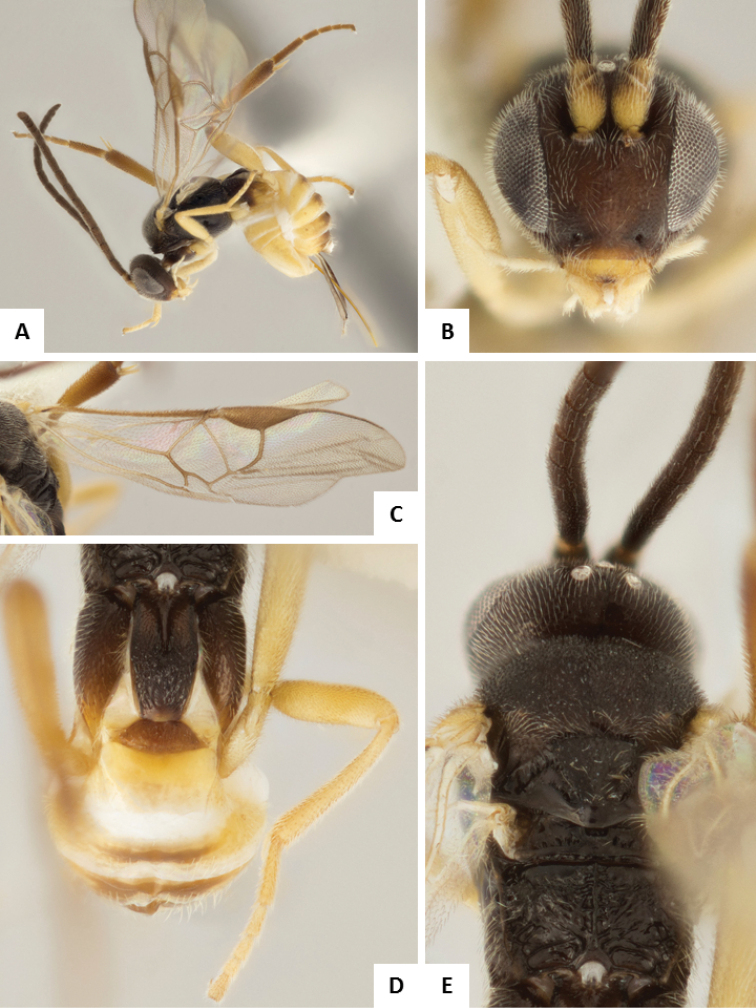
*Pseudapanteles gouleti*. **A** Habitus, lateral **B** Head, frontal **C** Wings **D** Metasoma, dorsal **E** Head and mesosoma, dorsal.

#### Rasivalva rugosa

Taxon classificationAnimaliaHymenopteraBraconidae

(Muesebeck, 1922)

Fig. 39

##### Distribution.

NEA.

##### Material examined.

Quebec, Summit King Mt. Old Chelsea, 45.503586 -75.797942, 351m, 17.viii.1965, Voucher Code: CNCHYM03399.

**Figure 39.**
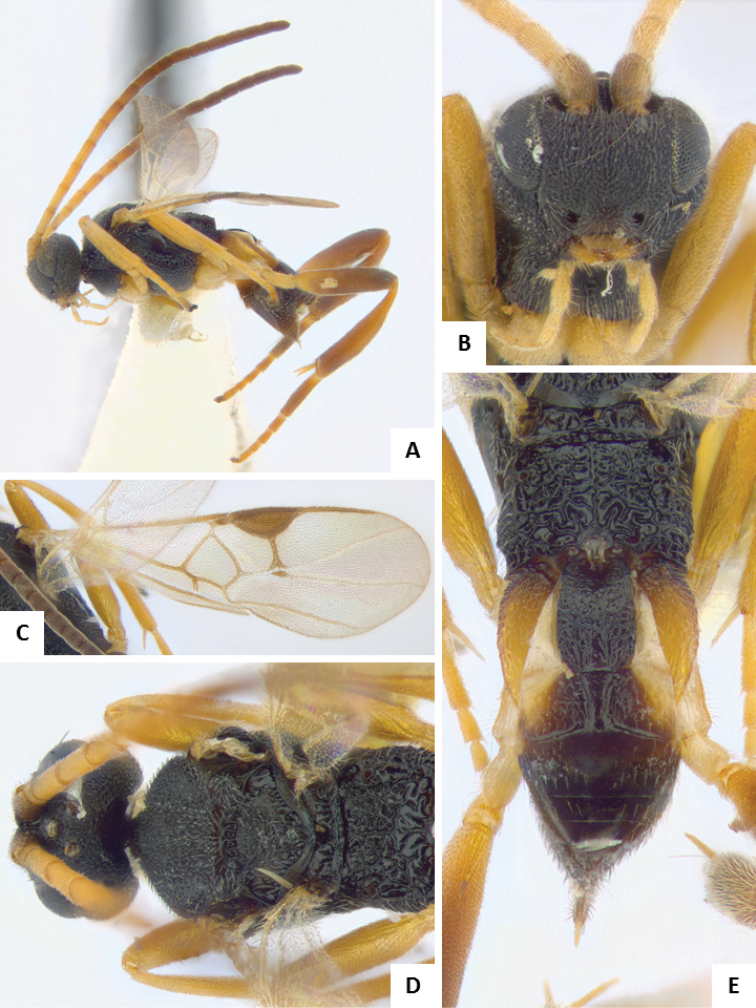
*Rasivalva rugosa*. **A** Habitus, lateral **B** Head, frontal **C** Wings **D** Metasoma, dorsal **E** Head and mesosoma, dorsal.

#### Rasivalva sp.

Taxon classificationAnimaliaHymenopteraBraconidae

##### Distribution.

NEA.

##### Notes.

This species corresponds in BOLD to BIN BOLD:AAH2147, with all specimens collected in southern Ontario.

##### Material examined.

Ontario, Ottawa, city garden, 45.356100 -75.706900, 10.viii-1.ix.2007, H. Goulet, Voucher Code: WMIC0214; 30.vii-10.viii.2007, H. Goulet, Voucher Code: CAM1011.

#### Sathon cinctiformis

Taxon classificationAnimaliaHymenopteraBraconidae

(Viereck, 1911)

Fig. 40

##### Distribution.

NEA.

##### Material examined.

Ontario, Ottawa, Dow’s swamp , 45.419164 -75.709650, 9.vii.1946, G.S. Walley, Voucher Code: CNC309918; Ottawa, Dow’s swamp, 45.394302 -75.704544, 9.vii.1946, G. S. Walley, Voucher Code: CNCHYM03476, CNCHYM03477; Ottawa, 45.406631 -75.701407, 20.vii.1947, W.R.M. Mason, Voucher Code: CNC483601; 20.viii.1947, W.R.M. Mason, Voucher Code: CNC483612; 21.vi.1947, W.R.M. Mason, Voucher Code: CNC483590, CNC483591, CNC483592, CNC483593, CNC483594, CNC483595; 28.vi.1940, O. Peck, Voucher Code: CNC483599; 5.vii.1947, W.R.M. Mason, Voucher Code: CNC483596, CNC483597, CNC483598; 45.406633 -75.701408, 19.vii.1939, O. Peck, Voucher Code: CNC483600; Quebec, Hull, 45.428550 -75.714554, 11.vi.1957, C.D. Miller, Voucher Code: CNC483602, CNC483603, CNC483604, CNC483605, CNC483606, CNC483607, CNC483608, CNC483609; 18.vi.1957, J.G. Chillcott, Voucher Code: CNC483610; 2.viii.1947, W.R.M. Mason, Voucher Code: CNC483611.

**Figure 40.**
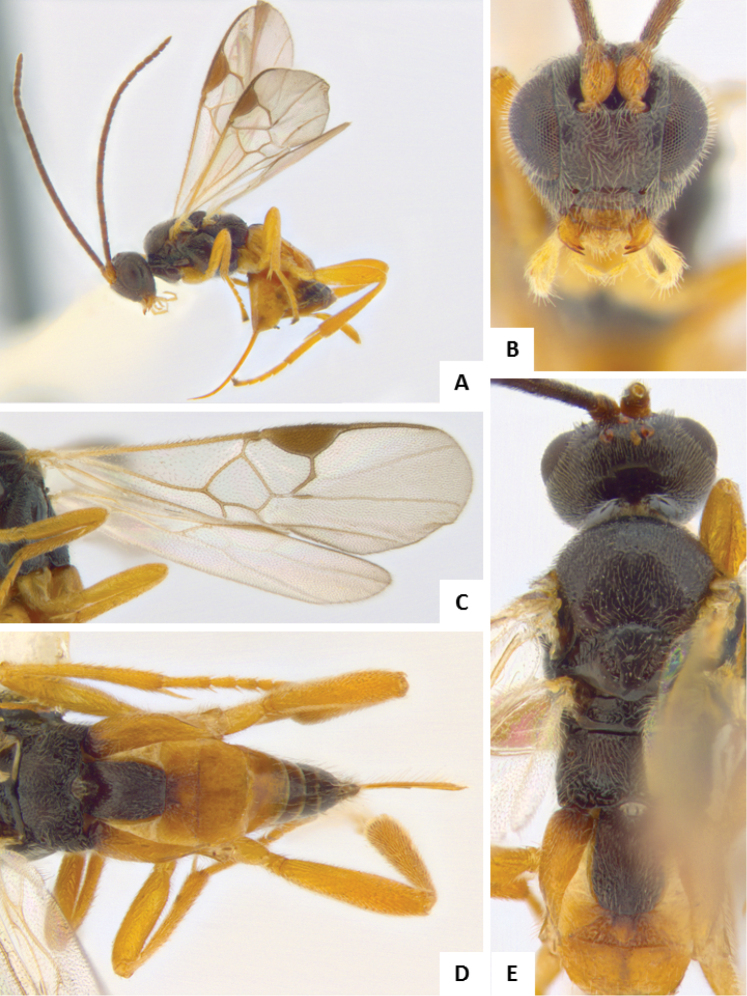
*Sathon cinctiformis*. **A** Habitus, lateral **B** Head, frontal **C** Wings **D** Metasoma, dorsal **E** Head and mesosoma, dorsal.

#### Sathon neomexicanus

Taxon classificationAnimaliaHymenopteraBraconidae

(Muesebeck, 1921)

##### Distribution.

NEA.

##### Material examined.

Ontario, Bells Corners, 45.322133 -75.833303, 7.vii.1943, G.S. Walley, Voucher Code: CNC309924.
