# Corrigenda: A biodiversity hotspot for Microgastrinae (Hymenoptera, Braconidae) in North America: annotated species checklist for Ottawa, Canada

**DOI:** 10.3897/zookeys.927.52153

**Published:** 2020-04-16

**Authors:** Samuel Lauzon, Jose Fernandez-Triana

**Affiliations:** 1 Direction de la recherche forestière, Ministère des Forêts, de la Faune et des Parcs, Québec, Canada Direction de la recherche forestière, Ministère des Forêts, de la Faune et des Parcs du Québec Québec Canada; 2 Canadian National Collection of Insects, Ottawa, Canada Canadian National Collection of Insects Ottawa Canada

## Abstract

In a paper about the biodiversity of Microgastrinae (Hymenoptera: Braconidae) in Ottawa Canada (Fernandez-Triana et al. 2016) some figure captions are incorrect. That includes three cases where the species name shown does not correspond with the actual species being depicted in those figures. To correct those mistakes, we detail below the correct captions for the corresponding figures.

In a paper about the biodiversity of Microgastrinae (Hymenoptera: Braconidae) in Ottawa Canada (Fernandez-Triana et al. 2016) some figure captions are incorrect. That includes three cases where the species name shown does not correspond with the actual species being depicted in those figures. To correct those mistakes, we detail below the correct captions for the corresponding figures.

The caption of Figure 8 (page 17):

**Figure 8.***Apanteleslaricellae*. **A** Habitus, lateral **B** head, frontal **C** wings **D** head and mesosoma (partially), dorsal **E** metasoma, lateral **F** metasoma, dorsal **F** mesosoma, dorsal.

Should read:

**Figure 8.***Apantelespetrovae*. **A** Habitus, lateral **B** head, frontal **C** wings **D** cocoon **E** head and mesosoma (partially), dorsal **F** head and antenna, ventral **G** metasoma, dorsal.

The caption of Figure 9 (page 18):

**Figure 9.***Apantelesmorrisi*. **A** Habitus, lateral **B** head, frontal **C** wings **D** head and mesosoma, dorsal **E** metasoma dorsal **F** head and antenna, dorsal.

Should read:

**Figure 9.***Apanteleslaricellae*. **A** Habitus, lateral **B** head, frontal **C** wings **D** head and mesosoma (partially), dorsal **E** metasoma, lateral **F** metasoma, dorsal **G** mesosoma, dorsal.

The caption of Figure 10 (page 19):

**Figure 10.***Apantelespetrovae*. **A** Habitus, lateral **B** head, frontal **C** wings **D** cocoon **E** head and mesosoma (partially), dorsal **F** head and antenna, ventrally **G** metasoma, dorsal.

Should read:

**Figure 10.***Apantelesmorrisi*. **A** Habitus, lateral **B** head, frontal **C** wings **D** head and mesosoma, dorsal **E** metasoma, dorsal **F** head and antenna, dorsal.

The caption of Figure 12 (page 28):

**Figure 12.***Choerasconsimilis*. **A** Habitus, lateral **B** wings **C** head, frontal **D** metasoma dorsal **F** head and mesosoma, dorsal.

Should read:

**Figure 12.***Choerasconsimilis*. **A** Habitus, lateral **B** wings **C** head, frontal **D** metasoma dorsal **E** head and mesosoma, dorsal.

The caption of Figure 21 (page 48):

**Figure 21.***Dolichogenideaparalechiae*. **A** Habitus, lateral **B** head, frontal **C** wings **D** mesosoma and metasoma (partially), dorsal **E** metasoma, lateral **F** metasoma, dorsal **E** head and mesosoma, dorsal.

Should read:

**Figure 21.***Dolichogenideaparalechiae*. **A** Habitus, lateral **B** head, frontal **C** wings **D** mesosoma and metasoma (partially), dorsal **E** metasoma, lateral **F** metasoma, dorsal **G** head and mesosoma, dorsal.
